# Genetic architectures of brain-related traits are shaped by strong selective constraints

**DOI:** 10.1073/pnas.2609814123

**Published:** 2026-09-01

**Authors:** Huisheng Zhu, Yuval B. Simons, Jeffrey P. Spence, Guy Sella, Jonathan K. Pritchard

**Affiliations:** ^a^ Department of Biology, Stanford University, Stanford, CA 94305; ^b^ Department of Genetics, Stanford University, Stanford, CA 94305; ^c^ Section of Genetic Medicine, University of Chicago, Chicago, IL 60637; ^d^ Department of Human Genetics, University of Chicago, Chicago, IL 60637; ^e^ Institute for Human Genetics, University of California, San Francisco, CA 94158; ^f^ Department of Epidemiology and Biostatistics, University of California, San Francisco, CA 94158; ^g^ Department of Biological Sciences, Columbia University, New York City, NY 10027; ^h^ Program for Mathematical Genomics, Columbia University, New York City, NY 10032

**Keywords:** selection, GWAS, genetic architecture, brain-related traits, psychiatric disorders

## Abstract

Genome-wide association studies (GWAS) have identified hundreds of significant loci for psychiatric disorders, yet the strength of these associations remains modest compared to other human complex traits with similar numbers of hits. Whether this pattern reflects statistical artifacts or real biological differences—and, if the latter, what underlies it—remains unclear. In addition to psychiatric disorders, we find that other traits with functional enrichment in the central nervous system (CNS), whether binary or quantitative, also share similar genetic architectures, characterized by GWAS hits of limited statistical significance and generally higher allele frequencies. In comparing the architecture of binary and quantitative traits, we adjust for statistical power in their respective studies. After this adjustment, we fit an evolutionary model of architecture and show that CNS-enriched traits have large mutational target sizes, with contributing variants and genes experiencing stronger selection than those for other traits. Our findings reveal heterogeneity among complex traits and provide insights into traits that more effectively capture fitness-relevant processes. More broadly, our results suggest that the genetic architectures of complex traits are shaped by the tissues through which these traits are mediated.

## Significance

A central goal of human genetics is to understand how genetic variation shapes complex traits, including height and susceptibility to diseases. Over the past two decades, genome-wide association studies (GWAS) have identified numerous trait-associated genetic variants, but the findings can differ widely across traits. Notably, psychiatric disorders such as schizophrenia—and brain-related traits more broadly—share unusual patterns that lack a formal explanation. Using a population genetics framework, we showed that brain-related traits have large mutational target sizes, with underlying variants shaped by strong selective constraints. Our findings are of both practical and foundational significance, informing the interpretation of past and future GWAS results and contributing to a general understanding of how evolutionary forces shape heritable variation in human populations.

Many, if not most, traits in humans and in other species are genetically complex, with heritable variation spread over myriad variants throughout the genome. Complex traits have been studied for more than a century (1, 2), but only recently has it become possible to systematically dissect the genetic basis of their heritable variation. Over the past two decades, genome-wide association studies (GWAS) in humans have identified tens of thousands of robust associations between genetic variants and a wide array of complex traits (3, 4).

One intriguing observation from GWAS is that the genetic architectures of complex traits can vary widely. (Here, we use the term “architecture” to refer to the number of causal variants and their joint distribution of allele frequencies and effect sizes (4). Often, we focus on the subset of approximately independent variants that exceed genome-wide significance in GWAS.) Notable trait variation in genetic architectures includes differences in the number and magnitude of significant associations detected within the same cohort (5, 6), as well as differences in the distributions of effect sizes (7) and allele frequencies (8) among significant variants.

Understanding the causes and determinants of this variation in architecture is of both practical and foundational importance. From an applied perspective, a trait’s genetic architecture determines the performance of studies aimed at mapping its genetic basis, as well as the accuracy of phenotypic predictions derived from these studies (9–12). From a foundational perspective, the architecture of a trait reflects the evolutionary forces that shape heritable variation in natural populations and largely determines the genetic and phenotypic adaptive response to changes in selection pressures (13).

Recent work from Simons et al. tackled this problem from an evolutionary perspective (14, 15). Motivated by extensive evidence that many quantitative traits are subject to stabilizing selection, with fitness declining with displacement from an optimal trait value (13, 16, 17), and that genetic variation affecting one trait typically affects many others (9, 18, 19), they modeled how selection on variants arises from stabilizing selection in a multidimensional trait space(14). Their model also incorporated the effects of mutation and genetic drift. Given a demographic history and mutation rate per site, the genetic architecture of a trait in this model depends on two parameters and a distribution: the mutational target size (i.e., the number of sites in which a mutation affects the trait), the heritability per site in the target, and the distribution of selection coefficients for newly arising mutations in the target. Fitting this model to the 95 quantitative traits with more than a hundred approximately independent hits in the UK Biobank, they find that this model of pleiotropic stabilizing selection provides a better fit to the architectures of these 95 traits than previous ones (20–25). However, it remains unclear whether this result holds true for complex traits in general.

Several studies have suggested that the genetic architectures of psychiatric disorders and more generally of brain-related traits share characteristics that set them apart from other complex traits. Throughout this manuscript, we use “brain-related” as a shorthand for “CNS-enriched” traits, referring to traits for which genetic variation is inferred to act predominantly through cells of the central nervous system (CNS). We identify such traits using stratified LD score regression (S-LDSC), testing for statistically significant enrichment of SNP heritability in active regulatory regions of CNS cells (26, 27). Brain-related traits are consistently found to be among the most highly polygenic traits examined in GWAS based on a variety of polygenicity measures (25, 28–30), suggesting that they have large mutational target sizes. Additionally, variant effect sizes for brain-related traits have variances that are orders of magnitude smaller than those for other complex traits (31) and were well approximated by a single normal distribution, whereas those for other complex traits typically required a mixture of two or more normals with different scales (32). These findings suggest that brain-related traits arise from a large number of causal variants with relatively evenly distributed contributions to phenotypic variance.

Another consideration is that the traits Simons et al. examined were all quantitative; should we even expect complex diseases and disorders to share similar properties in their genetic architectures? Intuition might suggest that genetic variation affecting these traits would be under directional selection, namely, that alleles that decrease the risk of disease would be selected for and alleles that increase this risk would be selected against. In contrast, the pleiotropic stabilizing selection model postulates that when mutations arise, they are always selected against, and for any given selection coefficient, they are equally likely to increase or decrease liability. Several recent studies support the latter view, indicating that, at least for common genetic variation, the architecture of complex diseases and disorders is predominantly shaped by pleiotropic stabilizing selection (7, 33, 34). These findings therefore suggest that the Simons et al. model may extend to these binary traits.

Thus, we wanted to test whether the Simons et al. model fits brain-related traits, including psychiatric disorders, and to explore how, and whether, these traits differ from other complex traits. Do brain-related traits have unique genetic architectures? If so, what are the evolutionary factors that set brain-related traits apart? Here we set out to answer these questions.

We analyzed GWAS results across a wide range of complex traits and found that brain-related traits have unique features, with significant hits narrowly exceeding the significance threshold and having higher minor allele frequencies (MAF). After adjusting for statistical power and fitting the Simons et al. model, we conclude that psychiatric disorders, along with other brain-related quantitative traits, have large mutational target sizes, and their underlying variants and genes are experiencing stronger selection.

## Results

**Fig. 1.**
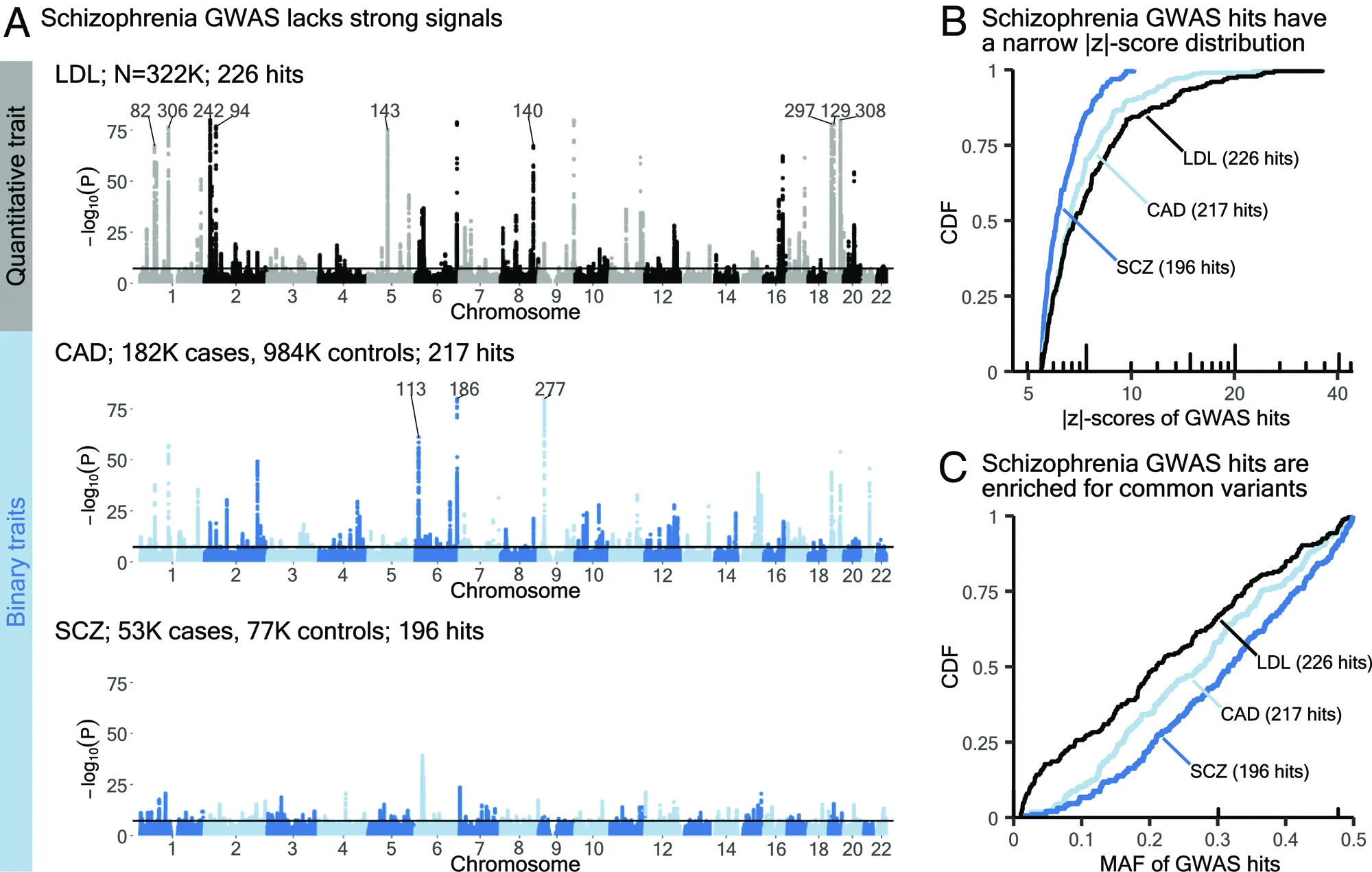
Schizophrenia GWAS hits narrowly exceed the significance threshold. (*A*) Manhattan plots for three example traits. The y-axes of all three plots are restricted to the range [0,80]. Significant associations beyond this range are labeled with the most significant *P*-value. Independent hits are obtained from GCTA-COJO (38), and hits with MAF below 1%, in the HLA region, or of low imputation quality are excluded (*Materials and Methods*). (*B*) Cumulative distributions of COJO hit absolute *z*-scores. (*C*) Cumulative distributions of COJO hit MAF. CAD: coronary artery disease; SCZ: schizophrenia.

Substantial variation in genetic architecture revealed by GWAS can be exemplified with three contrasting traits: a non-brain-related quantitative trait—low-density lipoprotein cholesterol (LDL) (35); a non-brain-related complex disease—coronary artery disease (36); and a brain-related complex disorder—schizophrenia (37). While these GWAS yield similar numbers of approximately independent hits (*Materials and Methods*), the significant associations for schizophrenia surpass the genome-wide significance threshold by only a small margin (Fig. 1*A*).

Differences in strength of significant associations are more readily apparent in the cumulative distributions (CDFs) of |z|-scores of their approximately independent hits (Fig. 1*B*). Schizophrenia, which lacks strong signals, has a steeper CDF curve. When we instead plot the cumulative distributions of MAF, a new piece of information emerges: Schizophrenia GWAS hits are generally higher frequency (Fig. 1*C*). The corresponding density plots for Fig. 1 *B* and *C* are provided in *SI Appendix*, Fig. S6.

### The Distinct Genetic Architectures of Brain-Related Traits.

Next, we asked whether these differences in architecture are seen more generally between brain-related and other complex traits. To this end, we considered the 151 quantitative traits in the UK Biobank with at least 20 approximately independent hits and SNP heritability above 5%, as well as 13 human complex diseases and disorders with publicly available case–control GWAS summary statistics that meet these same criteria Dataset S1; *SI Appendix*, Table S1; and *Materials and Methods*).

**Fig. 2.**
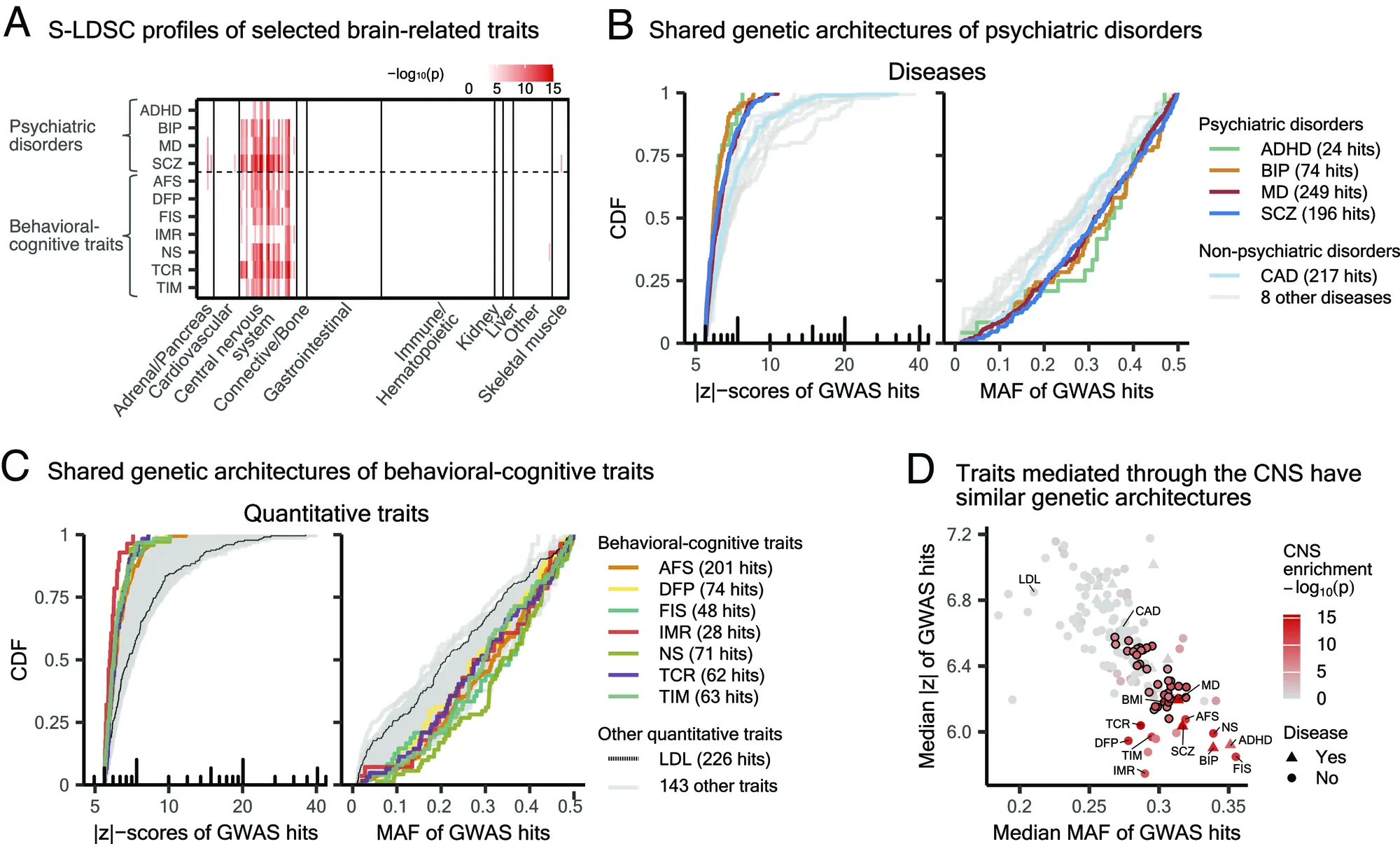
Traits with functional enrichment in the CNS share similar genetic architectures. (*A*) Functional enrichment analyses with S-LDSC (26, 27) on selected traits. Each row consists of 220 cell types from 10 categories, colored by $-\log_{10}\left(p\right)$ for the coefficient *τ* if significant after Bonferroni correction. The gradient scale is bounded above at 15. Results on all other traits are in *SI Appendix*, Figs. S7–S9. (*B*) Contrasting the GWAS hits for psychiatric disorders with those of nine other complex diseases. The full list of diseases and disorders can be found in *SI Appendix*, Table S1. (*C*) Contrasting the GWAS hits for behavioral-cognitive traits with those of 144 other quantitative traits. The full list of quantitative traits is in Dataset S1. (*D*) GWAS hits of traits enriched for the CNS have higher median MAF and lower median |z|-score. Each trait included in this study is colored by the combined *P*-value for CNS enrichment. The points corresponding to the 36 body composition–related traits are outlined with black borders. ADHD: attention deficit hyperactivity disorder; BIP: bipolar disorder; MD: major depression; AFS: age first had sexual intercourse; DFP: duration to first press of snap-button in each round; FIS: fluid intelligence score; IMR: number of incorrect matches in round; NS: neuroticism score; TCR: time to complete round; TIM: mean time to correctly identify matches; and BMI: body mass index.

To classify traits as brain-related or non-brain-related, we first used S-LDSC to obtain *P*-values for the enrichment of SNP heritability in CNS cell types. We then combined the *P*-values across CNS cell types using the aggregate Cauchy association test (ACAT) (39) to quantify overall CNS enrichment (*Materials and Methods*). This yielded 50 brain-related quantitative traits, including seven behavioral-cognitive traits, and 101 non-brain-related quantitative traits (*SI Appendix*, Figs. S7 and S8). Similarly, we identified four brain-related binary traits—all of which were psychiatric disorders—and nine non–brain-related diseases (*SI Appendix*, Fig. S9). Among brain-related traits, psychiatric disorders and behavioral-cognitive traits show the strongest CNS enrichment, and all 11 of these traits are shown in Fig. 2*A*.

As in the schizophrenia example, we find that GWAS hits for brain-related traits, including both quantitative and binary traits, generally span a narrower range of statistical significance and have higher MAF compared with other complex traits (Fig. 2 *B* and *C*). The corresponding density plots for Fig. 2 *B* and *C* are provided in *SI Appendix*, Fig. S10. By contrast, neurological diseases—including Alzheimer’s disease, Parkinson’s disease, and multiple sclerosis—are not classified as brain-related by our pipeline, and do not exhibit the genetic architecture observed for brain-related traits (*SI Appendix*, Fig. S11).

At a fixed sample size, the expected strength of association for a given variant *i* in GWAS is determined by its MAF $p_{i}$ and squared effect size $\beta_{i}^{2}$; more precisely, $z_{i}^{2}\propto 2p_{i}\left(1-p_{i}\right)\beta_{i}^{2}$ in expectation (see, e.g., SI Appendix of ref. 40). Despite being enriched for common variants, GWAS hits for brain-related traits show narrower distributions of |z|-scores. This is explained by their smaller squared effect sizes in standardized units relative to other complex traits (*SI Appendix*, Fig. S12), echoing previous findings (31, 32).

The narrow distributions of |z|-scores, elevated MAF, and small effect sizes place the genetic architectures of brain-related traits at an extreme among complex traits (Fig. 2*D* and *SI Appendix*, Figs. S13 and S14). Traits showing enrichment in other tissues, including the adrenal gland and pancreas, kidney, and skeletal muscle, also cluster in this parameterization of architecture, although such enrichment signals can also arise from correlations in annotations across cell types. Nonetheless, none of these tissue-enriched groups are separated from other traits as strongly as those showing CNS enrichment. If a trait is mediated by the CNS as well as cells in other tissues, we might expect its genetic architecture to be intermediate between traits exclusively enriched for the CNS and non-brain-related traits. This is what we see for body composition-related traits (Fig. 2*D* and *SI Appendix*, Fig. S15*B*), which are partially mediated by the CNS through appetite control and preference for food intake (41), but are also mediated by other tissues (*SI Appendix*, Fig. S15*A*). Here, we focus on the pronounced differences between brain-related and other traits, but we revisit the potential roles of other tissues on trait genetic architectures in *Discussion*.

Importantly, we sought to evaluate whether these patterns were driven by uncontrolled confounding in population-based GWAS of these traits. By comparing results from population-based and family-based association studies, previous studies have suggested that confounders including population stratification, indirect genetic effects, and assortative mating, can aggregate across the genome to bias estimates of heritability and genetic correlation (42–45). However, our analyses focus on GWAS hits, and we expected confounding effects at these strong-signal sites to be minor (45, 46). To check this, we first used a GWAS of birth coordinate as a negative control where we would expect most signals to be driven by confounding (excluding a few that are protective against allergies 47). MAF of GWAS hits for birth coordinate traits have a cumulative distribution that is distinct from any of the traits we considered (*SI Appendix*, Fig. S16). Using paired sibling- and population-based GWAS summary statistics (42), we confirmed that median MAF of significant hits is a stable measure of genetic architecture for well-powered traits. For traits with limited power, the significant hits identified in this study show no MAF-dependent attenuation of significance in sibling-based GWAS (*SI Appendix*, Fig. S17). Furthermore, we found that two urinary biomarkers were classified as brain-related, with GWAS architectures resembling other brain-related traits (*SI Appendix*, section 5 and Fig. S18). Taken together, these analyses suggest that the patterns described here are not driven by uncontrolled confounding. However, because family-based GWAS provide the most reliable estimates of direct genetic effects but remain substantially underpowered, we caution that this possibility cannot be entirely ruled out.

### Brain-Related Traits Retain Distinct Architectures After Accounting for Differences in Study Design.

To ensure a rigorous comparison between brain-related and other traits, we next considered differences in study design that could potentially confound the observed genetic architectures.

**Fig. 3.**
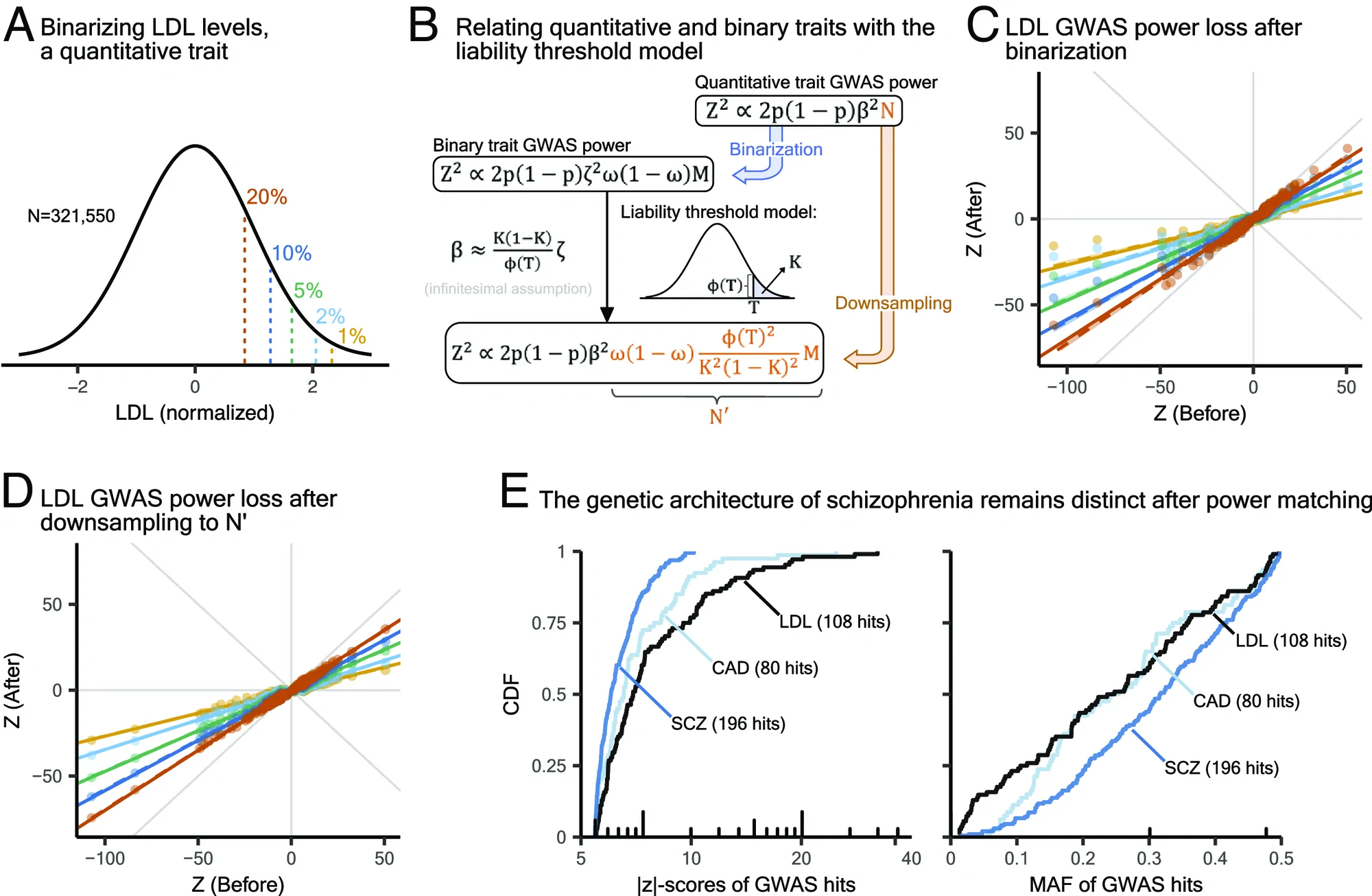
Matching GWAS statistical power between quantitative and binary traits. (*A*) Binarizing LDL with varying prevalence in the upper tail. LDL levels were measured in unrelated white British individuals from the UK Biobank. The original phenotypic distribution was standardized using rank-based inverse normal transformation. (*B*) Derivation of the sample size needed on the liability scale to reach equivalent power of a case–control GWAS. MAF is denoted by *p*. We transferred effect sizes from log odds ratio scale (*ζ*) to continuous scale (*β*) using the liability threshold model. For details, see *SI Appendix*, section 2. (*C*) Deflation of *z*-scores for LDL GWAS hits after binarizing. Different colors reflect the threshold used to binarize traits, as shown in panel (*A*). Solid lines represent predicted slopes, and dashed lines indicate fitted slopes. Gray lines in the background are y=0, x=0, and y=±x. (*D*) Deflation of *z*-scores for LDL GWAS hits after downsampling to matched sample sizes. Color choices, line styles, and background lines are identical to those in panel (*C*). (*E*) Reducing coronary artery disease and LDL GWAS power to match with the current effective sample size of schizophrenia GWAS, $N^{\prime }=192,273$ (*Materials and Methods*).

When using significant hits across studies that differ in trait type (binary versus quantitative), sample size, or disease prevalence, we first demonstrated that formulas originally derived to adjust for GWAS statistical power at the genome-wide scale also apply to significant hits. As a motivating example, we converted LDL levels, a quantitative trait, into binary traits by applying thresholds corresponding to a range of population prevalences (Fig. 3*A* and *SI Appendix*, Fig. S19*A* and *Materials and Methods*). To simplify the exposition, we assume here that population prevalence equals sample prevalence.

Based on the classical liability threshold model (48), an individual’s liability is a quantitative trait that relates to their genotype through the standard additive model, and individuals with liability exceeding a threshold *T* develop the disease. Previous studies have since established that the power to identify a variant in a case–control study with a sample size of *M* individuals, with a proportion *ω* of cases and (1−ω) of controls, is approximately equal to the power to identify that variant in a GWAS of liability with effective sample size

[1] $$\begin{array}{c} N^{\prime }\approx M\omega \left(1-\omega \right)\frac{\phi {\left(T\right)}^{2}}{K^{2}{\left(1-K\right)}^{2}}, \end{array}$$

where *K* is the population prevalence of the disease, and ϕ(·) denotes the standard normal density (11, 49–53); see also Fig. 3*B* and *SI Appendix*, sections 1–3].

Importantly, Eq. **1** relies on the infinitesimal assumption, namely that the effect size of the focal variant is small (*SI Appendix*, section 2). Since genome-wide significant variants have (relatively) large effect sizes, this assumption may not hold for significant hits. To address this concern, we used independent hits from the original quantitative LDL GWAS and evaluated the extent to which their *z*-scores were deflated in case–control GWAS across different binarization thresholds (Fig. 3*C* and *SI Appendix*, Fig. S19*B*). We also performed quantitative LDL GWAS on a subsample whose size was determined by Eq. **1** to match the reduction in power caused by binarizing for each threshold we used (Fig. 3*D*). Both binarizing and downsampling are expected to approximately linearly deflate *z*-scores with

[2] $$\begin{array}{c} Z_{\mathrm{after}}\approx \sqrt{\frac{N^{\prime }}{N}}Z_{\mathrm{before}}, \end{array}$$

where *N* is the sample size of the original quantitative trait GWAS (*SI Appendix*, section 3).

Fig. 3 *C* and *D* share the same set of predicted lines as given by Eq. **2**, which accurately predict the effects of both binarizing and downsampling on significant hits. Variants of large effect deviate from the linear relationships in Fig. 3*C* and *SI Appendix*, Fig. S19*B* but not in Fig. 3*D*, because Eq. **1** relies on the infinitesimal assumption, whereas Eq. **2** does not.

We repeated this analysis with five other medically relevant quantitative traits in the UK Biobank to ensure that these results were not unique to LDL (*SI Appendix*, Figs. S20–S24). Unexpectedly, many traits exhibit asymmetrical power reduction when binarizing in the lower tail compared to the upper tail. For example, BMI shows more deflation than expected when binarizing in the lower tail, and less deflation in the upper tail (*SI Appendix*, Fig. S20). This pattern suggests that the same set of variants have, on average, stronger effects in high-BMI individuals than in low-BMI individuals. One possible explanation for this tail asymmetry is genetic epistasis, in which the magnitude of a variant’s effect varies depending on an individual’s genetic background, resulting in larger effects at one end of the liability distribution compared to the other. Gene–environment interaction is another plausible mechanism and has been proposed to explain why polygenic scores achieve higher predictive accuracy in higher BMI quantiles than in lower ones (54). While intriguing, these deviations from our theory are relatively minor and determining the precise mechanism is beyond the scope of the current study.

Most disease GWAS included in our analyses were meta-analyses of multiple cohorts, in which case the effective sample size cannot be calculated by simply substituting the total sample size (*M*) and overall case fraction (*ω*) into Eq. **1**. Instead, the appropriate approach is to estimate $M\omega (\hat{1}-\omega )$ by summing across cohorts (53). For all binary traits, the estimated $M\omega (\hat{1}-\omega )$ values were indeed smaller than those obtained using the naive approach (*SI Appendix*, Table S2). We then calculated $N^{\prime }$ using Eq. **2** together with the population prevalence values listed in *SI Appendix*, Table S2. Even after matching all three example traits in Fig. 1 to the same $N^{\prime }$, we continue to see differences in the CDFs of |z|-scores and MAF for independent GWAS hits (Fig. 3*E*).

Next, we investigated whether aspects of GWAS study design other than statistical power could confound the observed genetic architectures. Potential confounders include, but are not limited to, ascertainment bias, sample prevalence, measurement context, phenotype heterogeneity, and technical differences across GWAS, including imputation panels, quality control procedures, and residual population stratification. To evaluate the impact of these factors, we compared three schizophrenia GWAS with nested samples of European ancestry. We found highly similar genetic architectures across the three studies (*SI Appendix*, Fig. S25), despite their differences in sample prevalence, imputation panels, and the proportion of cases with treatment-resistant schizophrenia receiving clozapine (*SI Appendix*, Table S3). Additionally, all disease GWAS used in this study have LDSC intercepts close to 1 (*SI Appendix*, Table S1), suggesting adequate control of population stratification.

Taken together, differences in GWAS study design are unlikely to explain the differences in architecture between brain-related and other traits, suggesting that other biological or evolutionary mechanisms are involved.

### Brain-Related Traits Have Large Mutational Target Sizes and Variants Under Strong Selection.

**Fig. 4.**
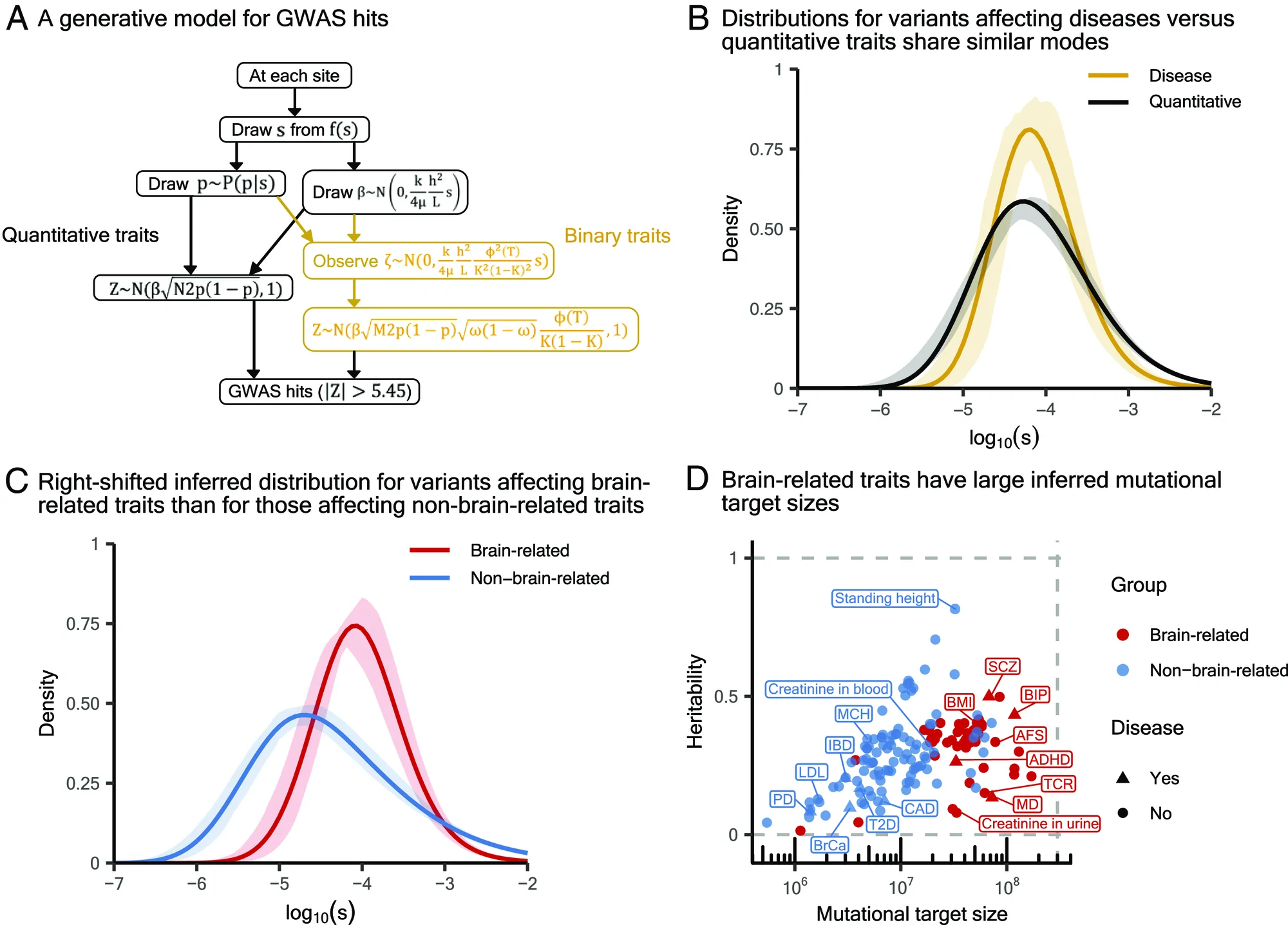
Stronger selection on brain-related variants and larger mutational target sizes for brain-related traits. (*A*) The model that generates MAF, effect size, and observed GWAS *z*-score at each genomic position, adapted from ref. 15 using Eq. **1** to include binary traits. *μ* is the mutation rate, and k≡4μ/E[2q(1−q)s]≈1 depends on f(s) and demography (see SI Appendix, section 1.5 in ref. 15). $h^{2}$ for a binary trait refers to heritability on the liability scale. (*B*) Inferred distributions of selection coefficients estimated from 151 quantitative traits and 13 human complex diseases and disorders, respectively, with 90% confidence envelopes shown. (*C*) Inferred distributions of selection coefficients when trait were instead grouped into 54 brain-related traits and 110 non-brain-related traits, with 90% confidence envelopes shown. (*D*) Median estimates of heritability and mutational target size, inferred jointly with the respective f(s) shown in panel (*C*). The gray lines are drawn at $x=3\times 10^{8}$, and y=0 and 1. MCH: Mean corpuscular hemoglobin.

To formally investigate alternative explanations, we turned to the generative version of the pleiotropic stabilizing selection model developed by Simons et al. (15) (see a summary in *SI Appendix*, Box 1 and the model shown in the black text in Fig. 4*A*). In this framing, mutations affecting the trait arise at a rate that depends on the trait’s mutational target size *L*, and are then assigned a selection coefficient s>0 from a distribution f(s). The frequency of the mutation at the time the GWAS is performed, *p*, is sampled from a distribution that arises from underdominant selection with the selection coefficient *s* and the effects of genetic drift during the demographic history of the population. We assume a demographic model estimated for the British population (55) and a mutation rate of $1.25\times 10^{-8}$ (14). The effect size of the mutation, *β*, also depends on *s*: Specifically, β|s∼N(0,c·s), where *c* is a trait-specific constant that depends on the heritability per site in the target, $h^{2}/L$. The variant is then assigned a z-score sampled from a distribution that depends on its effect size, allele frequency, and GWAS sample size. When this z-score exceeds the genome-wide significance threshold, the variant is considered a GWAS hit. Below, we fit f(s) using a spline function with four knots (see SI Appendix, section 4.8 in ref. 15), thus our full model includes six parameters per trait: four for f(s), as well as $h^{2}/L$, and *L*.

We extended the Simons et al. model to accommodate binary traits using Eq. **1** (see the orange text in Fig. 4*A*). The extended model provides a robust fit to complex diseases and disorders with more than one hundred hits (*SI Appendix*, section 6), support ing the view that common variants underlying complex diseases and disorders are shaped by pleiotropic stabilizing selection (7, 33, 34).

To include traits with fewer hits in our inference, we fitted a model in which the architectures of a chosen set of traits are parameterized by a shared f(s) distribution and two trait-specific parameters, $h^{2}/L$ and *L*. We therefore expanded our analysis to include all 151 quantitative traits and 13 diseases and disorders, each with more than 20 approximately independent hits. When traits were divided into quantitative and binary traits, the inferred distributions of selection coefficients have similar modes (Fig. 4*B*).

In contrast, the inferred trait-specific f(s) distributions among traits with more than a hundred GWAS hits differ markedly between brain-related and other complex traits (*SI Appendix*, Fig. S26). We therefore performed inference on traits grouped into these categories. Our results indicate that mutations affecting brain-related traits are subject to stronger selection than those affecting other traits (Fig. 4*C*). Moreover, the shared f(s) for brain-related traits is narrower than that for non–brain-related traits, which suggests a narrower distribution of effect sizes for brain-related traits, consistent with previous studies (see Introduction and refs. 31 and 32).

Our inference also indicates that brain-related traits tend to have substantially greater mutational target sizes than other complex traits (Fig. 4*D* and *SI Appendix*, Fig. S27), echoing previous studies that found brain-related traits to be more highly polygenic than other traits (see Introduction and refs. 25 and 28–30). To contextualize these estimates, approximately 10% of the genome has been estimated to be constrained by selection and is therefore considered putatively functional (56–58). For brain-related traits, our median inferred mutational target size is approximately 1.32% of the genome, compared with 0.27% for non-brain-related traits.

Unlike the distributions of selection coefficients and mutational target sizes, the distributions of estimated heritability do not differ significantly between brain-related and other traits (Fig. 4*D* and *SI Appendix*, Fig. S27). In turn, estimates of $h^{2}/L$, which is the key scaling parameter governing GWAS power and relating trait architectures within each category (15), are markedly lower for brain-related traits.

To validate our heritability estimates, we compared them with the recent whole-genome sequencing–based estimates ($h_{\mathrm{WGS}}^{2}$) (59). For the set of overlapping quantitative traits, our $h^{2}$ estimates derived from brain-related and non-brain-related f(s) distributions show greater concordance with $h_{\mathrm{WGS}}^{2}$ than estimates obtained under a single quantitative f(s) model, suggesting that grouping traits by CNS status improves model performance (*SI Appendix*, Fig. S28).

### Strong Selection and Large Mutational Target Size Explain the Distinct Architecture of Brain-Related Traits.

We next wanted to see if the evolutionary parameters we inferred could explain the genetic architectures of brain-related traits. In particular, we used simulations to see the impact of these parameters on the MAF and |z|-score distribution of GWAS hits, which set apart the genetic architectures of brain-related and other traits.

**Fig. 5.**
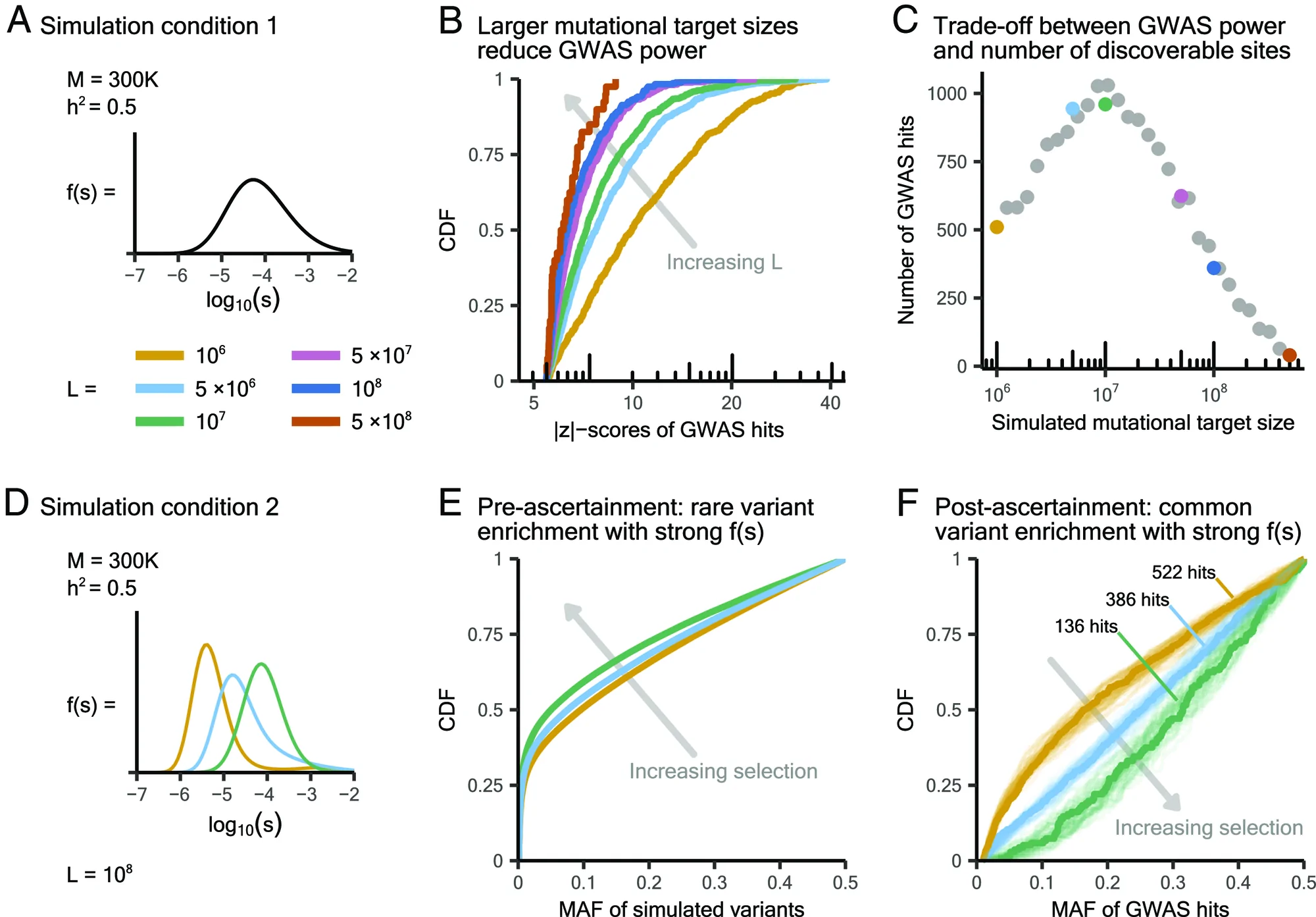
The differences in evolutionary parameters explain the differences in architecture between brain-related and other complex traits. (*A*) Simulations of traits with varying mutational target sizes. We fixed f(s) to the shared distribution inferred from 151 quantitative traits as shown in Fig. 4*B*. Colors in panels (*B* and *C*) correspond to the simulation settings shown in (*A*). (*B*) Larger mutational target sizes lead to narrower distributions of GWAS hit |z|-scores. (*C*) Nonmonotone relationship between mutational target size and number of GWAS hits. We ran additional simulations at finer increments of *L* and plotted them as gray dots. (*D*) Simulations of traits with varying distributions of selection coefficients. Colors in panels (*E* and *F*) correspond to the simulation settings shown in (*D*). (*E*) In the absence of ascertainment for GWAS hits, stronger selection leads to variants of lower MAF. (*F*) After ascertainment for GWAS hits (MAF >1% and |Z|>5.45), stronger selection leads to GWAS hits of higher MAF. We ran 50 simulations for each f(s) distribution, highlighted the median CDF curve of the MAF of simulated hits, and labeled the corresponding number of hits.

We first examined the impact of mutational target size. To this end, we fixed f(s) to the distribution estimated from 151 quantitative traits, held the sample size at 300 K and the trait heritability at 0.5, and varied the mutational target size (Fig. 5*A*). We found that larger values of *L* lead to steeper CDF curves for GWAS hit |z|-scores (Fig. 5*B*). This occurs because GWAS power is linearly proportional to the scaling parameter $h^{2}/L$ (Fig. 4*A*). The number of GWAS hits, on the other hand, scales nonlinearly with *L* (Fig. 5*C*). While increasing *L* makes more genomic sites relevant to the trait (15), the power to discover each individual site declines. These opposing effects interact to produce the observed nonlinear trend. In turn, the distribution of MAFs of GWAS hits is fairly insensitive to changes in mutational target size, indicating that differences in mutational target size cannot explain the higher MAFs that we observed for GWAS hits of brain-related traits (*SI Appendix*, Fig. S29).

Critically, we found that stronger selection leaves a counterintuitive signature on GWAS genetic architecture, enriching significant hits for higher MAF. To isolate the effect of selection, we held the mutational target size at $10^{8}$ bp and varied the distribution of selection coefficients (Fig. 5*D*). As expected, in the absence of GWAS ascertainment, variants simulated under stronger selection are generally rarer than those from weaker selection (Fig. 5*E*). However, this relationship is reversed after GWAS ascertainment: After restricting to common variants and selecting hits that surpass the genome-wide significance threshold, stronger selection becomes associated with higher MAF among significant GWAS hits (Fig. 5*F*). When trait-affecting variants are under stronger selection, only small effect variants can reach sufficiently high frequencies to be included in GWAS. Due to this ascertainment bias, an overrepresentation of small effect variants in turn leads to an enrichment of variants with higher MAF (*SI Appendix*, Fig. S30*B*). Stronger selection also contributes to steeper |z|-score distributions and fewer hits (*SI Appendix*, Fig. S30 *C* and *D*). We note that, for a fixed f(s), stronger selection implies larger effect sizes under the stabilizing selection model. However, this relationship does not necessarily extend across different f(s) distributions, because the parameter *k* in the model depends on f(s) (Fig. 4*A*). Taken together, these results demonstrate that varying f(s) produces characteristic changes in multiple features of GWAS genetic architecture, including the MAF distribution, |z|-score distribution, and the number of significant hits, thus providing information that distinguishes f(s) from the mutational target size *L*.

### Rare Variant Burden Tests Suggest Strong Selection on Brain-Relevant Genes.

Although simulations show that stronger selection on genetic variation can generate GWAS hits of higher MAF and lower |z|-scores—patterns consistent with what we observed for brain-related traits—these same GWAS hits are also the signals we used to infer f(s). To seek orthogonal evidence in support of stronger selective constraint on genetic variation influencing brain-related traits, we next turned to a genic perspective and asked: Are genes relevant to brain-related traits also under stronger selection compared to genes relevant to other traits?

To that end, we leveraged previously reported estimates of the fitness effects of loss-of-function (LoF) variants, denoted $s_{\mathrm{het}}$ (60). These estimates quantify selection against heterozygotes and serve as a metric of gene-level constraint. Moreover, from burden tests of these LoF variants, we obtained gene-to-trait effect estimates, $\hat{\gamma }$, which we then used to derive unbiased estimates of the squared effects, $\hat{\gamma^{2}}$ (*Materials and Methods*). One caveat to note is that our analysis did not include burden tests on binary traits, primarily due to limited data and the fact that statistical power is biased toward detecting risk-increasing effects in the rare variant regime.

Under a model of pleiotropic stabilizing selection, in which selection against LoF variants is mediated through their effects on traits, Spence et al. recently showed that on average $\hat{\gamma^{2}}$ increases with increasing $s_{\mathrm{het}}$ across a set of independent quantitative traits (40). In addition, variation in brain expression was the most predictive gene expression feature for $s_{\mathrm{het}}$ among all tissues examined (60). Our multiple regression analyses also showed that whether or not a gene is expressed in the “frontal cortex” is among the strongest predictors of baseline $s_{\mathrm{het}}$ estimates obtained from a model trained without any gene features (*SI Appendix*, Fig. S31). Together, these observations led to the hypothesis that brain-relevant genes are more constrained than other genes and that in constrained genes, the fitness effects of LoF variants should correlate more strongly with their effect on brain-related traits than on non-brain-related traits.

**Fig. 6.**
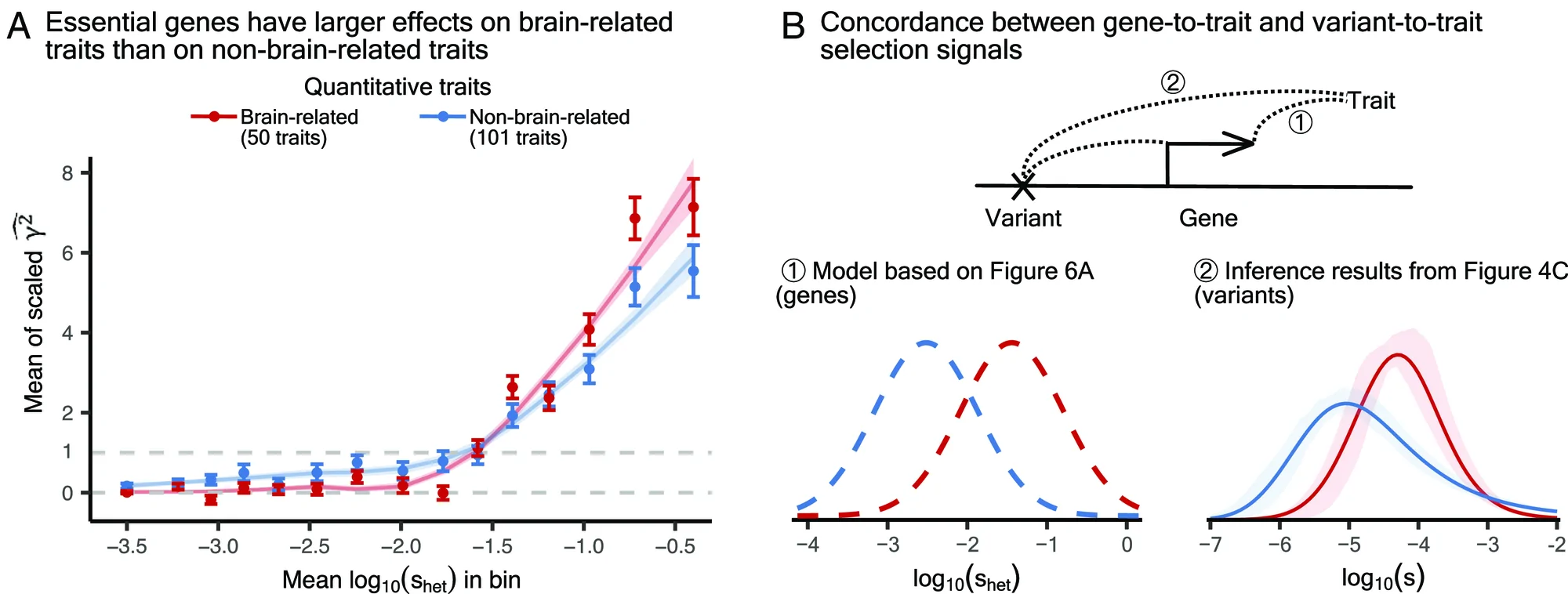
Burden tests corroborate stronger selection on genetic variation in the CNS compared to other tissues. (*A*) Mean squared genic effects on brain-related versus other quantitative traits, with genes grouped into 15 quantiles of $s_{\mathrm{het}}$ estimates (60). The estimated $\hat{\gamma^{2}}$ values are scaled so that the mean across all gene-trait pairs in the tenth bin equals one. All estimates are shown with a 95% CI computed from the SE. We bootstrapped genes at the trait level and showed the mean and 95% CIs of the fitted LOESS curves. (*B*) Comparing selection signals from gene-to-trait and variant-to-trait associations. ① Brain-relevant genes are under stronger selection than genes relevant to other traits. The two curves are hypothetical and are intended to illustrate the relationship between their modes. ② Brain-relevant variants are under stronger selection than variants relevant to other traits.

Binning genes by $s_{\mathrm{het}}$, we indeed found that genes that are more constrained have larger relative squared effect sizes, $\hat{\gamma^{2}}$, on brain-related than on other quantitative traits, and vice versa for less constrained genes (Fig. 6*A*). If these two trait categories had systematic differences in heritability or other factors, one curve would be consistently higher across the range. Instead, the two curves switch order, ruling out this possibility and supporting the hypothesis that effects on brain-related traits more strongly correlate with fitness consequences. In addition, we pruned the set of quantitative traits to 32 approximately uncorrelated traits, comprising 11 brain-related traits and 21 non-brain-related traits (*Materials and Methods* and *SI Appendix*, Fig. S32). Although the signal is noisier, we observed the same S-shaped trend (*SI Appendix*, Fig. S33).

Fig. 6*A* suggests a model in which brain-relevant genes are, on average, under stronger selection than genes relevant to other traits (① of Fig. 6*B*). If we assume that variants in the CNS and other tissues have similar effects on gene activity, this could help explain why selection on brain-relevant variants is stronger than on other traits (Fig. 4*C*, plotted again in ② of Fig. 6*B*). Therefore, the strong selection signals we found for the brain-related traits are concordant at both the variant and gene levels.

### From Evolutionary Inference to Predicting GWAS Discoveries.

**Fig. 7.**
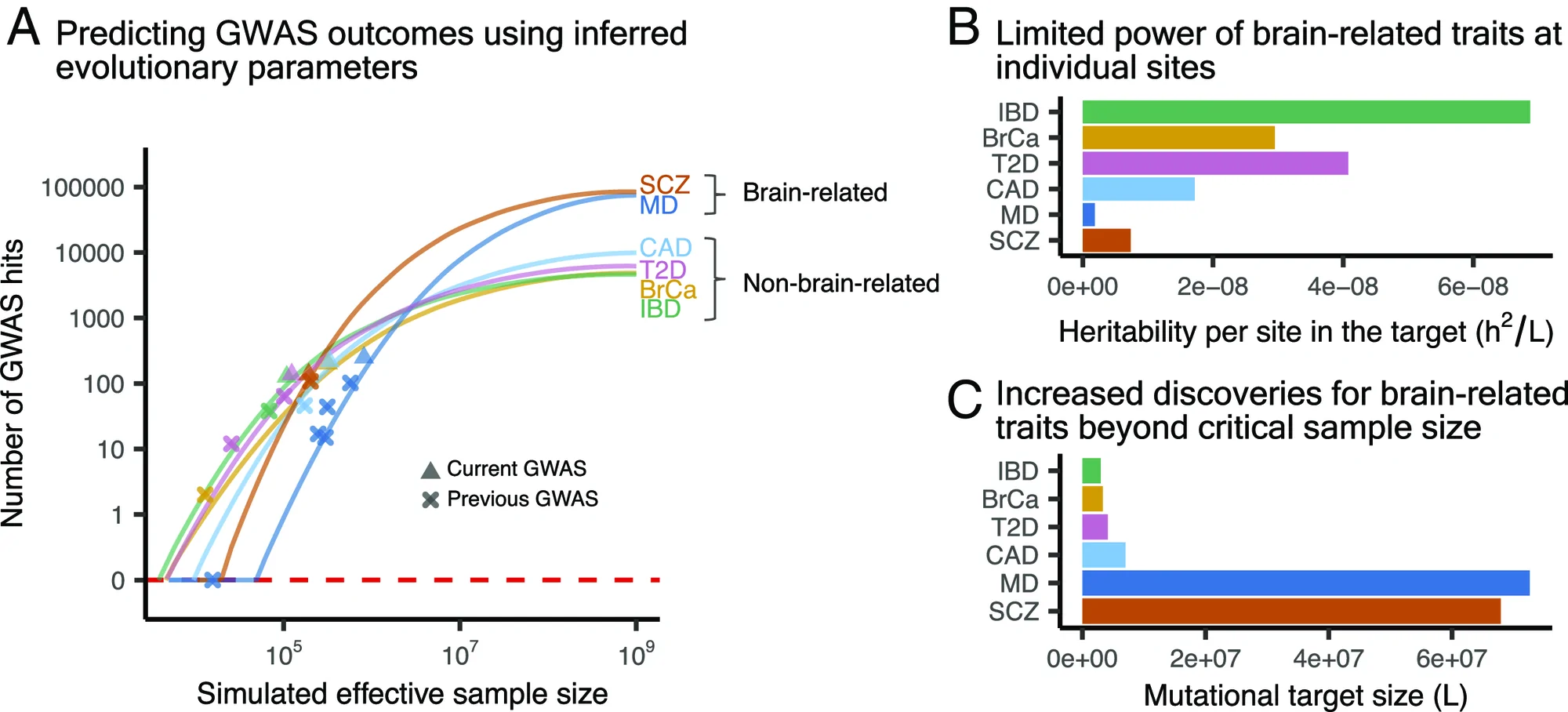
Explaining past and predicting future disease GWAS discoveries. (*A*) Expected number of hits in disease GWAS as a function of sample size, based on simulations using the inferred f(s) for brain-related and other traits (Fig. 4*C*), and disease-specific estimates of $h^{2}$ and *L*. We evaluated a range of sample sizes across 35 grid points and plotted the curves using the median values across 10 simulations at each point. Our inference was based on the current GWAS (*SI Appendix*, Table S1). For reference, we overlaid the reported number of hits and sample sizes from selected earlier GWAS [IBD (61); BrCa (62); T2D (63, 64); CAD (65); MD (66–70); and SCZ (71)]. (*B*) Inferred estimates of $h^{2}/L$ used in the simulations. (*C*) Inferred estimates of *L* used in the simulations.

Our inferred evolutionary parameters can explain past GWAS findings and predict future results. Focusing on the six complex diseases and disorders with more than a hundred GWAS hits, Fig. 7*A* shows the number of independent GWAS hits in simulations as a function of the effective study size (Eq. **1**). In particular, our findings for major depression recapitulate the historical trajectory: Early studies showed a prolonged period with few or no significant findings (66, 72, 73), followed by a recent sharp increase in the number of detected loci (67–70, 74). Our inference explains these historical trends. To a good approximation, variants reach genome-wide significance when their contribution to phenotypic variance exceeds a threshold inversely proportional to the study’s sample size. Given the low heritability per site for major depression (Fig. 7*B*), larger sample sizes are required to surpass this threshold, after which the number of discoveries sharply rises due to its large mutational target size (Fig. 7*C*). More generally, we expect the future numbers of GWAS hits for brain-related traits to increase more rapidly than those for other traits, ultimately reaching higher levels of saturation.

## Discussion

What are the processes that shape the genetic architecture of complex traits? Do certain kinds of traits share similar architectures, and if so, what causes them to differ from other kinds of traits?

We found that brain-related quantitative traits and complex disorders share distinct genetic architectures, in that their GWAS hits have modest significance, small effect sizes, and higher MAF. These features can be explained by genetic variation affecting brain-related traits having larger mutational target sizes and experiencing markedly stronger selection than those affecting other traits.

To support our inference that brain-related traits have larger mutational target sizes, we examined how genes expressed in the brain differ from those expressed in other tissues. Although brain tissues do not express more genes (*SI Appendix*, Fig. S34*A*), brain-specific genes are, on average, longer (75–78) (*SI Appendix*, Fig. S34*B*), contain longer introns (78), produce more isoforms (79), and have longer intergenic distances to neighboring genes (80, 81) (*SI Appendix*, Fig. S34*C*). Intriguingly, the pattern of intergenic length-dependent enrichment in gene expression is observed only in neurons, oligodendrocytes, and astrocytes, but not in microglia cells (81). Given the central role of microglia in neurological diseases such as Alzheimer’s disease and Parkinson’s disease (82), this distinction may help explain why we observed differences in genetic architectures between neurological diseases and psychiatric disorders.

The LoF burden test analyses corroborate that genetic variation affecting the CNS is under stronger selection (Fig. 6). Further supporting this conclusion, among constrained genes depleted of missense and LoF variants, 30% are highly expressed in the brain, compared with only 14% in lymphocytes, which rank second across tissues (83). Our main analyses were based on common variants, which are largely regulatory (84). To directly assess evolutionary constraint in regulatory regions, multiple sequence alignment of primate and mammalian genomes revealed that open chromatin in neural tissues has the highest phyloP scores, and that sites without a single substitution across all species are enriched for regulatory elements in the brain (58, 85). A recent study modeling heart and brain chromatin accessibility also reported that rare variants have the largest regulatory effects relative to common variants in fetal neurons, suggesting that fetal neurons may disproportionately shape evolutionary pressures (86).

Together, these findings suggest that both coding and regulatory variation affecting the CNS is subject to strong evolutionary constraint. If selection on variants affecting brain-related traits is stronger than for other traits, then we would expect a greater proportion of their heritability to come from rare, noncoding variants. In line with these expectations, recent studies using whole-genome sequencing data have found that brain-related traits exhibit relatively strong heritability enrichment from rare variants and the highest enrichment from noncoding variants across all traits examined (59).

Importantly, our findings should not be interpreted as evidence that the brain-related traits we examined, especially the behavioral-cognitive traits, are themselves under stronger selection. Under the pleiotropic stabilizing selection model that we fitted, most of the selection on genetic variation for a focal trait arises from other traits in the high-dimensional trait space. For example, selection may act primarily on core aspects of brain development and function, and variants affecting these processes may also influence the brain-related traits we focused on. As a result of this feature of the model, we cannot determine the specific traits on which the inferred strong selection acts. The brain-related traits we analyzed can therefore be viewed as proxies, tagging the variants and genes involved in essential biological processes. Since brain-related traits are highly correlated (87), they could in principle collapse onto a single underlying trait, thereby undermining the assumption of a high-dimensional trait space. To test this assumption, we used simulations assuming no pleiotropic variant effects to show that the observed MAF distributions of GWAS hits for brain-related traits cannot be recovered (*Materials and Methods* and *SI Appendix*, Fig. S35).

Why would selection on genetic variation be stronger for brain-related traits compared to traits mediated by other tissues? Brain tissues are distinct in that expression quantitative trait loci (eQTL) effects show high levels of sharing within the brain, yet remain largely unshared with other tissues (88). This suggests that variants regulating gene expression in the brain may be largely brain-specific. Moreover, the brain appears to be particularly vulnerable to broad disruptions in transcriptional regulation: Mutations affecting histone modification, chromatin remodeling, cohesin-mediated chromatin looping, and transcription elongation predominantly result in developmental delay and intellectual disability (89), and systematic disruption of long-gene expression can cause autism (75) and Rett syndrome (76). This pattern is particularly well documented for chromatin-modifying disorders, 83% of which involve intellectual disability (90). These findings suggest that the brain has unique transcriptional regulatory machinery, that perturbations to transcription are more likely to affect the brain, and that disruption of gene expression in the brain carries disproportionately severe fitness consequences relative to other tissues.

With most common variants being regulatory, we can envision the strong selection happening in three ways, when comparing CNS to other tissues: i) variants could typically have greater effects on the activity (e.g., expression) of genes; ii) changes in the activity of genes could typically have greater effects on traits; and iii) changes in the activity of genes typically affect more selected traits. The genic analyses we presented (Fig. 6) suggest that changes to gene expression involve greater fitness effects in the CNS than in other tissues, which in turn supports the latter two models (without excluding the first).

Our analysis also suggests that traits that are mediated by the same tissues tend to have similar architectures and thus similar evolutionary parameters. Brain-related traits appear to occupy a “corner” in the architecture space, which is why their genetic architectures are more clearly set apart from the architecture of traits mediated by other tissues, but we also see a clustering of architectures for cardiovascular and skeletal muscle traits, for example (*SI Appendix*, Figs. S13 and S14). The omnigenic model (91, 92) suggests why trait architectures might cluster by tissues. If many of the regulatory variants that affect gene activity in given cell types contribute to heritable variation in traits that are mediated by these cell types, then we would expect these traits to share similar mutational target sizes and distributions of selection effects; the sharing of effect size distributions (scaled appropriately) follows from the shared distribution of selection effects under the pleiotropic stabilizing selection model (14). Nonetheless, our inference suggests that there is substantial variation in mutational target sizes and distribution of selection effects among brain-related traits (*SI Appendix*, Figs. S27 and S26). This too is to be expected: Even setting aside the coarseness in associating traits with tissues and the uncertainty in our inferences, traits that are mediated by the same tissue are expected to have different numbers of “core genes” and to be differentially affected by different cellular circuits.

The case of body composition-related traits is especially interesting from this point of view. As noted above, these traits are mediated by a mixture of tissues, including the CNS (*SI Appendix*, Fig. S15). Their architectures and inferred evolutionary parameters appear to fall between those of brain-related traits mediated primarily by the CNS and those of non-brain-related traits (Fig. 4*D* and *SI Appendix*, Fig. S27). More generally, this suggests that the architecture of common variation underlying complex traits can be approximated as a superposition of architectures associated with tissues, with some trait-specific effects in each tissue.

## Materials and Methods

### Identifying Approximately Independent Hits Using GCTA-COJO.

From GWAS summary statistics, we applied GCTA-COJO (38) to identify conditionally independent hits and re-estimated variant effect sizes $\hat{\beta }$ on each trait. We used parameters --cojo-p 5e-8 and --cojo-slct, along with a LD reference panel consisting of genotypes from a subset of 10,000 White British individuals from the UK Biobank. We removed hits with a minor allele frequency (MAF) below 1%, those located in the HLA region, and those with INFO scores below 0.8.

### Data on 151 Quantitative Traits.

We downloaded GWAS summary statistics for 305 quantitative phenotypes (Dataset S1; same trait list as in ref. 40) from the Neale Lab (http://www.nealelab.is/uk-biobank, version 3). Specifically, these results were based on 361,194 individuals of White British ancestry in the UK Biobank. All the phenotypes were rank-based inverse normal transformed. Age, age^2^, inferred sex, age×inferred sex, age^2^×inferred sex, and principal components 1 to 20 were included as covariates. Estimates of SNP heritability for the 305 quantitative traits were downloaded from the Neale Lab. We followed the same procedure as described in the subsection for identifying approximately independent hits. For this study, we focused on 151 quantitative traits, each with at least 20 significant hits and a SNP $h^{2}$ greater than 5% (Dataset S1).

### Data on 13 Complex Diseases.

**GWAS summary statistics.** Since the UK Biobank cohort has been found to be healthier than the general population (93), we turned to publicly available latest GWAS summary statistics from case–control studies. We restricted our analyses to studies on cohorts of European ancestry, as the demographic model in ref. 15 was specifically derived for the European population. For several diseases, the complete results require additional approval from 23andMe. We thus used only the publicly available version, excluding the 23andMe cohort. We followed the same procedure as described in the subsection for identifying approximately independent hits. Using the same cut-off of more than 20 genome-wide independent hits, we curated a list of 13 complex diseases and disorders (*SI Appendix*, Table S1).

### Using Stratified LD Score Regression (S-LDSC) to Define Brain-Related Traits.

GWAS summary statistics on all 164 traits were processed by the “munge_sumstats.py” script provided by LDSC developers (https://github.com/bulik/ldsc, v1.0.1) (94). LD scores were computed based on European ancestry participants in the 1000 Genomes Phase 3 dataset (95). To identify key cell types for each trait, we conducted cell type–specific analyses with S-LDSC (26). Finucane et al. categorized 220 distinct cell types into 10 categories: adrenal/pancreas, cardiovascular, central nervous system (CNS), connective/bone, gastrointestinal, immune/hematopoietic, kidney, liver, other, and skeletal muscle. Each cell-type-specific annotation was then added to the baseline LD model (v1.2) to evaluate heritability enrichment. The resulting regression coefficient z-scores were converted to one-sided *P*-values, shown in Fig. 2*B* and *SI Appendix*, Figs. S7–S9. To account for multiple testing, we applied a Bonferroni correction, setting the significance threshold to a *P*-value of 0.05/(220×164).

In Fig. 2*D* and *SI Appendix*, Figs. S13 and S14, the *P*-values for each cell-type category are obtained from meta-analyses using the aggregated Cauchy association test (ACAT), which is robust to the correlations among the constituent *P*-values (39). Specifically, within each category, *P*-values were transformed into Cauchy variables and aggregated through $T=\sum_{i=1}^{k}\frac{1}{k}\tan\left\{\left(0.5-p_{i}\right)\pi \right\}$. The resulting *P*-value can be obtained from the Cauchy distribution with 0.5−{arctan(T)}/π.

From the set of 164 complex traits, 50 quantitative traits and 4 complex disorders with meta-analysis *P*-values for CNS enrichment passing the Bonferroni threshold of 0.05/(10×164) are classified as brain-related traits. The remaining 101 quantitative traits and 9 complex diseases are classified as non-brain-related traits (Dataset S1).

### Thresholding and Downsampling Quantitative Traits.

We used UK Biobank individual-level data for the analyses described in this section. All data were deidentified prior to our access and analysis. For a given quantitative trait, we obtained recorded trait values from approximately 360 K unrelated White British individuals in the UK Biobank. We first conducted GWAS on rank-based inverse normal transformed phenotypes using PLINK 2.0 (https://www.cog-genomics.org/plink/2.0/) (96), with parameters --glm hide-covar and --covar-variance-standardize. Age, age^2^, sex, age×sex, array, center, and principal components 1 to 20 were included as covariates in the regression model. We then identified conditionally independent hits using GCTA-COJO as described above.

For the thresholding analysis, we dichotomized the continuous trait into ten binary traits (cases = 2 and controls = 1 following PLINK format), representing a disease prevalence of 1%, 2%, 5%, 10%, and 20% on either the lower or the upper tail of the phenotypic distribution. To reduce computational burden, we ran case–control GWAS only on the list of previously identified hits with flag --extract and modified parameter --glm firth-fallback hide-covar.

For the downsampling analysis, we drew five subsamples, each with sample sizes calculated to match with the prevalence rates of 20%, 10%, 5%, 2%, and 1% used to create previous binary traits (*SI Appendix*, section 3). Smaller subsets were ensured to nest within larger ones. Phenotypes in each subset were again rank-based inverse normal transformed to run GWAS using the same variant list filter.

Last, z-scores from each of the ten thresholded or five downsampled GWAS were linearly regressed on z-scores from the original GWAS.

These procedures were repeated for six medically relevant traits, as they are commonly used in clinical settings to declare disease status based on specific cutoffs. These traits include LDL, BMI, hemoglobin A1c (HbA1c), diastolic blood pressure, systolic blood pressure, and heel bone mineral density T-score. The corresponding UK Biobank data fields are 30,780, 21,001, 30,750, 4,079, 4,080, and 78, respectively.

### Estimating *N*^′^ for Binary Traits and Rescaling GWAS Hit Z-Scores.

For a given complex disease or disorder, we defined in Eq. **1** that $N^{\prime }\approx M\omega \left(1-\omega \right)\frac{\phi {\left(T\right)}^{2}}{K^{2}{\left(1-K\right)}^{2}}$ is the effective sample size needed on the underlying liability scale to reach equivalent power as of the original case–control study. When the case–control GWAS is a meta-analysis, Grotzinger et al. showed that Mω(1−ω) should be estimated by summing the cohort-specific values of Mω(1−ω) across all constituent cohorts (53). Let $p_{i}$ be the MAF of SNP *i* and $\hat{\zeta_{i}}$ be its estimated effect size on the log odds ratio scale. Given that $\hat{\zeta_{i}}∼N\left(\zeta_{i},\frac{1}{2Mp_{i}\left(1-p_{i}\right)\omega \left(1-\omega \right)}\right)$ (*SI Appendix*, section 1), we estimated $M\omega (\hat{1}-\omega )$ with the median value of $\frac{1}{2p_{i}\left(1-p_{i}\right)SE^{2}\left(\zeta_{i}\right)}$ across all SNPs in the summary statistics file. Although derived using slightly different reasoning, our approach is essentially equivalent to the procedure described in ref. 53 in settings when cohort-specific information is unavailable (*SI Appendix*, section 6).

The remaining term, $\frac{\phi {\left(T\right)}^{2}}{K^{2}{\left(1-K\right)}^{2}}$, requires estimating population prevalence of the disease, *K*. Estimates of *K* shown in *SI Appendix*, Table S2 were taken from the GWAS source paper when available. Otherwise, we used prevalence estimates in the European population. Assuming that disease liability follows a standard normal distribution, we estimated the liability threshold *T* from *K*, and then computed ϕ(T) as the standard normal density evaluated at this threshold.

When standardizing two traits to have comparable sample sizes, regardless of whether the trait is quantitative with reported sample size *N* or binary with estimated sample size $N^{\prime }$, we simply scale the GWAS hit z-scores of the trait with larger sample size with $\sqrt{\frac{N_{\mathrm{small}}}{N_{\mathrm{big}}}}$.

### Inferring *f*(*s*), *h*^2^, and *L*; and Simulating GWAS Hits.

We used the scripts developed by Simons et al. for analyzing quantitative traits (15). The complete inference framework is described in detail in their *SI Appendix*. Here, we outline the relevant key concepts. With the significant GWAS hits for a given set of traits, we used a maximum likelihood approach to jointly infer a shared f(s) across traits, along with trait-specific parameters, $h^{2}$ and *L*. The log likelihood of observing a GWAS hit *i* is

$${\mathrm{LL}}_{\text{hit}\;i}=\;\log\left(\frac{P\left(Z_{i},p_{i}|h^{2},L,f\left(s\right)\right)}{P\left(\text{hit}|h^{2},L,f\left(s\right)\right)}\right).$$

By the chain rule in probability, the numerator is $P\left(Z_{i}|p_{i},h^{2},L,s\right)P\left(p_{i}|s\right)$. The site frequency spectra, $P(p_{i}|s)$, are estimated with forward simulations assuming a European demographic model. The z-scores then follow $N(0,D\cdot 2p_{i}\left(1-p_{i}\right)N\frac{h^{2}}{L}s+1)$ up to a constant term *D*. The denominator accounts for GWAS ascertainment and is a double integral of the numerator for |z|>5.45 and p>1%. Details can be found in SI Appendix, section 3.3 of ref. 15.

For a given f(s) distribution, we first find each trait *j*’s maximizing $\hat{h^{2}}$ and $\hat{L}$ for

$${\mathrm{LL}}_{\text{trait}\;j}=\left(\sum_{i}{\mathrm{LL}}_{\text{hit}\;i}\right)-0.05\left(\frac{\hat{L}}{3\cdot 10^{8}}+\frac{\hat{h^{2}}}{1}\right),$$

where the minus term penalizes estimates beyond reasonable thresholds. The shared f(s) across traits then maximizes $\left(\sum_{j}{\mathrm{LL}}_{\text{trait}j}\right)$. CIs are obtained by bootstrapping and resampling genomic blocks. Details can be found in SI Appendix, sections 2.8, 3.5, and 3.7–3.9 of ref. 15.

To extend the inference framework to accommodate binary traits, we simply transferred effect sizes from log odds ratio scale to the liability scale (*SI Appendix*, section 2). For binary traits, $h^{2}$ denotes heritability on the liability scale, and *N* represents the corresponding effective sample size on that scale, $N^{\prime }$.

We further adopted the scripts provided by Simons et al. (15) to simulate GWAS hits of a trait, with input parameters f(s), $h^{2}$, and *L*. Briefly, we simulated *L* variants with MAF and z-scores drawn from $P(p_{i}|s)$ and $P(Z_{i}|p_{i},h^{2},L,s)$ and outputted the variants with $p_{i}>1\%$ and |Z|>5.45. Details can be found in SI Appendix, section 5.1 of ref. 15.

To modify the Simons model into a one-dimensional trait space model, i.e., assuming that variants do not have pleiotropic effects, we followed results in Simons et al. (14). Contrary to a high-dimensional trait space in which $\beta ∼N(0,D\cdot \frac{h^{2}}{L}s)$, in a one-dimensional trait space *β* equals $\sqrt{D\cdot \frac{h^{2}}{L}s}$. It follows that the z-scores for a given variant should instead be drawn from $N(\sqrt{D\cdot 2p_{i}\left(1-p_{i}\right)N\frac{h^{2}}{L}s},1)$.

### LoF Burden Tests on 151 Quantitative Traits.

Summary statistics for LoF burden tests on 112 quantitative traits were downloaded from Milind et al. (97). For the remaining 39 quantitative traits, we followed the scripts they provided and conducted gene-based burden tests with REGENIE (98). All UK Biobank individual-level data were deidentified prior to our access and analysis. A whole genome regression model is fit in the first step to make Leave One Chromosome Out (LOCO) phenotypic predictions. We filtered variants for MAF > 1%, missingness < 10%, Hardy–Weinberg equilibrium test *P*-value $<10^{-15}$ and applied linkage disequilibrium pruning with 1,000 variant windows, 100 variant shifts, and $r^{2}<0.9$. In the second step of REGENIE, LoF burden tests were performed with LOCO predictions included as an offset term. We filtered LoF variants to have MAF < 1% and misannotation probability (estimated in ref. 60) <10%. At both stages, we applied rank-based inverse normal transformation to all phenotypes and included age, age^2^, sex, age×sex, genotyping PCs 1 to 15, rare variant PCs 1 to 15, and WES batch as covariates. With the estimated gene-to-trait burden effects, $\hat{\gamma }$, ${\hat{\gamma }}^{2}-\mathrm{SE}{\left(\hat{\gamma }\right)}^{2}$ is an unbiased estimator of mean squared genic effects $\gamma^{2}$ (40, 97).

### Distinct Subsets of (Approximately Uncorrelated) Brain-Related and Non-Brain-Related Traits.

To reduce redundancy in the trait list, we pruned the set of 151 quantitative traits to 32 approximately uncorrelated traits as shown in *SI Appendix*, Fig. S32. Specifically, traits were sorted in decreasing order of meta-analysis *P*-values for CNS enrichment. Starting with the trait that had the strongest enrichment for CNS, time to complete round, we iteratively added traits whose pairwise genetic correlations with all previously selected traits did not exceed 0.5. Genetic correlation estimates were obtained from the Neale Lab. We excluded 27 biomarker traits that did not have genetic correlation estimates. In the end, we selected 32 approximately uncorrelated traits, of which 11 are CNS traits (Dataset S1).

## Supplementary Material

Appendix 01 (PDF)

Dataset S01 (XLSX)

## Data Availability

Codes used for this article are available at https://github.com/huishengz/cns-selection. Data and results are available at https://doi.org/10.5281/zenodo.19154957. To access UK Biobank data, eligible researchers can apply at https://www.ukbiobank.ac.uk/use-our-data/apply-for-access/ (99).
