# Supplementary material for: Genetic architectures of brain-related traits are shaped by strong selective constraints
Source: Proc Natl Acad Sci U S A. 2026 Sep 1;123(36):e2609814123. doi: 10.1073/pnas.2609814123 (PMC13552918; doi:10.1073/pnas.2609814123)
Supplement: Supplementary file 1 [file pnas.2609814123.sapp.pdf]

Supporting Information for

Genetic architectures of brain-related traits are shaped by strong selective constraints

Huisheng Zhu, Yuval B. Simons, Jeffrey P. Spence, Guy Sella and Jonathan K. Pritchard

Corresponding Authors: Huisheng Zhu, Guy Sella and Jonathan K. Pritchard.
E-mail: huisheng@stanford.edu, gs2747@columbia.edu, pritch@stanford.edu

This PDF file includes:

Box 1
Supporting text
Figs. S1 to S35
Tables S1 to S4
SI References

Table of Contents

Box 1

The Simons *et al.* model of pleiotropic stabilizing selection 4

Supplementary Information Text

1. GWAS power. 5
2. Relating effect sizes. 5
3. Binarizing and downsampling a quantitative trait. 6
4. Insights from the formula for estimating N'. 6
5. Urinary biomarkers as an additional check against confounding. 6
6. Evaluating model fit of the extended Simons *et al.* model. 6

Supplementary Figures

Figure S1: Relating effect sizes with the liability threshold model. 9
Figure S2: Reduction in GWAS power after binarizing a quantitative trait. 10
Figure S3: A larger sample size is needed on the liability scale when the disease is rarer. 11
Figure S4: Trait-specific $f(s)$ for six complex diseases and disorders. 12
Figure S5: QQ plots of residual p-values for six complex diseases and disorders. 13
Figure S6: Density plots of GWAS hits for the three example traits. 14
Figure S7: S-LDSC functional enrichment on 151 quantitative traits (first half). 15
Figure S8: S-LDSC functional enrichment on 151 quantitative traits (second half). 16
Figure S9: S-LDSC functional enrichment on 13 human complex diseases and disorders. 17
Figure S10: Density plots of GWAS hits for all the complex traits included in this study. 18
Figure S11: Contrasting neurological diseases with other complex diseases and disorders. 19
Figure S12: Squared effect sizes of GWAS hits for brain-related versus other traits. 20
Figure S13: Parameterizing the genetic architectures of complex traits (first half). 21
Figure S14: Parameterizing the genetic architectures of complex traits (second half). 22
Figure S15: Contrasting body composition-related traits with other quantitative traits. 23
Figure S16: Contrasting two birth coordinate traits with 151 selected quantitative traits. 24
Figure S17: Sibling GWAS show no systematic bias in median MAF of GWAS hits. 25
Figure S18: Contrasting urinary traits with serum creatinine and other quantitative traits. 26
Figure S19: LDL GWAS power loss after lower-tail binarization. 27
Figure S20: BMI GWAS power loss after binarization or downsampling. 28
Figure S21: HbA1c GWAS power loss after binarization or downsampling. 29
Figure S22: Diastolic blood pressure GWAS power loss after binarization or downsampling. 30
Figure S23: Systolic blood pressure GWAS power loss after binarization or downsampling. 31
Figure S24: Heel BMD T-score GWAS power loss after binarization or downsampling. 32
Figure S25: Comparing GWAS hits from three schizophrenia GWAS. 33
Figure S26: Comparing trait-specific $f(s)$ for brain-related versus non-brain-related traits. 34
Figure S27: Histograms of inferred evolutionary parameters. 35
Figure S28: Comparison of heritability estimates obtained in this study with recently reported whole-genome sequence-based heritability estimates. 36
Figure S29: Variation in MAF distributions of GWAS hits cannot be recovered by changing L. 37
Figure S30: Impact of GWAS ascertainment on simulations under different $f(s)$ distributions. 38
Figure S31: Whether or not a gene is expressed in the brain strongly predicts s_{het}. 39
Figure S32: Heatmap of pairwise genetic correlations across 32 independent traits. 40

Figure S33: Mean squared genic effects on independent brain-related versus other traits. . . . 41
Figure S34: Genes expressed in the brain tend to be longer and have longer intergenic lengths. . . . 42
Figure S35: Assuming no pleiotropy for variant effects fails to recapitulate the observed variation in MAF distributions of GWAS hits. . . . 43

Supplementary Tables

Table S1: Details of complex diseases and disorders included in this study. . . . 44
Table S2: Key parameters for complex diseases and disorders included in this study. . . . 45
Table S3: Comparing the study design of three schizophrenia GWAS. . . . 46
Table S4: Model fit of the normal distribution, the alpha model, and the Simons model. . . . 47

Box 1: The Simons *et al.* model of pleiotropic stabilizing selection

Core idea. Assuming pleiotropic stabilizing selection, the phenotypic effect of a variant can be represented as a vector in a high-dimensional trait space, with the strength of selection determined by the magnitude of the variant's effect in trait space. When the dimensionality of the trait space is sufficiently high, the conditional distribution of the effect size on a focal trait, $\beta \mid s$, is approximately normal. Consequently, we do not need to specify the remaining traits explicitly.

Key assumptions. We assume that most complex traits are under stabilizing selection, with trait means close to their respective optima. We further assume that variants are highly pleiotropic, each affecting at least 10 traits, and that the degree of pleiotropy is broadly consistent across GWAS hits for a given trait. Finally, we assume that GWAS hits can capture the genetic architecture of a trait and that the allele frequencies and effect sizes of GWAS-identified variants constitute a censored, but otherwise unbiased, sample of all the underlying causal variants.

Main results from the previous paper (1). Simons *et al.* fitted a generative version of the model to approximately independent GWAS hits for 95 quantitative traits in the UK Biobank, each with more than a hundred hits. They found that the inferred $f(s)$ are fairly similar across traits. Consequently, most of the variation in GWAS results across traits is driven by two scaling parameters, L and h^2/L.

New results from this study. We extended the model to accommodate binary traits and evaluated its fit. By grouping related traits and inferring a shared $f(s)$, we were also able to include traits with fewer than a hundred hits. With the expanded set of quantitative and binary traits, we included more brain-related traits and examined how their model parameters differ from those of other traits.

Supporting Information Text

1. GWAS power.

Quantitative trait GWAS power. In a linear regression model of the form $Y \sim X\beta + \epsilon$, the estimated effect sizes are approximately distributed as $\hat{\beta} \sim \mathcal{N}(\beta, SE^2)$ (2). For a quantitative trait GWAS, Y is a vector of standardized phenotype values, X is a vector of genotype values at a focal SNP, β is the additive effect size per copy of the effect allele, and the residual term $\epsilon \sim N(0, \sigma^2)$ captures the background genetic and environmental noise. If H_1 is true, i.e., $\beta \neq 0$, Z^2 approximately follows a non-central chi-square distribution, $Z^2 \sim \chi_1^2((\frac{\beta}{SE})^2)$. GWAS power is determined by this non-centrality parameter $(\frac{\beta}{SE})^2$. Furthermore, if N is the total number of individuals in the study, and X_i is centered so that $\bar{X} = 0$,

$$SE^2 \approx Var(\hat{\beta}) = \frac{\sigma^2}{NVar(X)} = \frac{\sigma^2}{2Np(1-p)},$$

where the last step assumes Hardy-Weinberg equilibrium at this locus. Assuming σ^2 is approximately the phenotypic variance, V_p, and the trait is standardized to have unit variance, we get

$$\text{quantitative trait GWAS power} \propto f\left(2Np(1-p)\beta^2\right). \qquad [1]$$

Binary trait GWAS power. A case-control GWAS typically assumes a logistic regression model of the form

$$\ln(\frac{P}{1-P}) \sim \zeta X + \epsilon, \qquad [2]$$

where $P = Prob(Y = 1)$, and ζ is the effect size of allele 1 measured on the log odds ratio scale.

	Case	Control
Risk allele count	a	b
Non-risk allele count	c	d

Contingency table.

Given a contingency table as shown above and assuming that all cell counts are non-zero,

$$\hat{\zeta} \sim N(\zeta, \frac{1}{a} + \frac{1}{b} + \frac{1}{c} + \frac{1}{d}),$$

approximately. Under the null hypothesis that $\zeta = 0$, we have $E(a) = 2M\omega p$ given that M is the number of cases plus the number of controls, and ω is the sample prevalence. Similarly, $E(b) = 2M(1-\omega)p$, $E(c) = 2M\omega(1-p)$, and $E(d) = 2M(1-\omega)(1-p)$. Plugging these expected values into the previous formula, it follows that $\hat{\zeta} \sim N(\zeta, \frac{1}{2Mp(1-p)\omega(1-\omega)})$. Therefore, we arrive at

$$\text{binary trait GWAS power} \propto f\left(2Mp(1-p)\zeta^2\omega(1-\omega)\right). \qquad [3]$$

2. Relating effect sizes.

Note that the effect sizes obtained from a quantitative trait GWAS, β's, are measured in standardized units on an additive scale, whereas the effect sizes obtained from a binary trait GWAS, ζ's, are measured on a log odds ratio scale. Previous studies found that if effect sizes are sufficiently small, then the relationship between these two effect sizes is

$$\beta \approx \frac{K(1-K)}{\phi(T)}\zeta, \qquad [4]$$

where K is the prevalence of the disease in the population and $\phi(T)$ is the standard normal density at the liability threshold (3, 4).

Here, we provide a derivation of this relationship. In a population carrying a variant with effect size β, the disease liability distribution is shifted to the right compared with a population without the variant (Figure S1). When the effect size, ζ, is sufficiently small, using Equation 2 we find that

$$\frac{K'}{1-K'} = \frac{K + \Delta K}{1 - K - \Delta K} = \frac{K}{1-K}e^{\zeta} \approx \frac{K}{1-K}(1+\zeta),$$

which implies that

$$\Delta K \approx \frac{K(1-K)\zeta}{1+K\zeta} \approx K(1-K)\zeta.$$

In turn, when β is sufficiently small, the increase in prevalence can be approximated by

$$\Delta K \approx \beta \times \phi(T),$$

where $\phi(T)$ is the density at the liability threshold. The relationship between effect sizes on the liability scale and on the log odds ratio scale (Equation 4) follows from these expressions. When effect sizes are sufficiently large, this approximation breaks down, and ΔK depends on the shape of the liability distribution, which is typically assumed to be standard normal.

3. Binarizing and downsampling a quantitative trait.

Plugging Equation 4 into Equation 3, we can re-write binary trait GWAS power as

$$\text{binary trait GWAS power} \propto f\left(2Mp(1-p)\beta^2\omega(1-\omega)\frac{\phi(T)^2}{K^2(1-K)^2}\right). \quad [5]$$

To better understand how to relate the GWAS power of a quantitative trait and a binary trait, we next binarized a quantitative trait and ran a case-control GWAS. In the simplest case, we sampled the full set of cases and controls after binarization, that is, $N = M$ and $K = \omega$. According to Equation 5 and Equation 1, we see that binarizing a quantitative trait reduces GWAS power, with the case-control GWAS retaining $\frac{\phi(T)^2}{K(1-K)} * 100\%$ of the original GWAS power (Figure S2).

Furthermore, the loss in GWAS power after trait binarization can be replicated by downsampling the original trait to a new sample size N', given by Equation 1. The obtained Z-scores after binarization and downsampling both will be deflated by a factor of

$$Z_{after} = \sqrt{\frac{N'}{N}} Z_{before} = \sqrt{\frac{M\omega(1-\omega)\frac{\phi(T)^2}{K^2(1-K)^2}}{N}} Z_{before}. \quad [6]$$

Equation 6 gives the predicted slopes, shown as solid colorful lines in Figures 2C and D.

4. Insights from the formula for estimating N'.

The formula we derived for transferring the sample size of a case-control GWAS to the effective sample size on the liability scale, $N' \approx M\omega(1-\omega)\frac{\phi(T)^2}{K^2(1-K)^2}$, can be decomposed into two parts. From the first factor, $\omega(1-\omega)$, it is evident that the power of a case-control GWAS is maximized when the sample prevalence is 50%. The second factor, $\frac{\phi(T)^2}{K^2(1-K)^2}$, is the main reason why the estimated N' for the current schizophrenia GWAS, 192,273, is larger than its original sample size of $M = 130{,}644$ (Materials and Methods, Table S2). For a given case-control study, i.e., when $M\omega(1-\omega)$ is fixed, a higher statistical power is achieved when the disease is less prevalent (Figure S3). One can also regard this as rewarding the extra data collection effort to recruit the same number of cases for a rarer disease.

Combining these elements, we arrived at a strategy for optimizing GWAS design. Suppose we want to conduct a GWAS on a quantitative trait, but with a constraint on budget. In other words, there is a limit on the number of individuals we can genotype or sequence. Under these circumstances, the optimal approach that maximizes GWAS power is to sample X individuals in the upper $K\%$ of the phenotypic distribution, match with an additional X individuals sampled from the lower $(1-K)\%$ of the distribution, and conduct a case-control GWAS. As long as K is below 8.5%, the second factor will be larger than 4 (Figure S3), and therefore the resulting case-control GWAS will have greater statistical power than conducting a quantitative trait GWAS with $2X$ individuals.

5. Urinary biomarkers as an additional check against confounding.

We were intrigued by urinary biomarkers, some of which were classified by our pipeline as brain-related and are less obviously prone to confounding from population stratification, assortative mating, or indirect genetic effects as compared to behavioral-cognitive traits. We noticed that creatinine and sodium levels in urine are classified as brain-related, while the level of creatinine in blood is not (Dataset S1 and Figure S18A). Notably, in clinical practice, serum creatinine assesses the kidney's normal function of eliminating waste products from muscle metabolism, whereas urinary creatinine is often used to normalize other urinary biomarkers, given that creatinine excretion is kept at a relatively constant rate (5). Thus, unadjusted urinary creatinine and sodium concentrations are indicative of hydration status and urine flow rate, which involves coordination between the brain and bladder (6). GWAS results of these urinary traits more closely resemble those of other brain-related traits than those of serum creatinine, which again supports that traits mediated through the CNS share similar genetic architectures (Figure S18B). Overall, this suggests that confounding is not driving the distinct patterns we see for the brain-related traits in our analyses, including the behavioral-cognitive ones.

6. Evaluating model fit of the extended Simons *et al.* model.

We fitted the extended model to six complex diseases and disorders that have more than 100 approximately independent hits (trait-specific $f(s)$ shown in Figure S4), as previous work suggests the inference of evolutionary parameters should be well powered in this case (1). Two of the six traits, schizophrenia and major depression, are brain-related. Following the approaches in the previous Simons *et al.* paper for evaluating model fit to quantitative traits, we considered alternative models for comparison: fitting effect sizes with a normal distribution, and the α-model (7), which imposes a negative relationship between effect sizes and allele frequencies.

We quantified the goodness-of-fit of the different models by computing a residual p-value, defined previously as $\Pr\left(|z| > |z_i| \,\big|\, |z_i| > 5.45,\, p_i,\, \text{model}\right)$ (1). If we correctly model the distribution of z-scores among GWAS hits, then these residual

p-values should be uniformly distributed between 0 and 1. To avoid overfitting, we split the genome into approximately independent blocks (8), each time inferring the model on 90% of the blocks and computing residual p-values for the held-out 10%. Across diseases, our pleiotropic stabilizing selection model provides a better fit to the residual p-values than the two simpler heuristic models (Figure S5). We next conducted Kolmogorov-Smirnov tests to determine whether the overall distribution of residual p-values for each trait matches the expected uniform distribution. At a false discovery rate of 0.05, we find that the simpler heuristic models provide a poor fit to the data; notably, we can reject the normal model for 5 of the 6 diseases, the α-model for 4, and the pleiotropic stabilizing selection model for none (Table S4).

Supplementary Figures

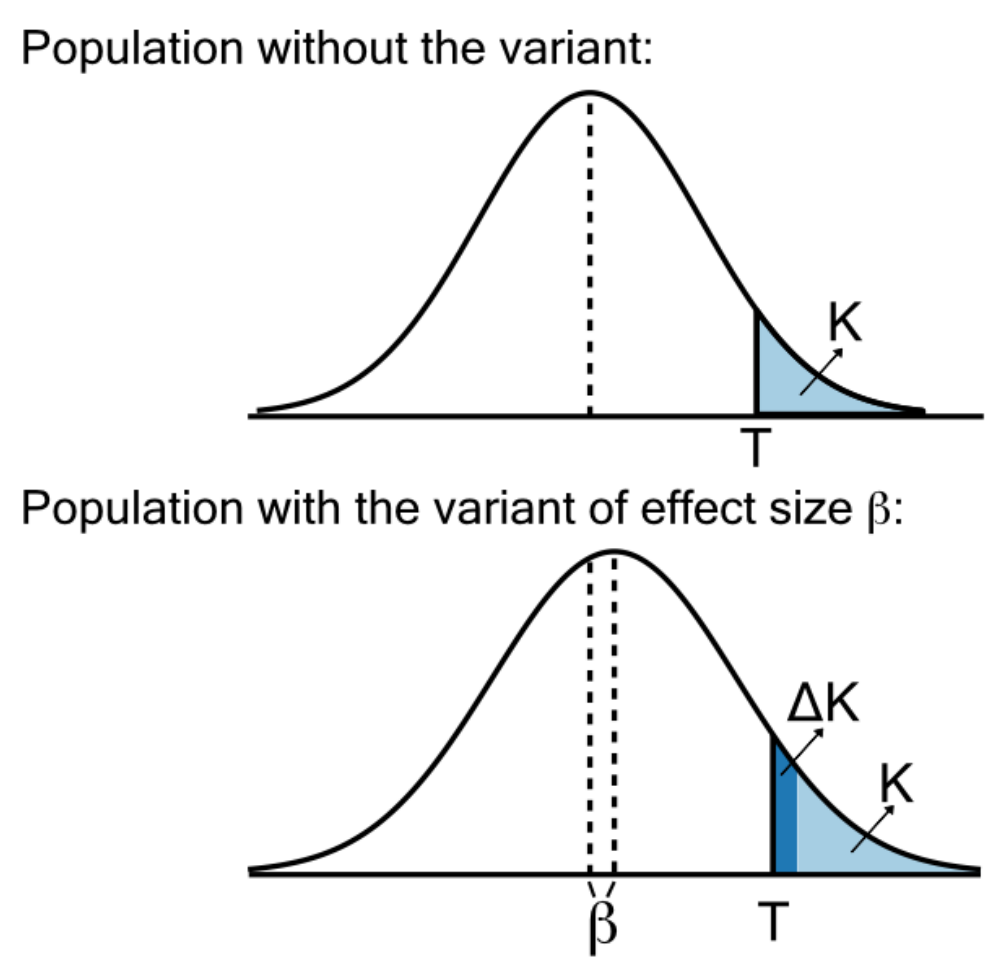

Fig. S1. Relating effect sizes with the liability threshold model. Our derivation here assumes that effect sizes (both β and ζ) are small.

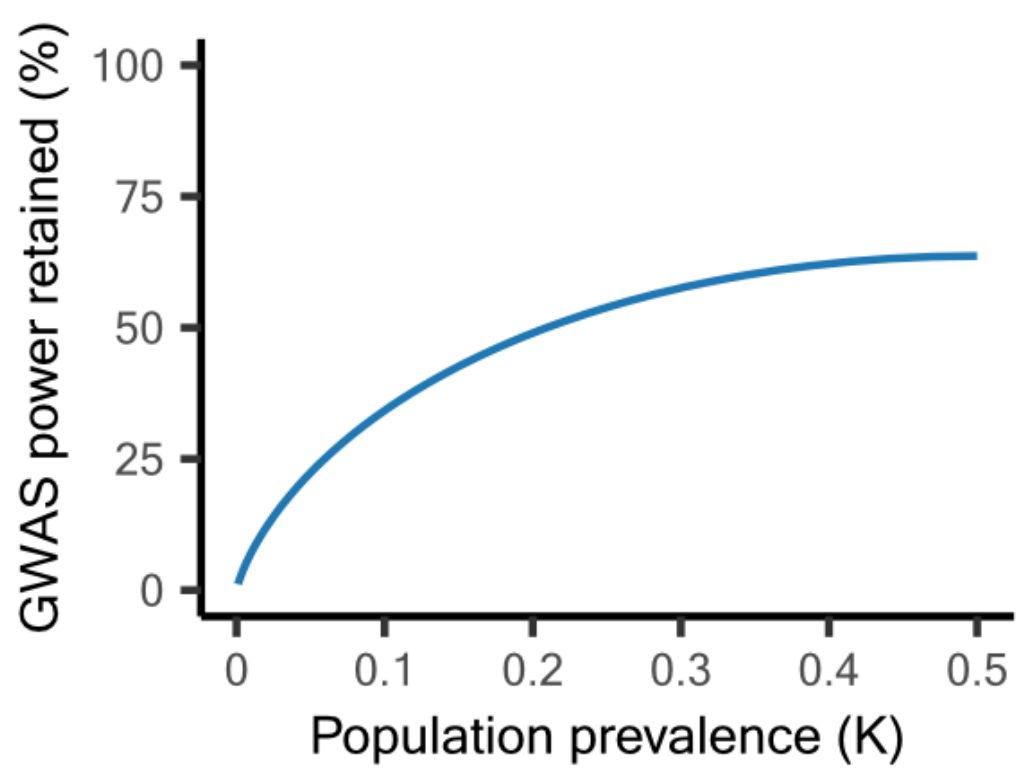

Fig. S2. Reduction in GWAS power after converting a quantitative trait to a binary trait. We assume that the sample size remains the same and that the population prevalence matches the sample prevalence. $\phi(T)$ denotes the density at the liability threshold, assuming liability follows a standard normal distribution.

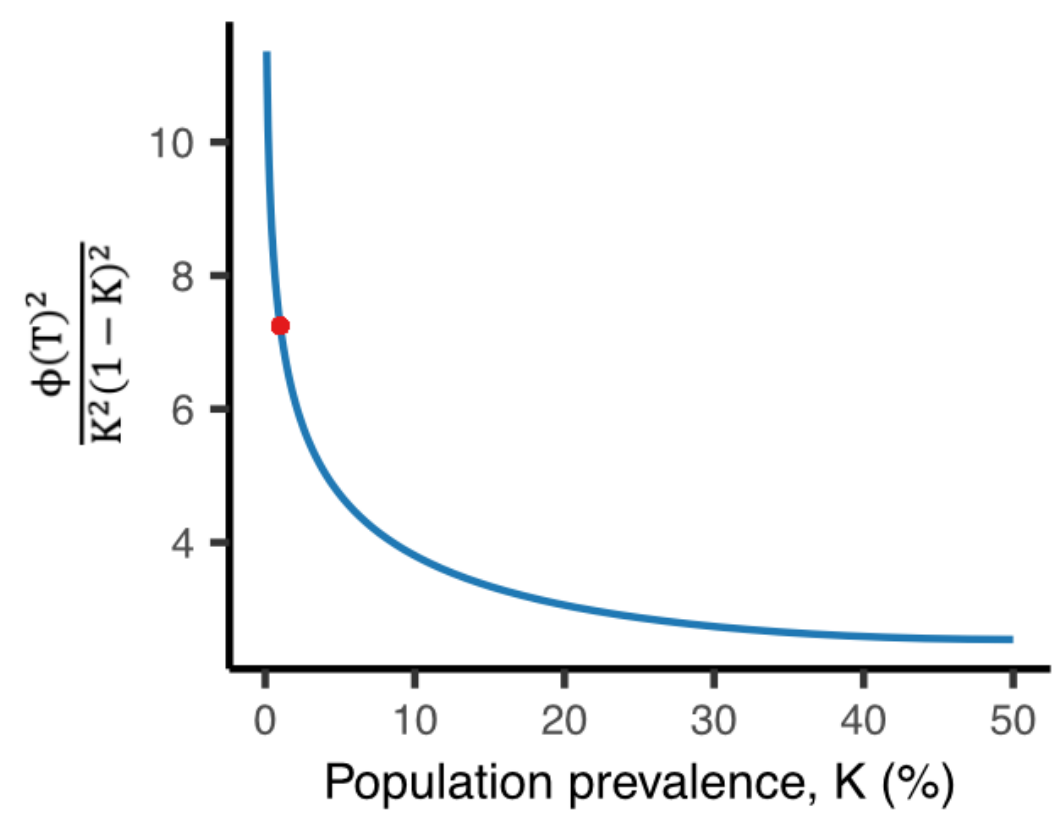

Fig. S3. A larger sample size is needed on the liability scale when the disease is rarer. The red point is placed at $K = 1\%$, the assumed population prevalence for schizophrenia (9).

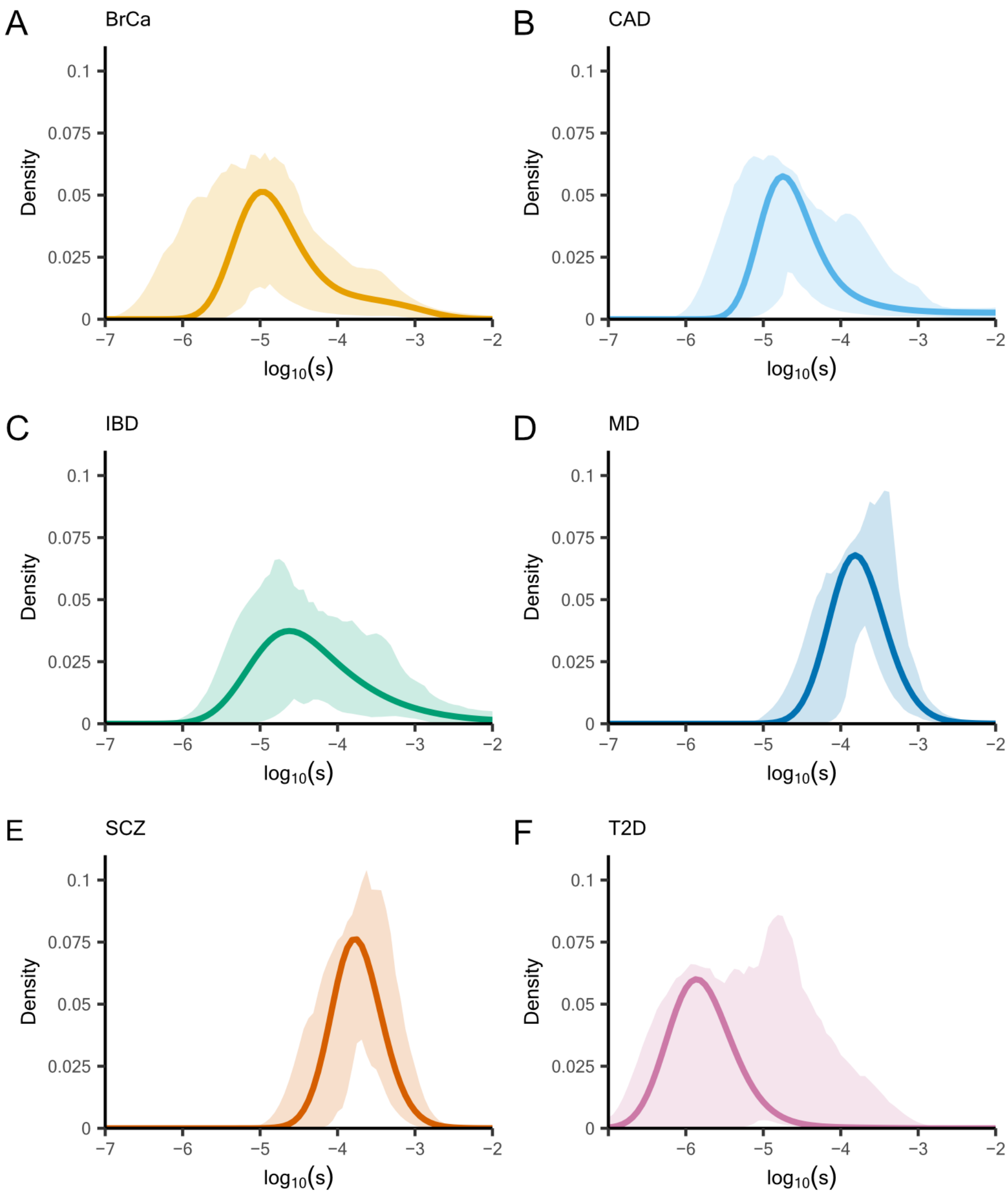

Fig. S4. Trait-specific distributions of selection coefficients inferred from *A)* breast cancer *B)* coronary artery disease *C)* inflammatory bowel disease *D)* major depression *E)* schizophrenia *F)* type 2 diabetes GWAS hits, with 90% confidence envelopes shown.

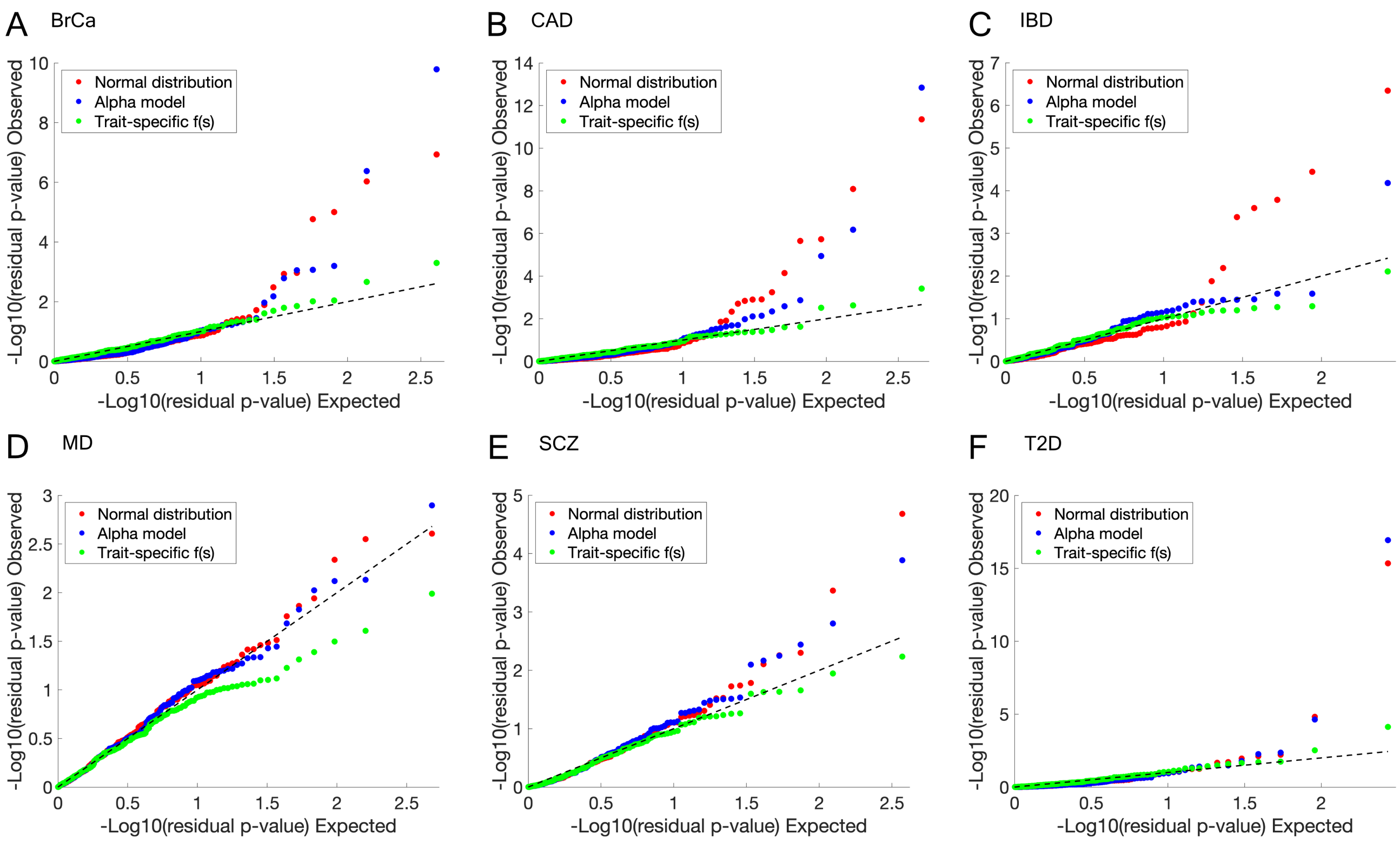

Fig. S5. QQ plots of residual p-values for ***A)*** *breast cancer* ***B)*** *coronary artery disease* ***C)*** *inflammatory bowel disease* ***D)*** *major depression* ***E)*** *schizophrenia* ***F)*** *type 2 diabetes,* **evaluated under three models: the normal distribution, the alpha model, and the adapted Simons model, which infers trait-specific $f(s)$.**

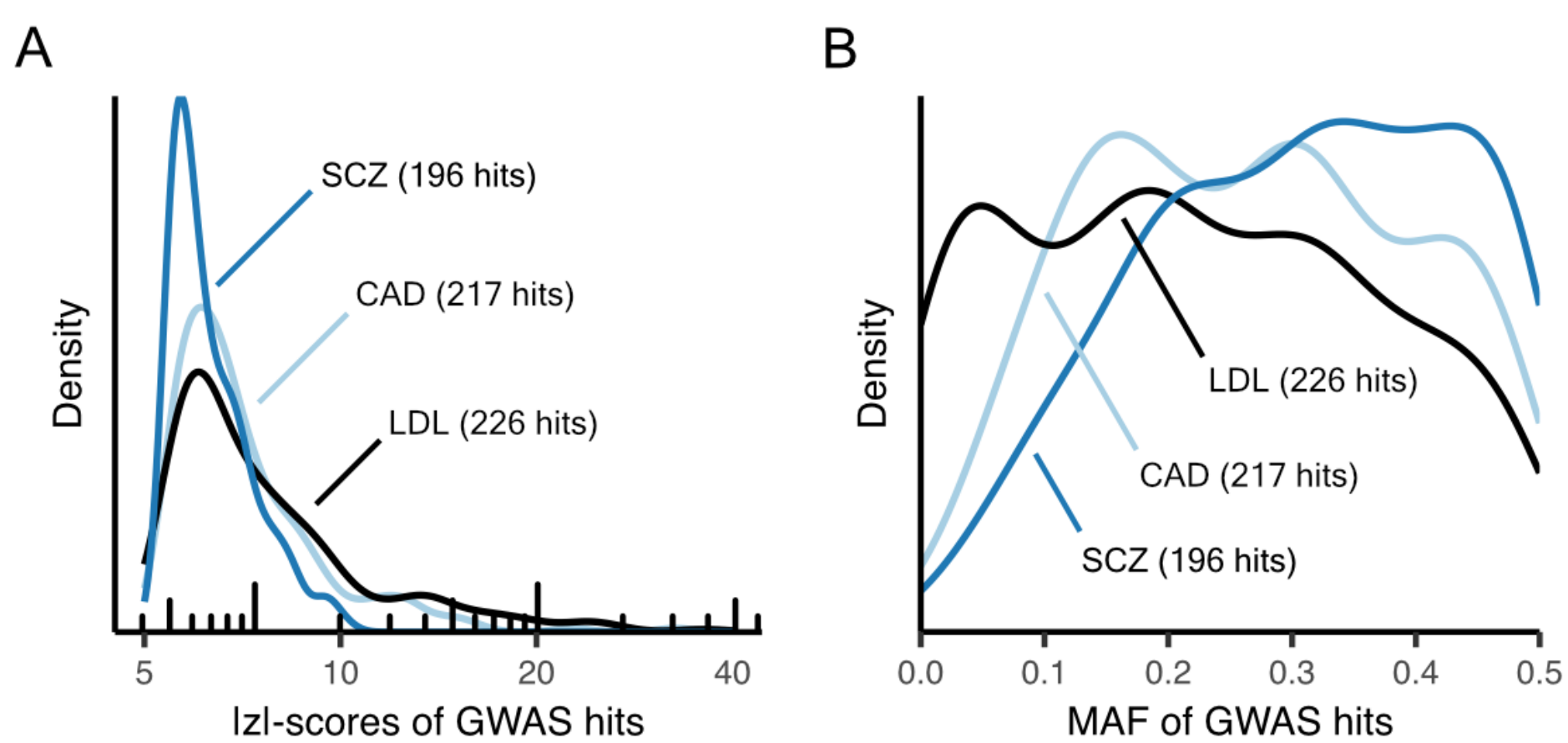

Fig. S6. Schizophrenia GWAS hits have a narrow distribution of $|z|$-scores and high MAFs. ***A)*** *Density of COJO hit absolute z-scores.* ***B)*** *Density of COJO hit MAF.*

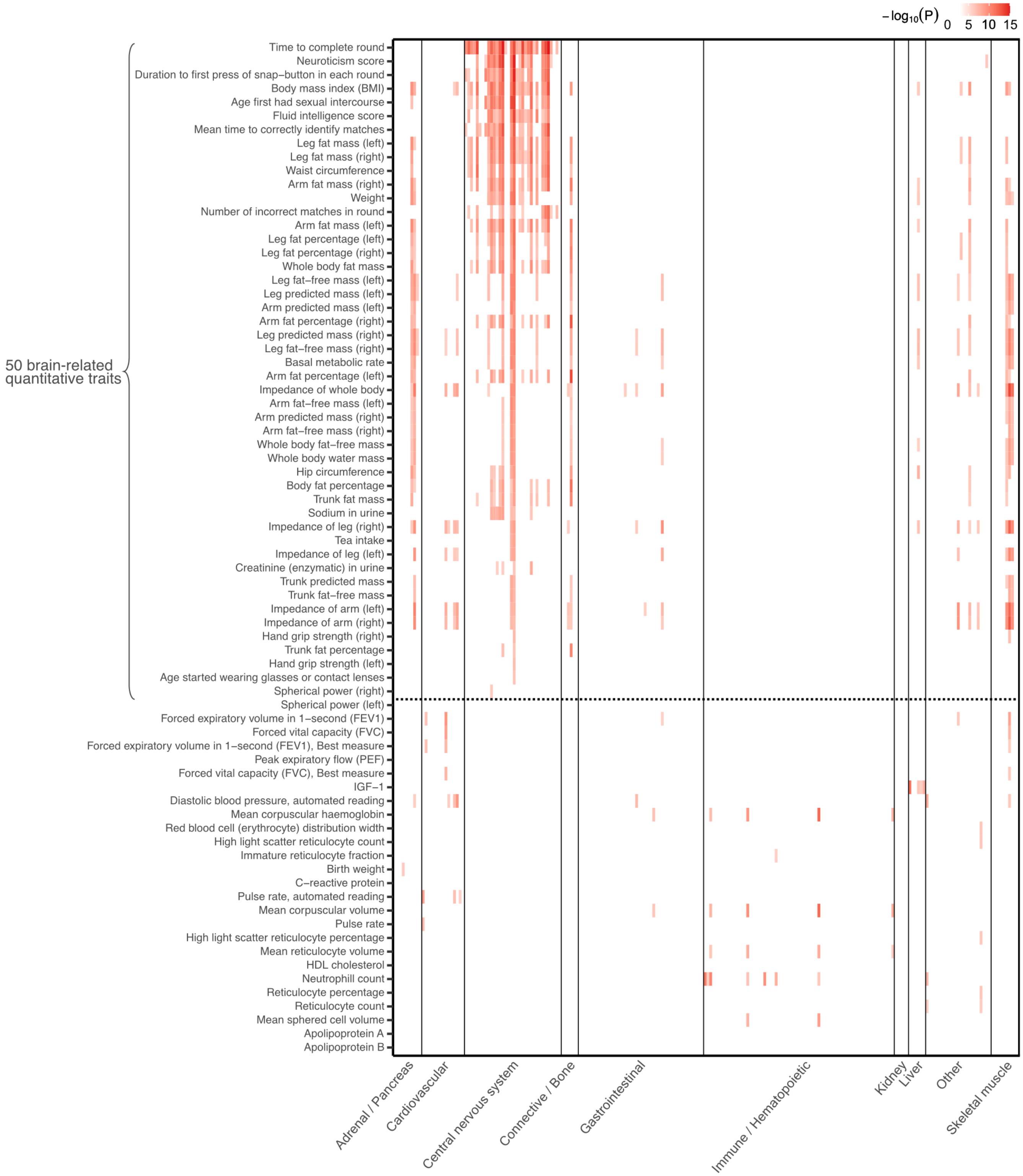

Fig. S7. S-LDSC functional enrichment analyses on 151 quantitative traits (first half). *Each row consists of 220 cell types from 10 categories, colored by* $-log_{10}(p)$ *for the coefficient* τ *in the S-LDSC model (10, 11) if* $-log_{10}(p)$ *passes the Bonferroni-corrected threshold* $-log_{10}(0.05/(220 \times 164))$*. Traits are ordered by decreasing significance of the meta-analyzed p-values for CNS enrichment (Materials and Methods). The top 50 traits pass the significance threshold of* $-log_{10}(0.05/(10 \times 164))$ *and are therefore classified as brain-related.*

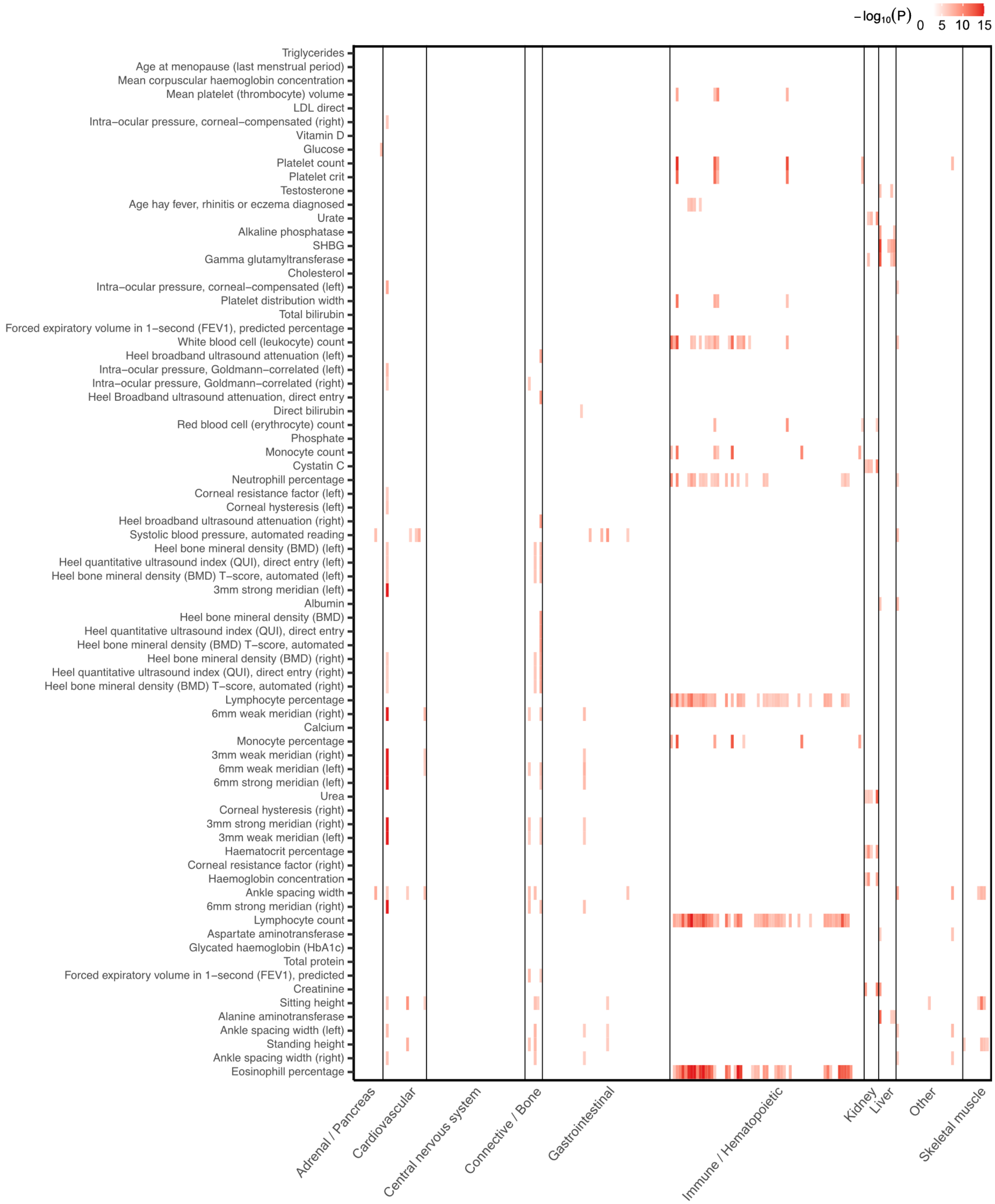

Fig. S8. S-LDSC functional enrichment analyses on 151 quantitative traits (second half). *Each row consists of 220 cell types from 10 categories, colored by* $-log_{10}(p)$ *for the coefficient* τ *in the S-LDSC model (10, 11) if* $-log_{10}(p)$ *passes the Bonferroni-corrected threshold* $-log_{10}(0.05/(220 \times 164))$. *Traits are ordered by decreasing significance of the meta-analyzed p-values for CNS enrichment (Materials and Methods). All traits in this figure are classified as non-brain-related.*

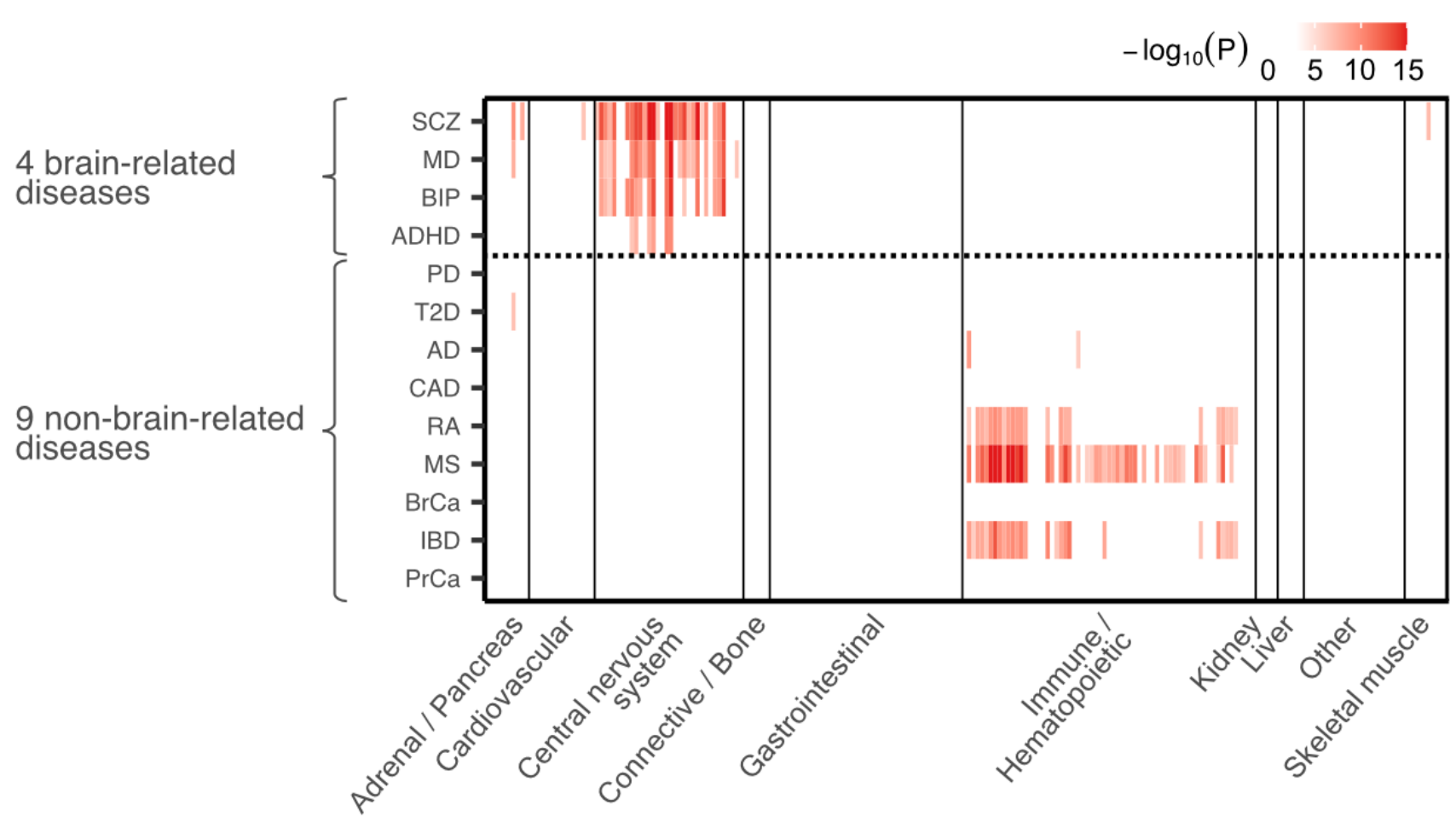

Fig. S9. Functional enrichment analyses with S-LDSC on 13 human complex diseases and disorders. *Each row consists of 220 cell types from 10 categories, colored by* $-log_{10}(p)$ *of the coefficient* τ *in the S-LDSC model (10, 11) if* $-log_{10}(p)$ *passes the Bonferroni correction threshold* $-log_{10}(0.05/(220\times164))$*. Traits are ordered by decreasing significance of the meta-analyzed p-values for CNS enrichment (Materials and Methods). The top 4 traits pass the significance threshold of* $-log_{10}(0.05/(10\times164))$ *and are therefore classified as brain-related. Full names of the traits can be found in Table S1.*

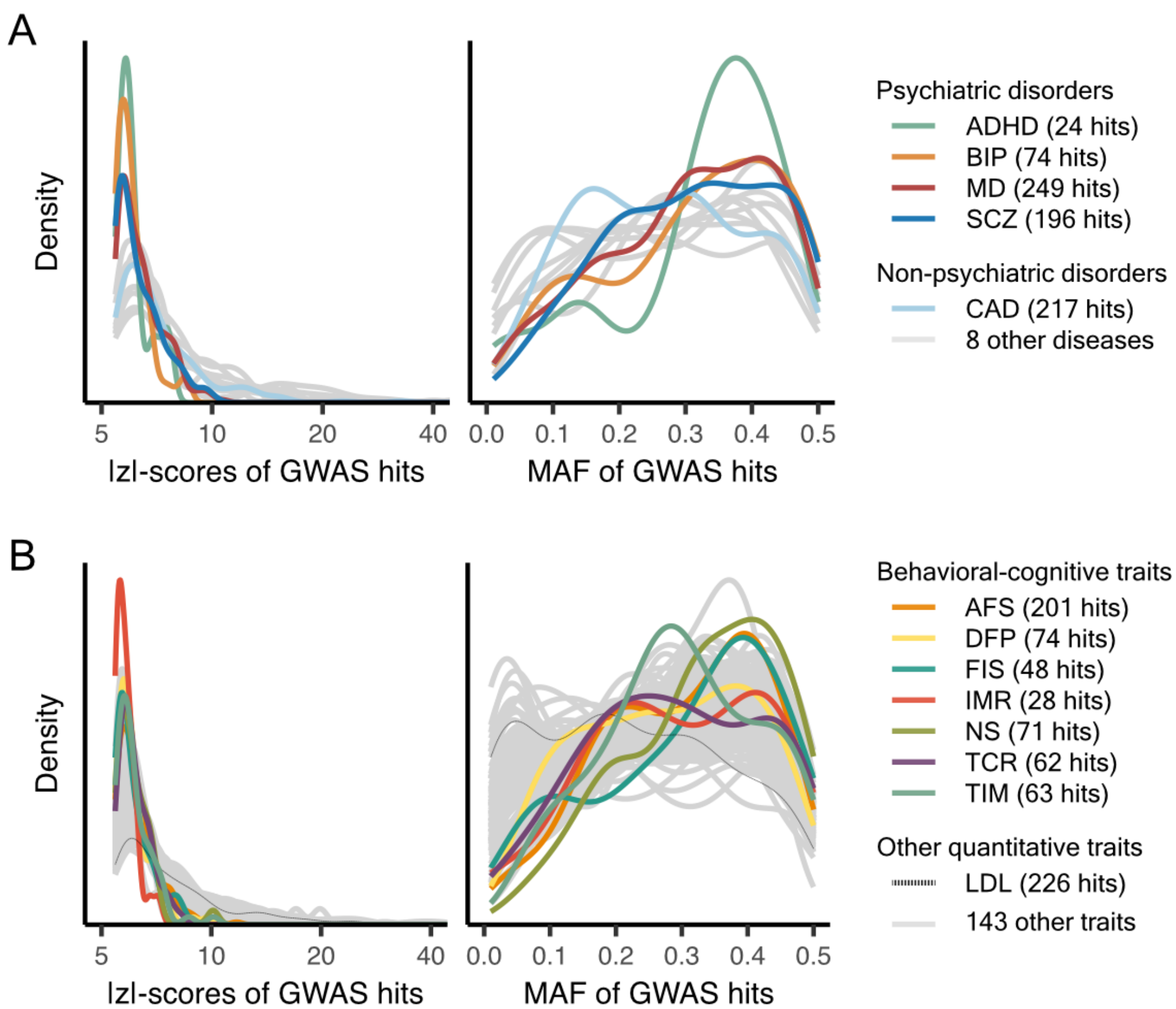

Fig. S10. GWAS hits for brain-related traits have a narrow distribution of $|z|$-scores and high MAFs. ***A)*** *Density of COJO hit absolute z-scores.* ***B)*** *Density of COJO hit MAF.*

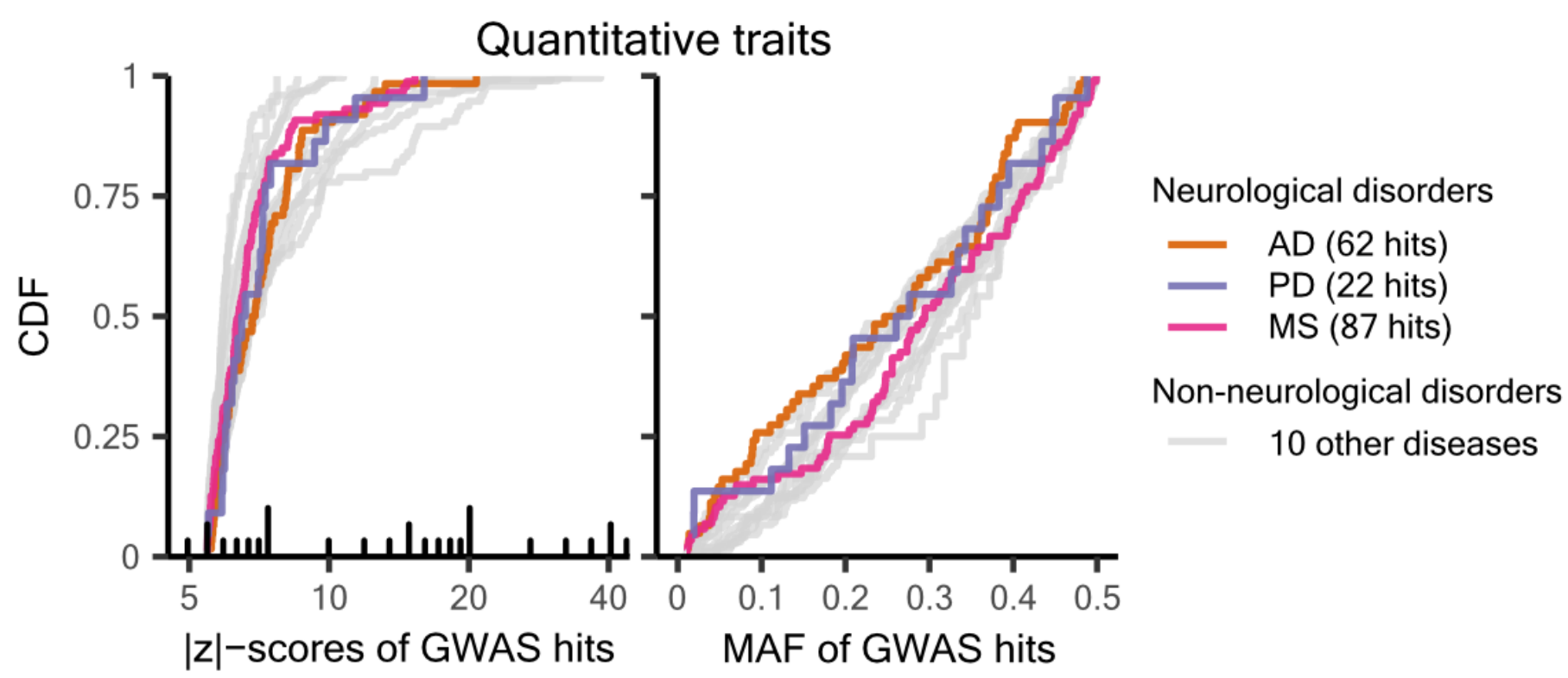

Fig. S11. Contrasting the GWAS hits for neurological diseases with those for 10 other human complex diseases and disorders. *The full list of diseases and disorders can be found in Table S1. AD: Alzheimer's disease; PD: Parkinson's disease; MS: multiple sclerosis.*

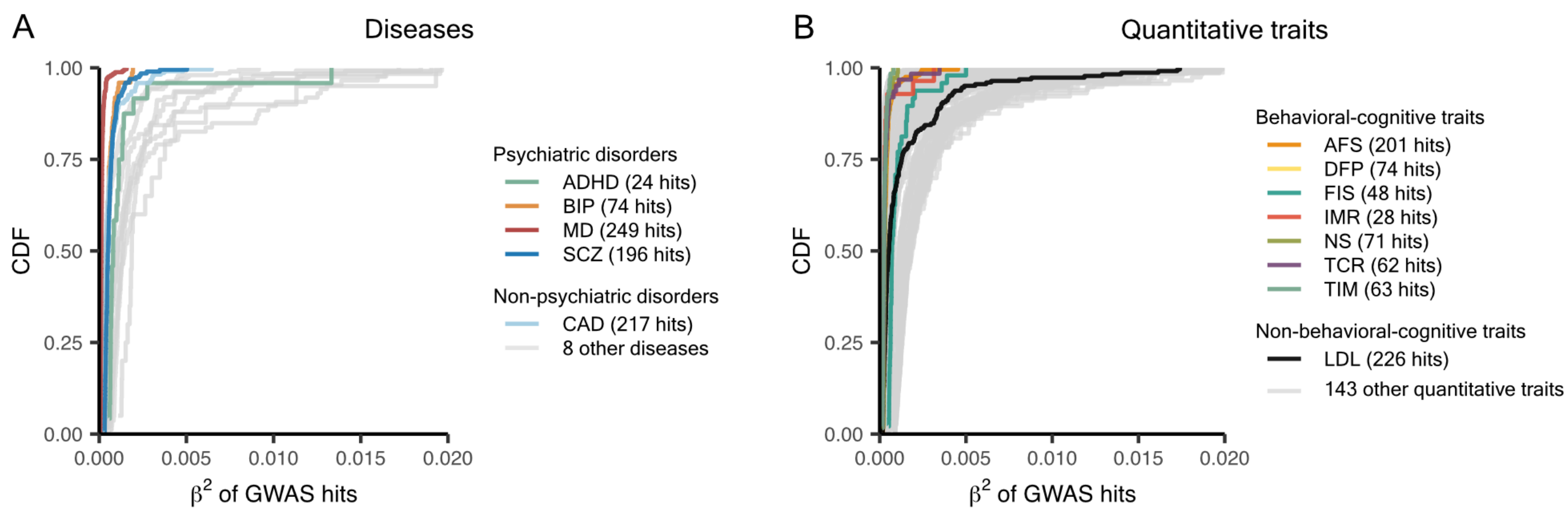

Fig. S12. Squared effect sizes of GWAS hits for brain-related versus non–brain-related traits. ***A)*** *Psychiatric disorders compared with nine other complex diseases. Effect sizes reported on the log odds ratio scale (ζ) are transformed to the liability scale (β) with Equation 4. Details of the derivation are provided in the Supplementary notes, Section 2. When applying Equation 4, we assumed disease prevalence as reported in Table S2.* ***B)*** *Behavioral-cognitive traits compared with 144 other quantitative traits.*

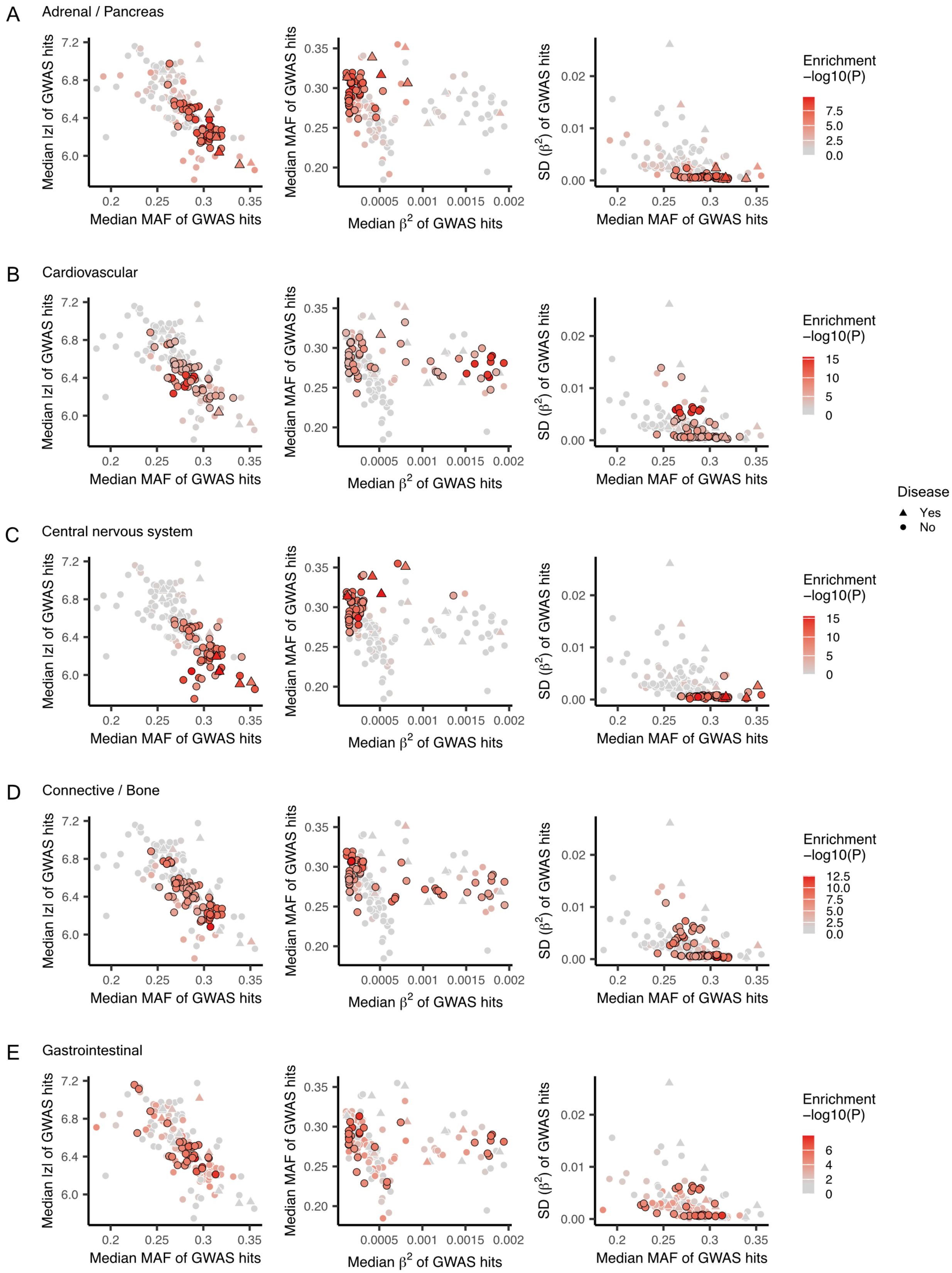

Fig. S13. Parameterizing the genetic architectures of complex traits (first half). *Pairwise combinations of four GWAS hit metrics — median MAF, median $|z|$-score, median β^2, and standard deviation of β^2 — were used to parameterize trait architectures. All 164 traits included in this study are colored by meta-analyzed S-LDSC p-values for functional enrichment in the* ***A)*** *Adrenal / Pancreas* ***B)*** *Cardiovascular* ***C)*** *Central nervous system* ***D)*** *Connective / Bone* ***E)*** *Gastrointestinal category. Traits with p-values exceeding the significance threshold of $0.05/(10 \times 164)$ are outlined with black borders.*

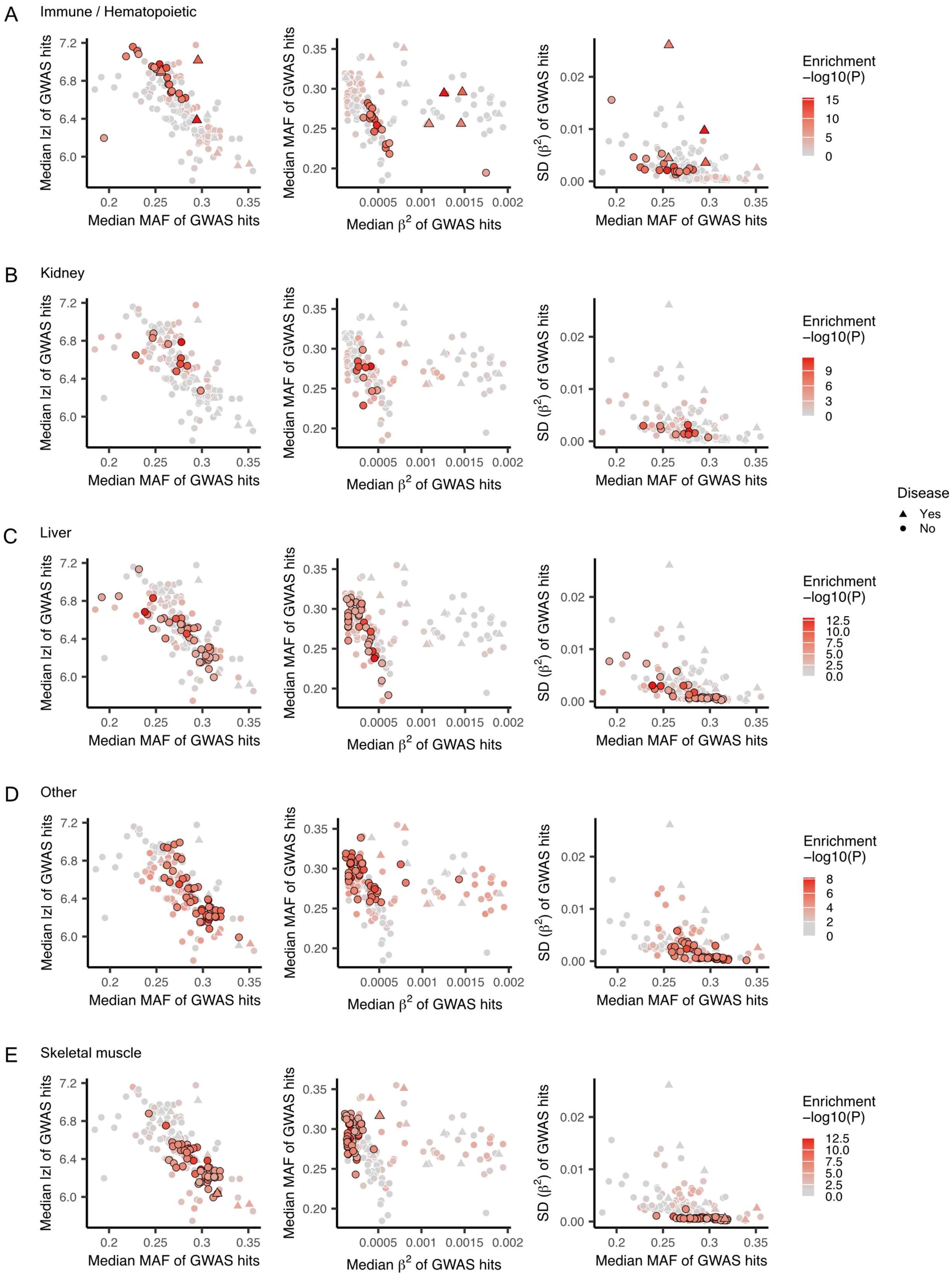

Fig. S14. Parameterizing the genetic architectures of complex traits (second half). *Pairwise combinations of four GWAS hit metrics — median MAF, median $|z|$-score, median β^2, and standard deviation of β^2 — were used to parameterize trait architectures. All 164 traits included in this study are colored by meta-analyzed S-LDSC p-values for functional enrichment in the* ***A)*** *Immune / Hematopoietic* ***B)*** *Kidney* ***C)*** *Liver* ***D)*** *Other* ***E)*** *Skeletal muscle category. Traits with p-values exceeding the significance threshold of $0.05/(10 \times 164)$ are outlined with black borders.*

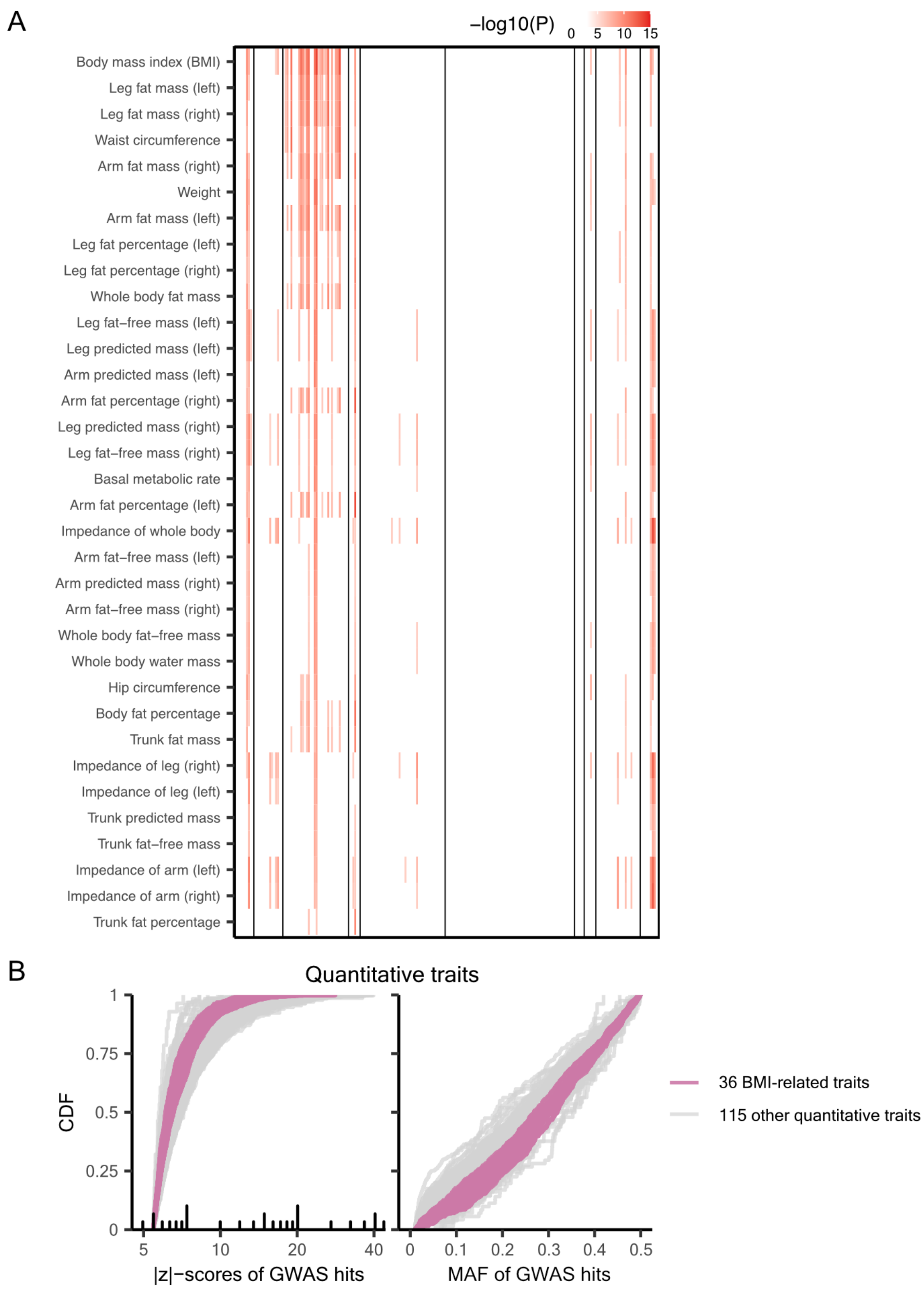

Fig. S15. Contrasting body composition-related traits with other quantitative traits. ***A)*** *S-LDSC profiles of the 36 body composition-related traits extracted from Figure S7.* ***B)*** *CDF curves for GWAS hits. The UK Biobank data fields for the 36 body composition–related traits are 48, 49, 21001, 21002, 23098–23102, and 23104–23130. Gray lines correspond to the rest of the 115 quantitative traits listed in Dataset S1.*

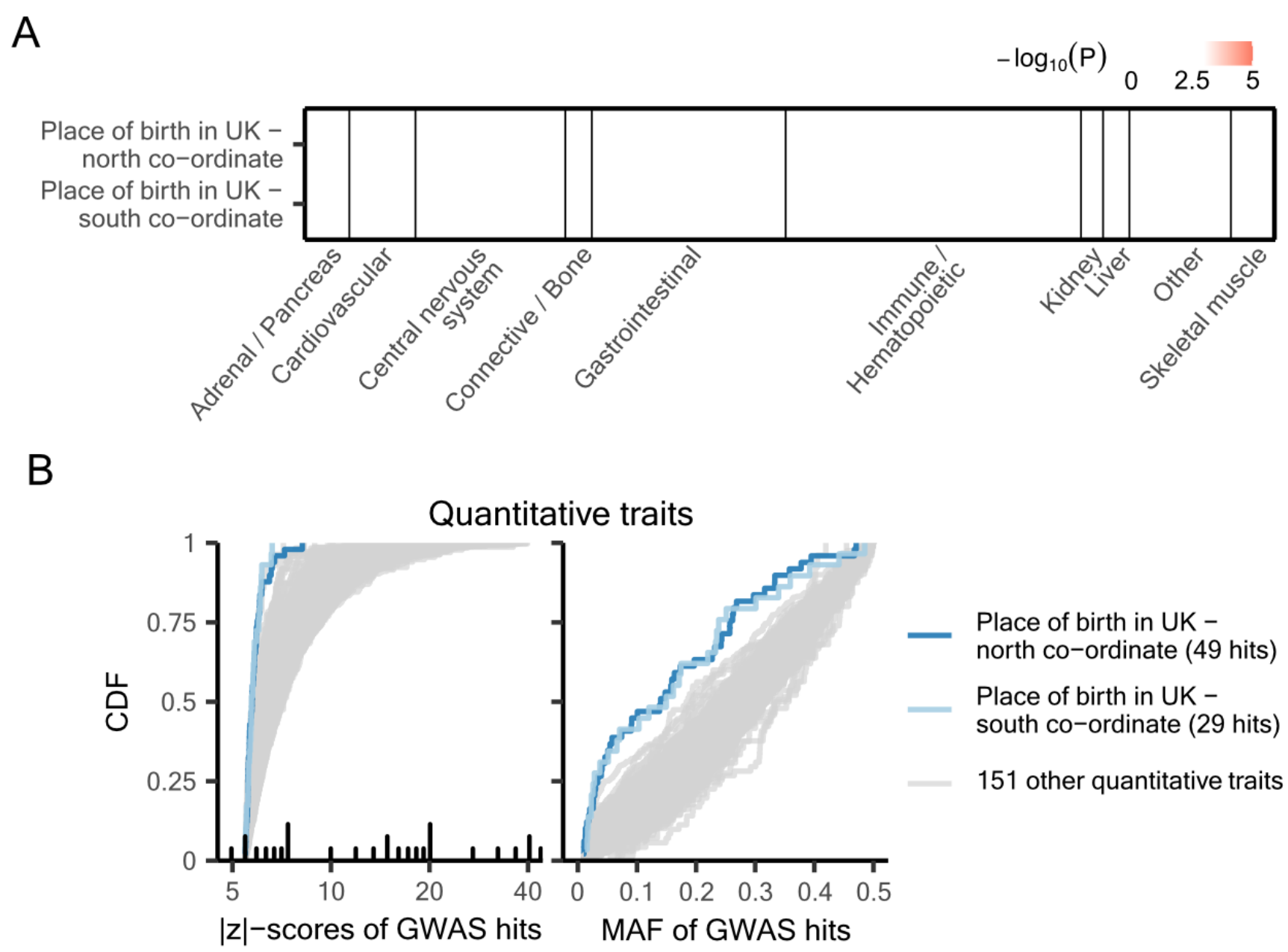

Fig. S16. Contrasting two birth coordinate traits with 151 selected quantitative traits. ***A)*** *S-LDSC enrichment results.* ***B)*** *CDF curves for GWAS hits. UK Biobank data fields are 129 for Place of birth in UK - north co-ordinate and 130 for Place of birth in UK - south co-ordinate. Same as the other quantitative traits, GWAS summary statistics for these two birth coordinate traits were downloaded from the Neale Lab (12), with 20 genotyping PCs included as covariates. The full list of quantitative traits can be found in Dataset S1.*

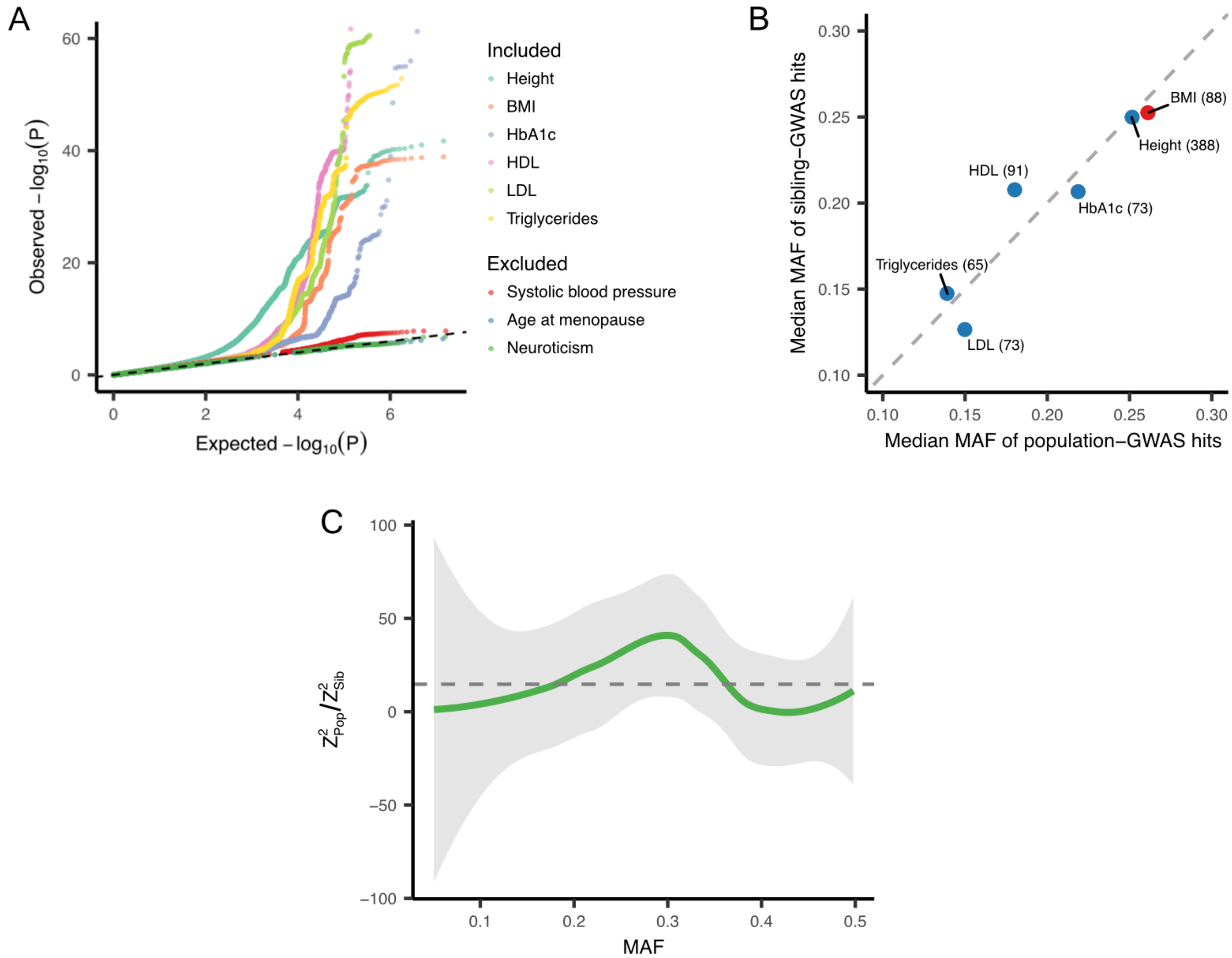

Fig. S17. Analyses of sibling GWAS show no evidence of systematic bias in the median MAF of GWAS hits, a key summary of trait architecture. ***A)*** *QQ plot of sibling GWAS summary statistics from Howe et al. (13), restricted to the nine traits that overlapped between their study and ours. The y-axis is restricted to the range* $[0, 60]$. ***B)*** *Median MAF of approximately independent hits identified from population and sibling GWAS. We selected six traits based on the QQ plot, applied a significance threshold of* 10^{-5}*, used distance-based clumping at 500 kb, and retrieved allele frequencies from the UK Biobank. Brain-related traits are shown in red and non-brain traits in blue. Population GWAS summary statistics are also from Howe et al. (13).* ***C)*** *Significant hits for neuroticism score used in this study show no MAF-dependent inflation in the Howe et al. population GWAS relative to the sibling GWAS. The green line is a LOESS curve, and the gray dashed line is plotted at the mean value.*

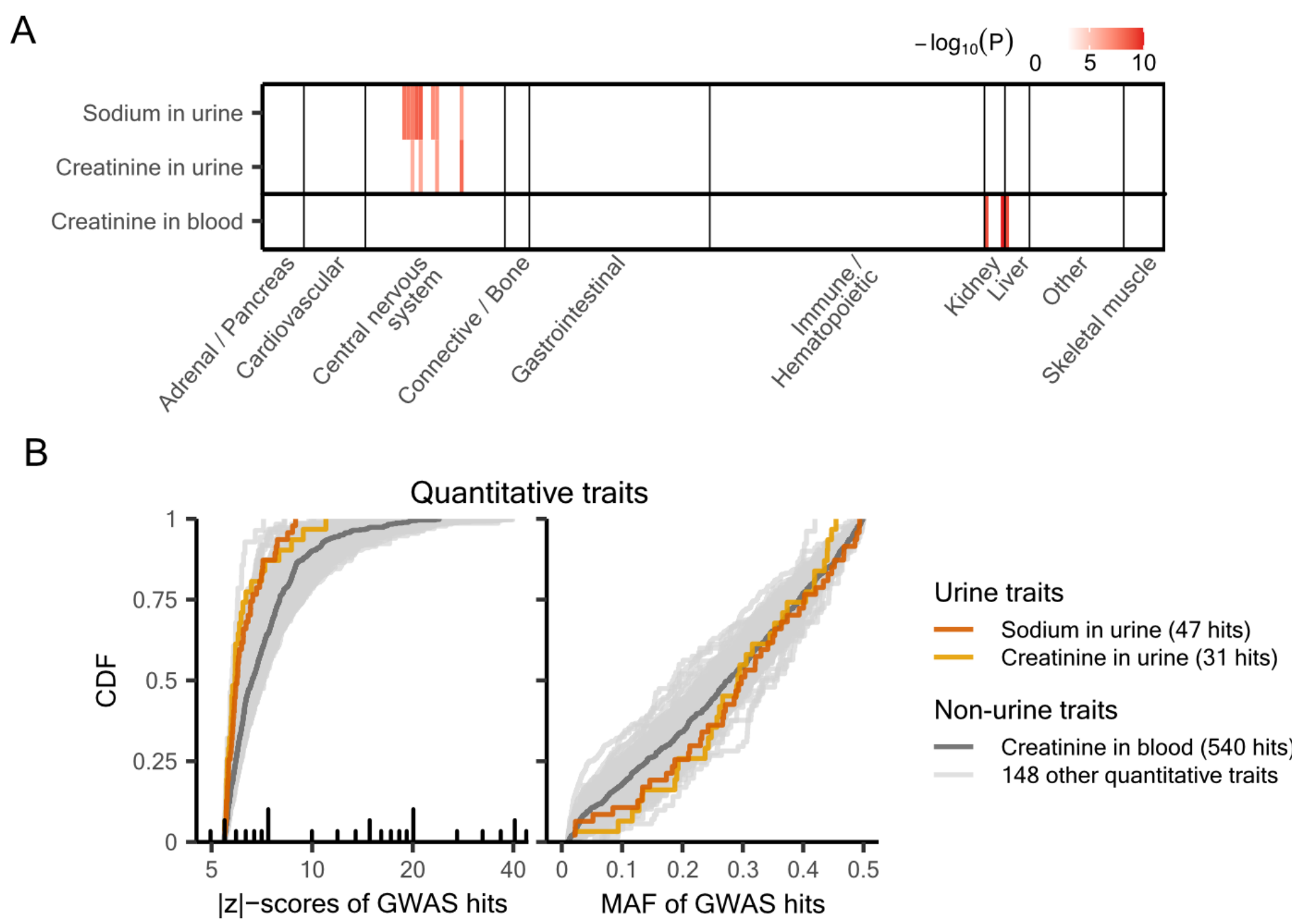

Fig. S18. Contrasting urinary traits with serum creatinine (and 148 other quantitative traits). ***A)*** *S-LDSC enrichment results.* ***B)*** *CDF curves for GWAS hits. The full list of quantitative traits can be found in Dataset S1. UK Biobank data fields are 30530 for sodium in urine, 30510 for creatinine in urine, and 30700 for creatinine in blood.*

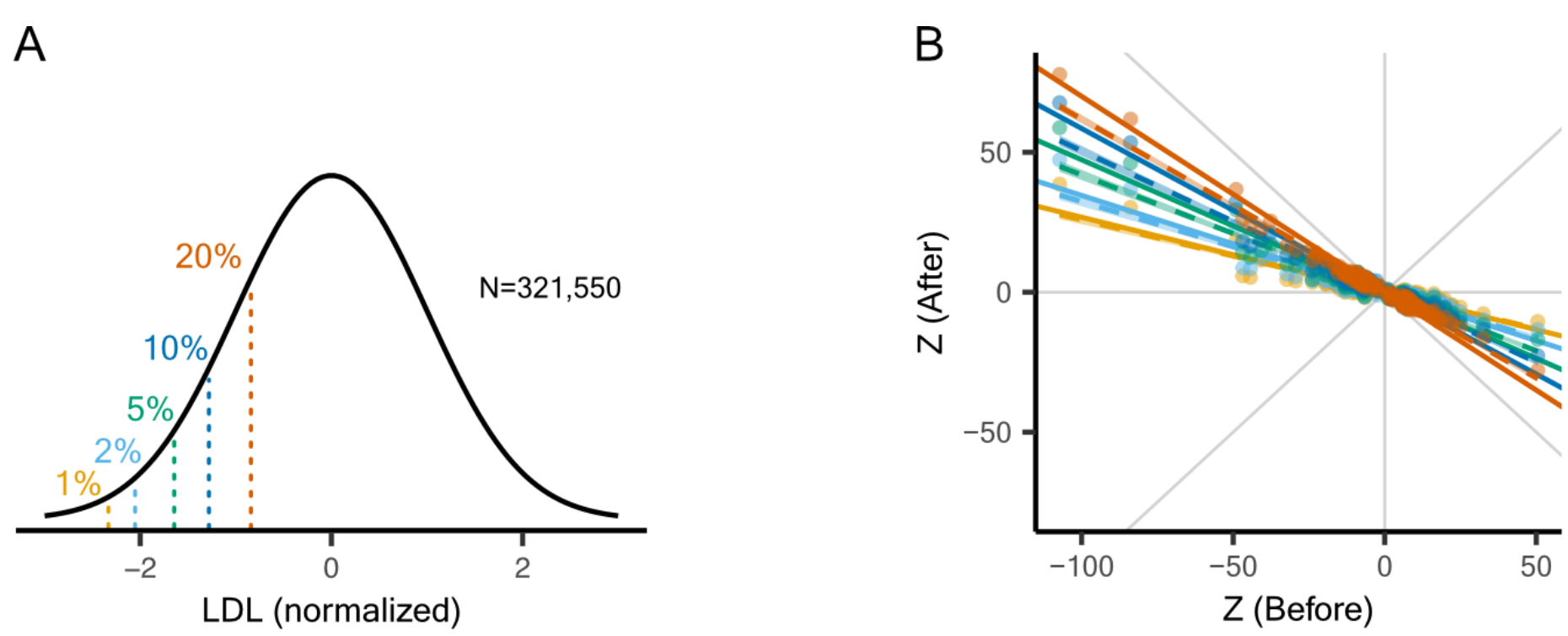

Fig. S19. LDL GWAS power reduction after binarization. ***A)*** *Binarizing LDL levels with varying prevalence in the lower tail.* ***B)*** *Deflation of GWAS hit Z scores following lower-tail binarization of LDL. Different colors correspond to different prevalence used in panel A. Solid lines represent predicted slopes, and dashed lines indicate fitted slopes. Gray lines in the background are* $y = 0$, $x = 0$ *and* $y = \pm x$. *LDL was measured in unrelated White British individuals from the UK Biobank (data field 30780). The original phenotypic distribution was standardized using rank-based inverse normal transformation.*

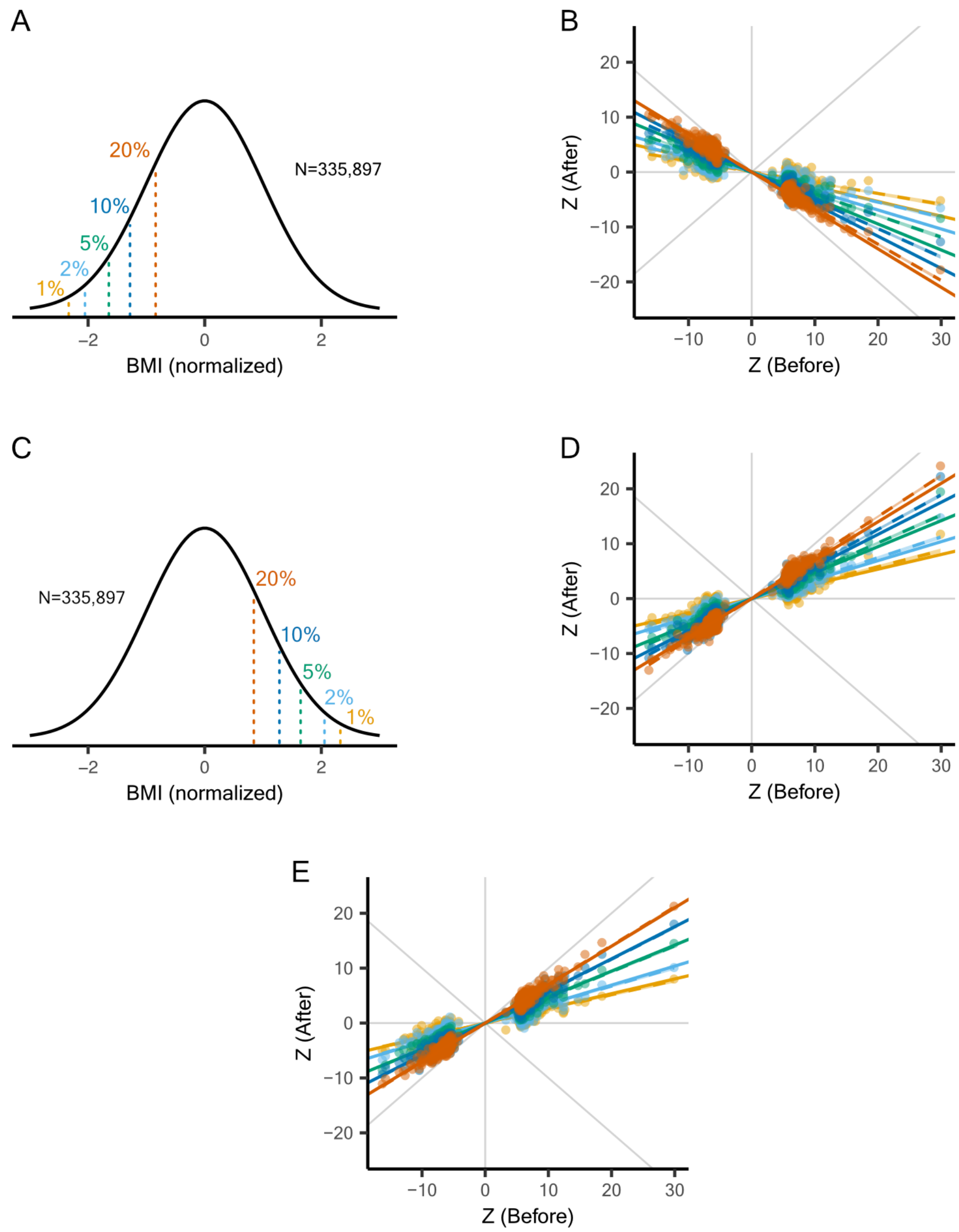

Fig. S20. BMI GWAS power reduction after binarization or downsampling. *Binarizing BMI with varying prevalence in the* ***A)*** *lower tail or the* ***C)*** *upper tail. Deflation of GWAS hit Z scores after* ***B)*** *binarization in the lower tail,* ***D)*** *binarization in the upper tail, or* ***E)*** *downsampling BMI to matched sample sizes. In panels B, D and E, different colors correspond to different prevalence used in panels A and C. Solid lines represent predicted slopes, and dashed lines indicate fitted slopes. Gray lines in the background are* $y = 0$, $x = 0$ *and* $y = \pm x$. *BMI was measured in unrelated White British individuals from the UK Biobank (data field 21001). The original phenotypic distribution was standardized using rank-based inverse normal transformation.*

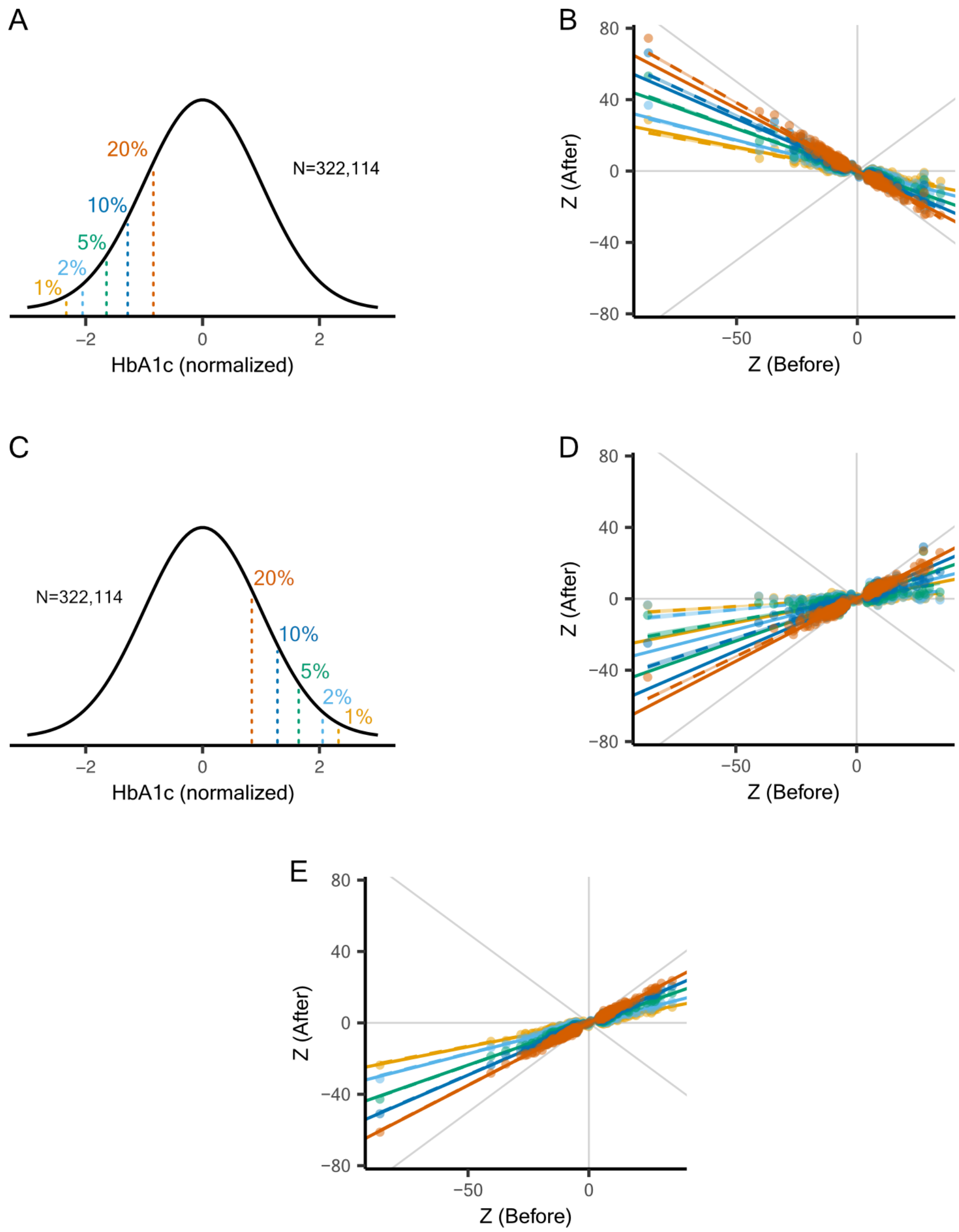

Fig. S21. HbA1c GWAS power reduction after binarization or downsampling. *Binarizing hemoglobin A1C (HbA1c) with varying prevalence in the* ***A)*** *lower tail or the* ***C)*** *upper tail. Deflation of GWAS hit Z scores after* ***B)*** *binarization in the lower tail,* ***D)*** *binarization in the upper tail, or* ***E)*** *downsampling HbA1c to matched sample sizes. In panels B, D and E, different colors correspond to different prevalence used in panels A and C. Solid lines represent predicted slopes, and dashed lines indicate fitted slopes. Gray lines in the background are* $y = 0$, $x = 0$ *and* $y = \pm x$. *HbA1c was measured in unrelated White British individuals from the UK Biobank (data field 30750). The original phenotypic distribution was standardized using rank-based inverse normal transformation.*

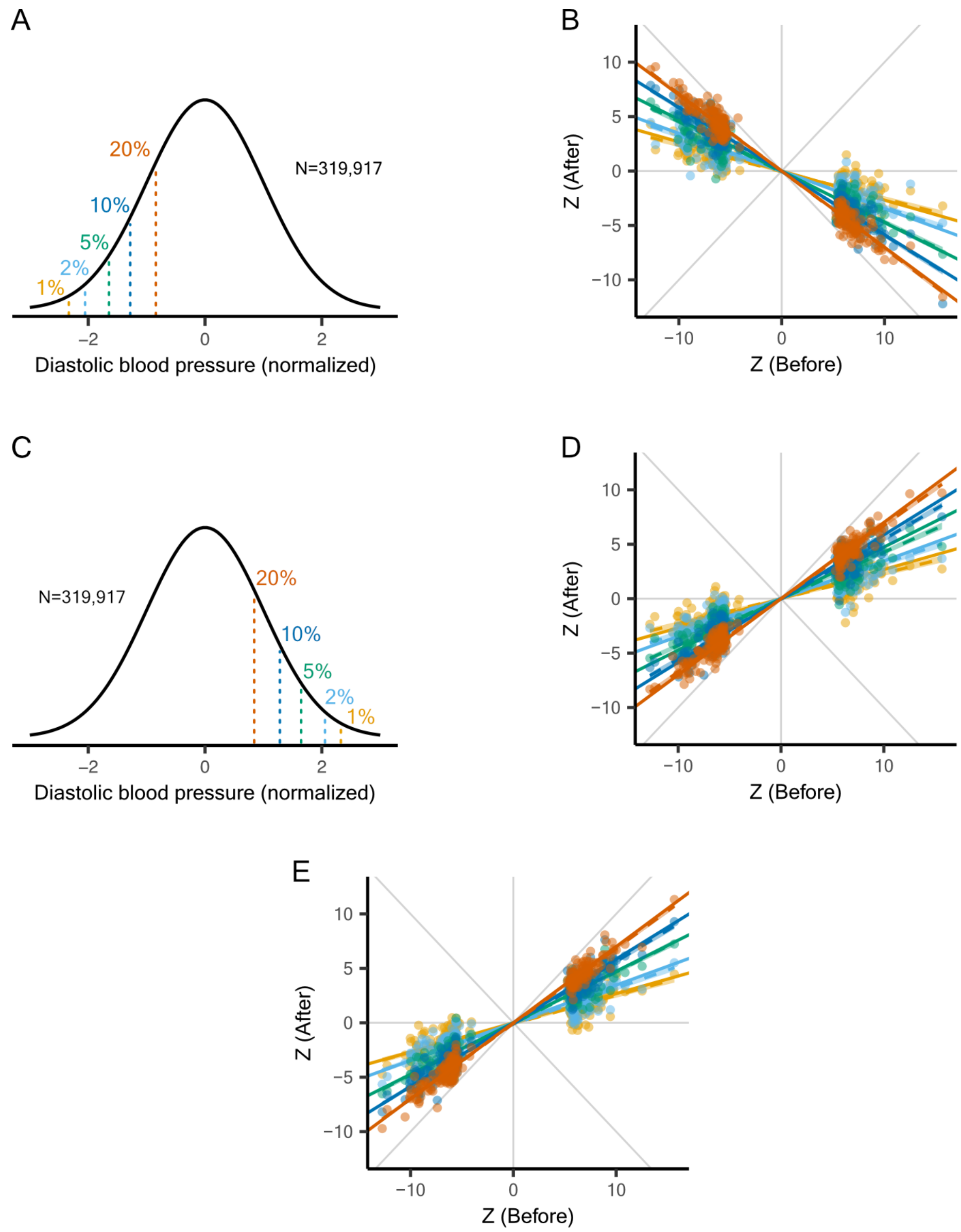

Fig. S22. Diastolic blood pressure GWAS power reduction after binarization or downsampling. *Binarizing diastolic blood pressure with varying prevalence in the* ***A)*** *lower tail or the* ***C)*** *upper tail. Deflation of GWAS hit Z scores after* ***B)*** *binarization in the lower tail,* ***D)*** *binarization in the upper tail, or* ***E)*** *downsampling diastolic blood pressure to matched sample sizes. In panels B, D and E, different colors correspond to different prevalence used in panels A and C. Solid lines represent predicted slopes, and dashed lines indicate fitted slopes. Gray lines in the background are $y = 0$, $x = 0$ and $y = \pm x$. Diastolic blood pressure was measured in unrelated White British individuals from the UK Biobank (data field 4079). The original phenotypic distribution was standardized using rank-based inverse normal transformation.*

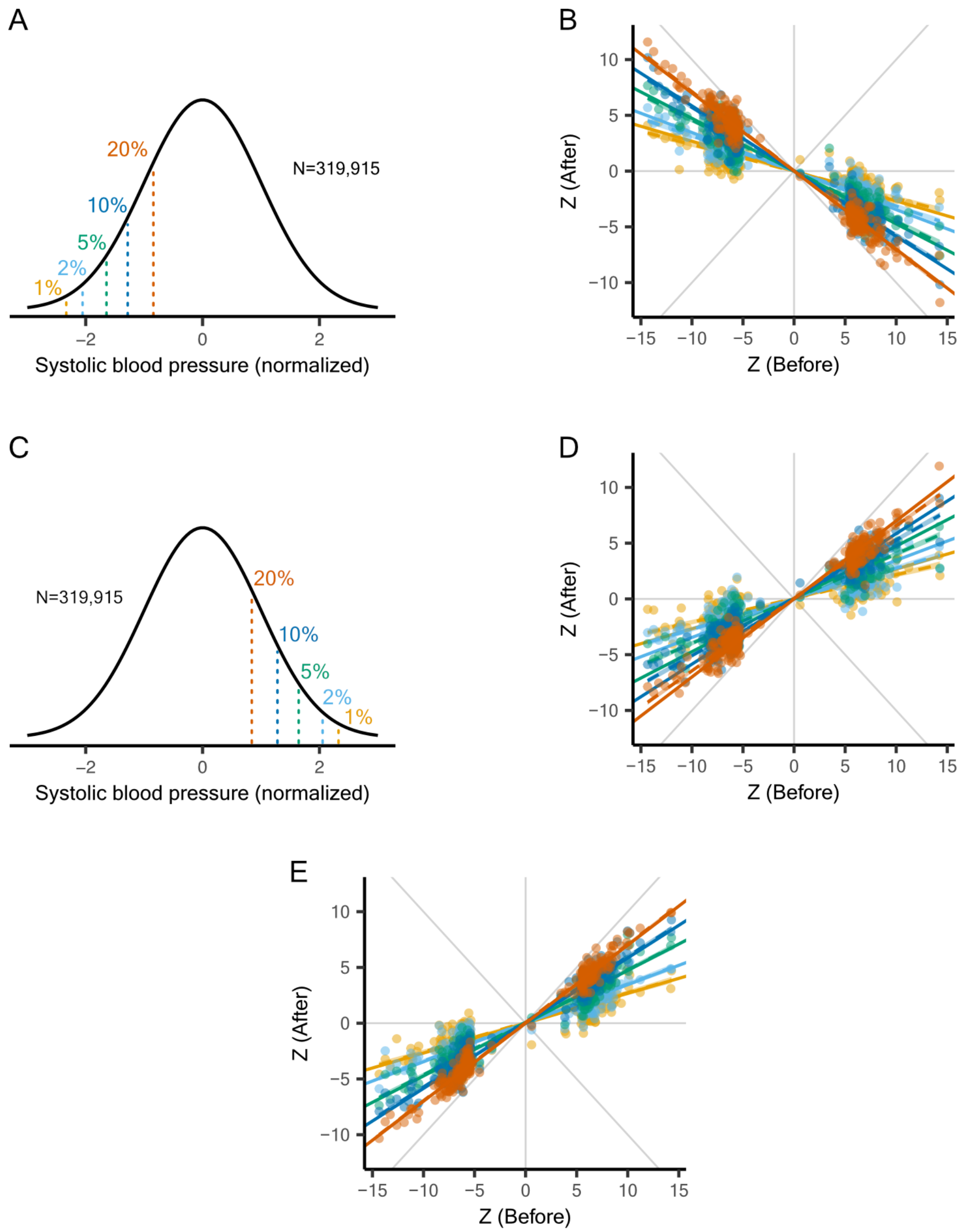

Fig. S23. Systolic blood pressure GWAS power reduction after binarization or downsampling. *Binarizing systolic blood pressure with varying prevalence in the* ***A)*** *lower tail or the* ***C)*** *upper tail. Deflation of GWAS hit Z scores after* ***B)*** *binarization in the lower tail,* ***D)*** *binarization in the upper tail, or* ***E)*** *downsampling systolic blood pressure to matched sample sizes. In panels B, D and E, different colors correspond to different prevalence used in panels A and C. Solid lines represent predicted slopes, and dashed lines indicate fitted slopes. Gray lines in the background are* $y = 0$, $x = 0$ *and* $y = \pm x$. *Systolic blood pressure was measured in unrelated White British individuals from the UK Biobank (data field 4080). The original phenotypic distribution was standardized using rank-based inverse normal transformation.*

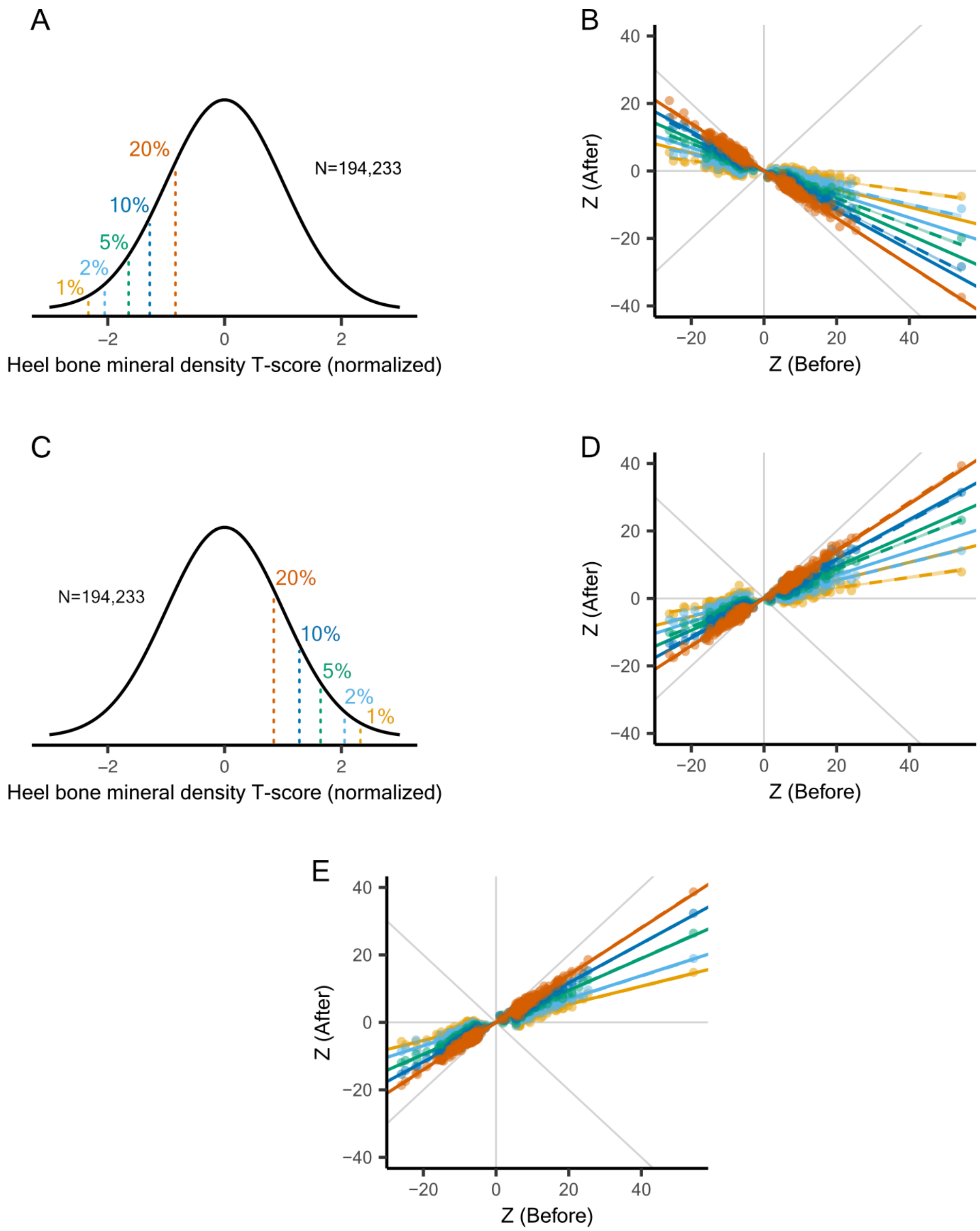

Fig. S24. Heel bone mineral density T-score GWAS power reduction after binarization or downsampling. *Binarizing heel bone mineral density T-score with varying prevalence in the **A)** lower tail or the **C)** upper tail. Deflation of GWAS hit Z scores after **B)** binarization in the lower tail, **D)** binarization in the upper tail, or **E)** downsampling heel bone mineral density T-score to matched sample sizes. In panels B, D and E, different colors correspond to different prevalence used in panels A and C. Solid lines represent predicted slopes, and dashed lines indicate fitted slopes. Gray lines in the background are $y = 0$, $x = 0$ and $y = \pm x$. Heel bone mineral density T-score was measured in unrelated White British individuals from the UK Biobank (data field 78). The original phenotypic distribution was standardized using rank-based inverse normal transformation.*

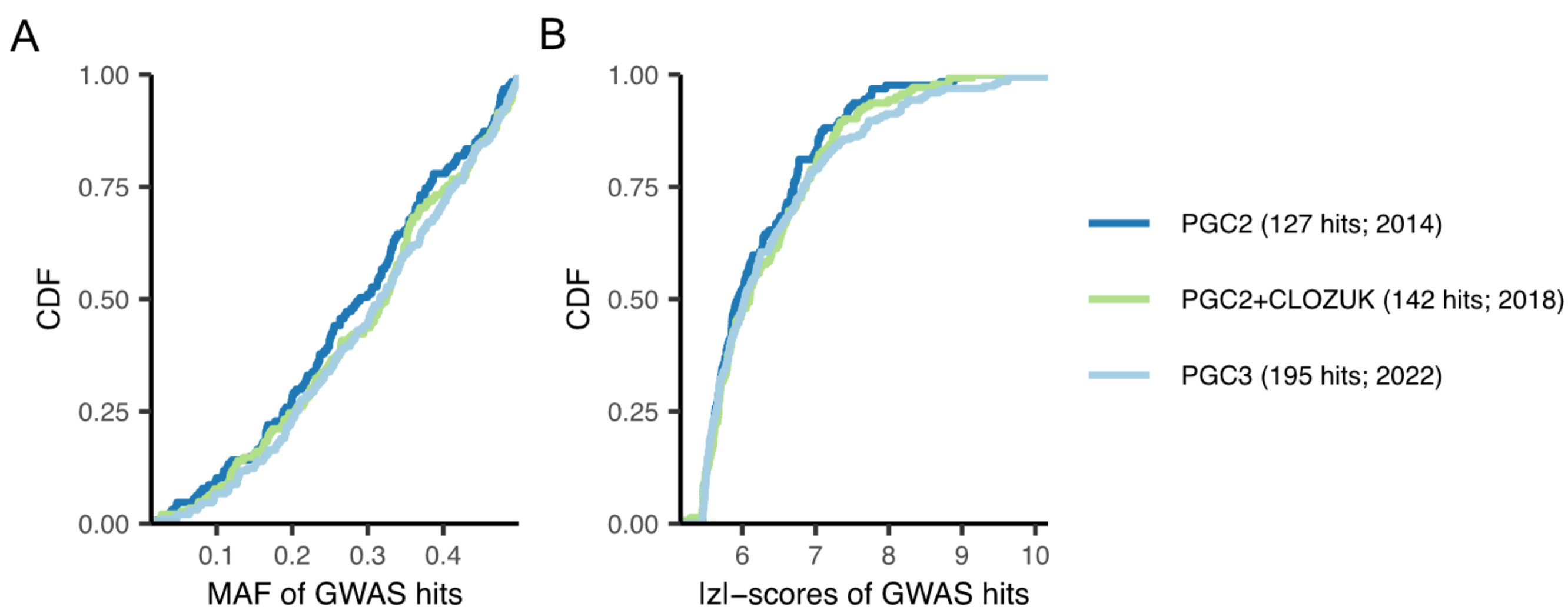

Fig. S25. Comparing the approximately independent hits from three schizophrenia GWAS. ***A)*** *CDF curves of MAF among GWAS hits.* ***B)*** *CDF curves of absolute z-scores among GWAS hits. More details of the three GWAS (9, 14, 15) are provided in Table S3. Approximately independent hits for PGC2 were taken from Supplementary Table 2 of (14), whereas COJO hits for PGC2+CLOZUK and PGC3 were identified following procedures described in the Materials and Methods section.*

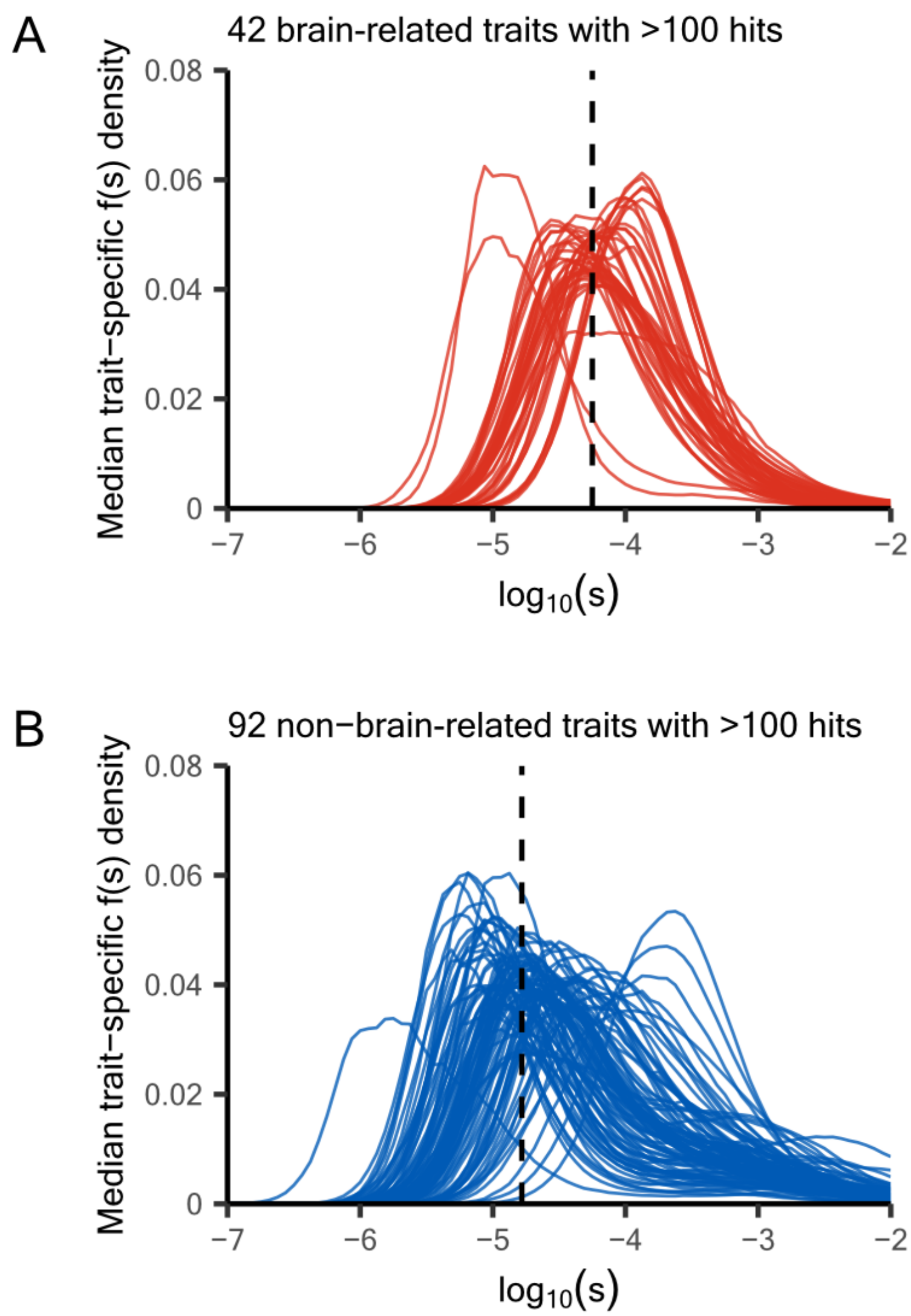

Fig. S26. Median trait-specific distributions of selection coefficients inferred from 134 traits with at least 100 GWAS hits, with traits grouped into *A)* *brain-related* versus *B)* *non-brain-related* traits. *Vertical line in each plot corresponds to the median mode.*

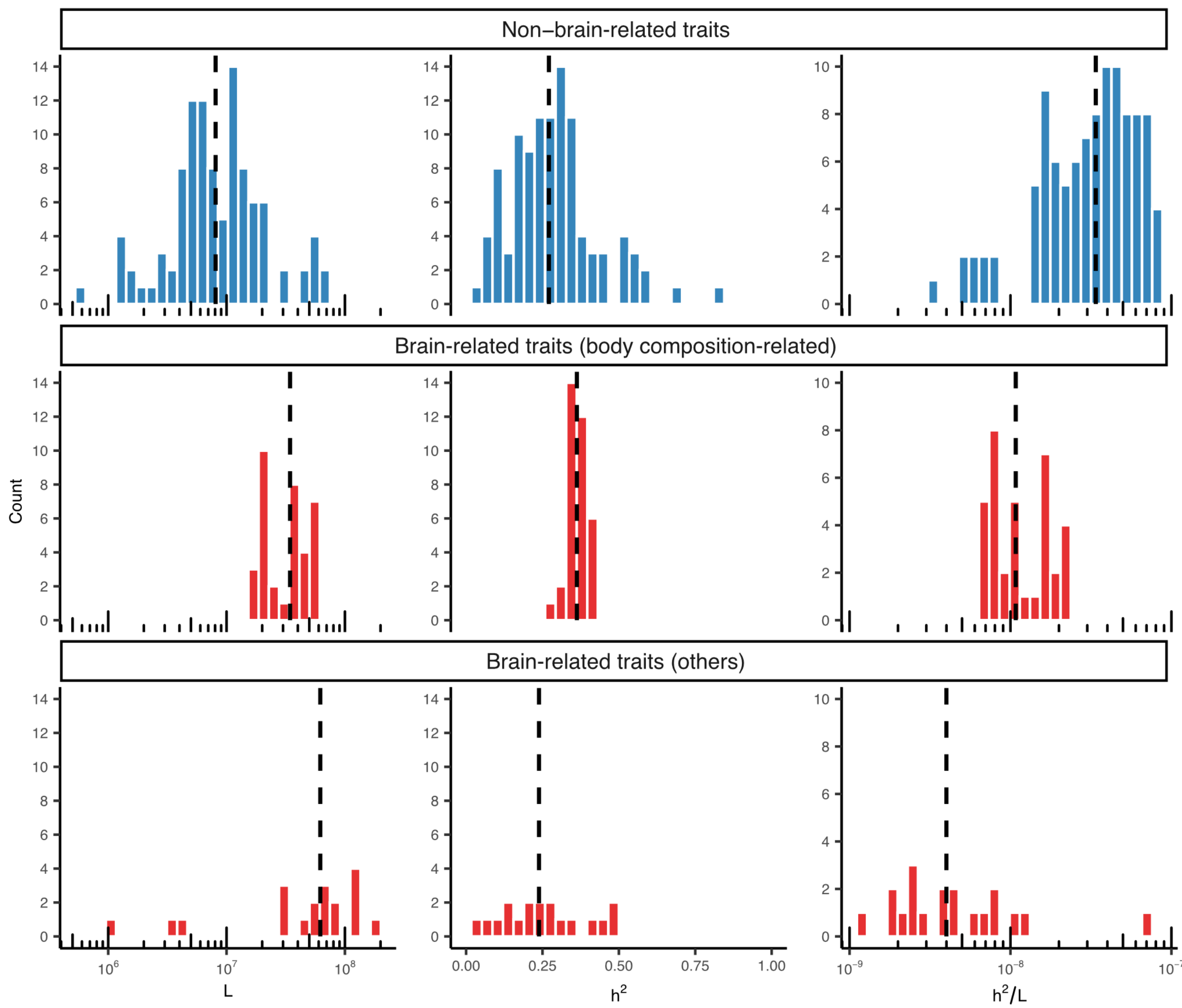

Fig. S27. Re-plotting the estimates in Figure 4D as histograms to highlight distribution shifts. *Dashed vertical lines indicate the median values in each subplot. Brain-related traits are further divided into body composition-related traits and other brain-related traits. Body composition-related traits have L and h^2/L estimates that fall between those of non–brain-related traits and traits primarily influenced by the CNS alone.*

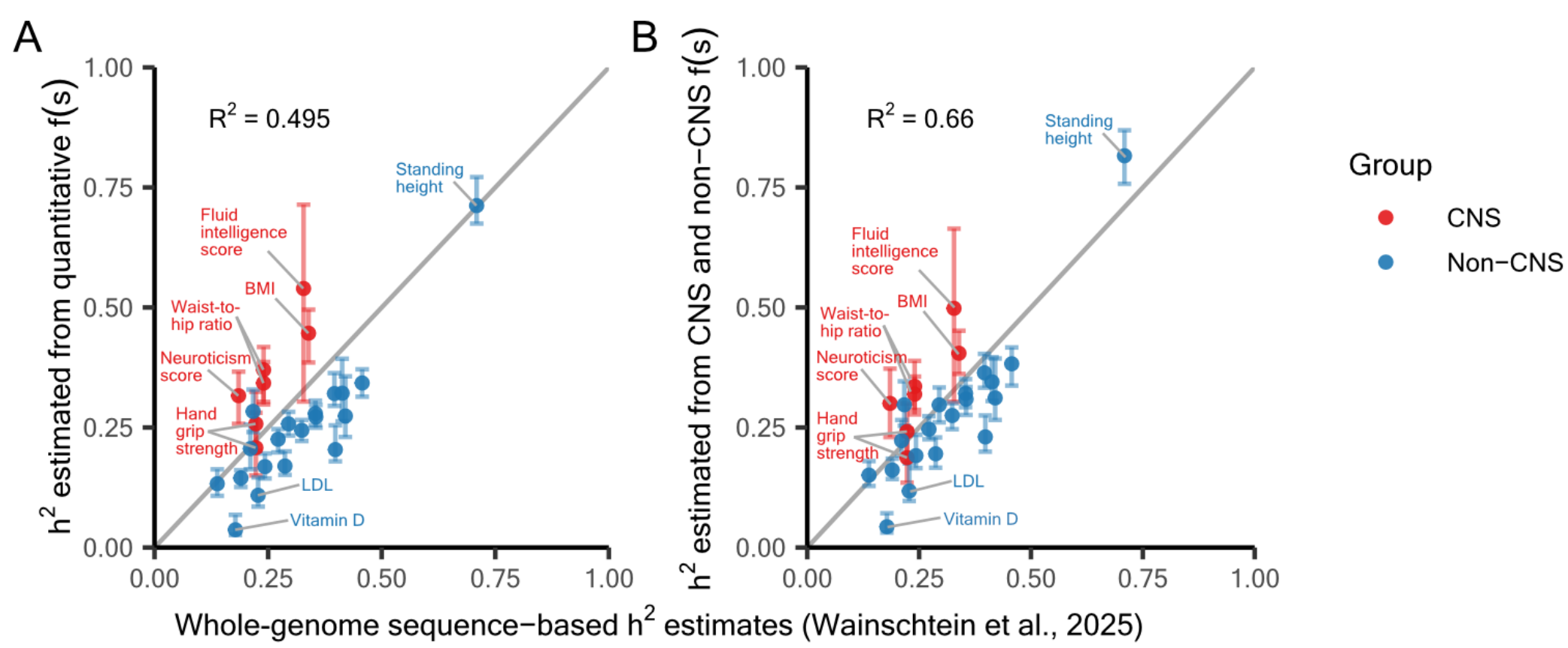

Fig. S28. Comparing heritability estimates obtained in this study with recently reported whole-genome sequence-based heritability estimates (h^2_{WGS}). ***A)*** *Comparing h^2_{WGS} with h^2 obtained jointly with quantitative $f(s)$, as shown in Figure 4B.* ***B)*** *Comparing h^2_{WGS} with h^2 obtained jointly with brain-related or non-brain-related $f(s)$, as shown in Figure 4C. Wainschtein et al. (16) estimated h^2_{WGS} in 347,630 unrelated individuals from the UK Biobank with GREML-LDMS.*

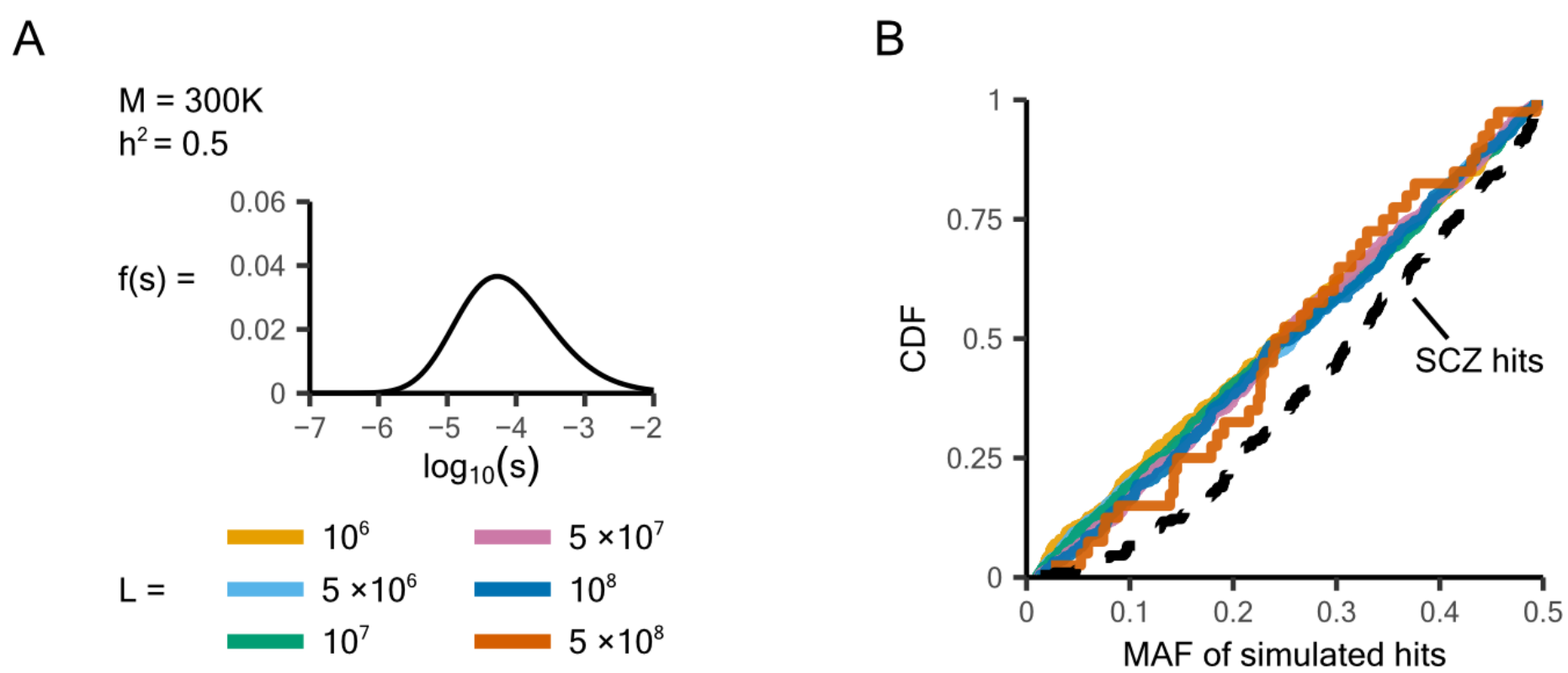

Fig. S29. Variation in MAF distributions of GWAS hits cannot be recovered by changing L. ***A)*** *Same simulation setup as in Figure 5A.* ***B)*** *Varying L does not change the MAF distribution of GWAS hits. The black dashed line shows real schizophrenia GWAS hits for reference.*

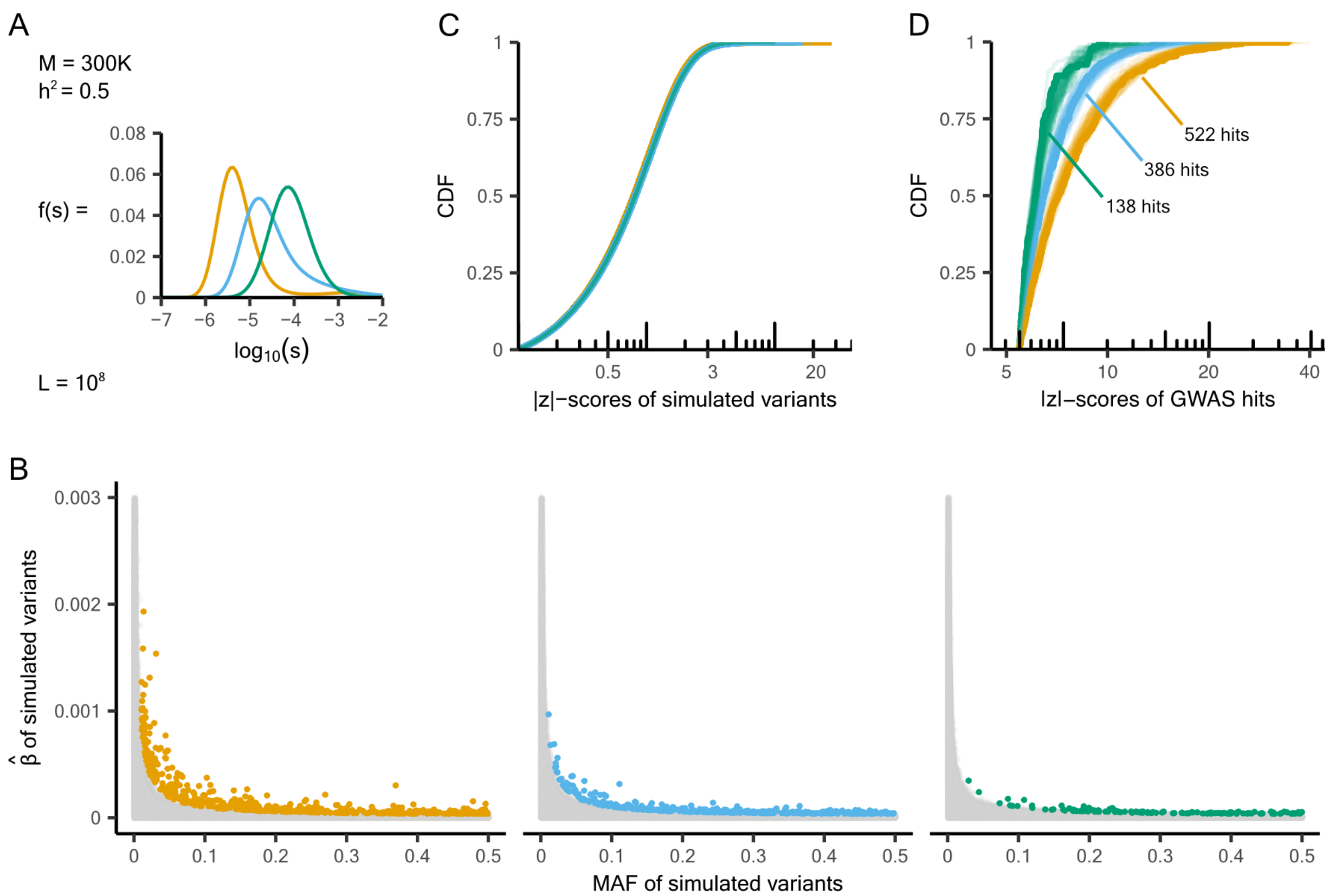

Fig. S30. Impact of GWAS ascertainment on simulations under different $f(s)$ distributions. ***A)*** *Same simulation setup as in Figure 5D.* ***B)*** *The GWAS ascertainment process. Variants with MAF $> 1\%$ and $|Z| > 5.45$ are discovered as hits and colored according to the $f(s)$ distribution from which they were simulated. Unselected variants are shown in grey.* ***C)*** *Without GWAS ascertainment, the CDFs of $|Z|$ scores for variants simulated under different $f(s)$ distributions differ in the tails.* ***D)*** *After GWAS ascertainment, stronger $f(s)$ produces steeper GWAS hit $|Z|$ score CDFs and fewer hits.*

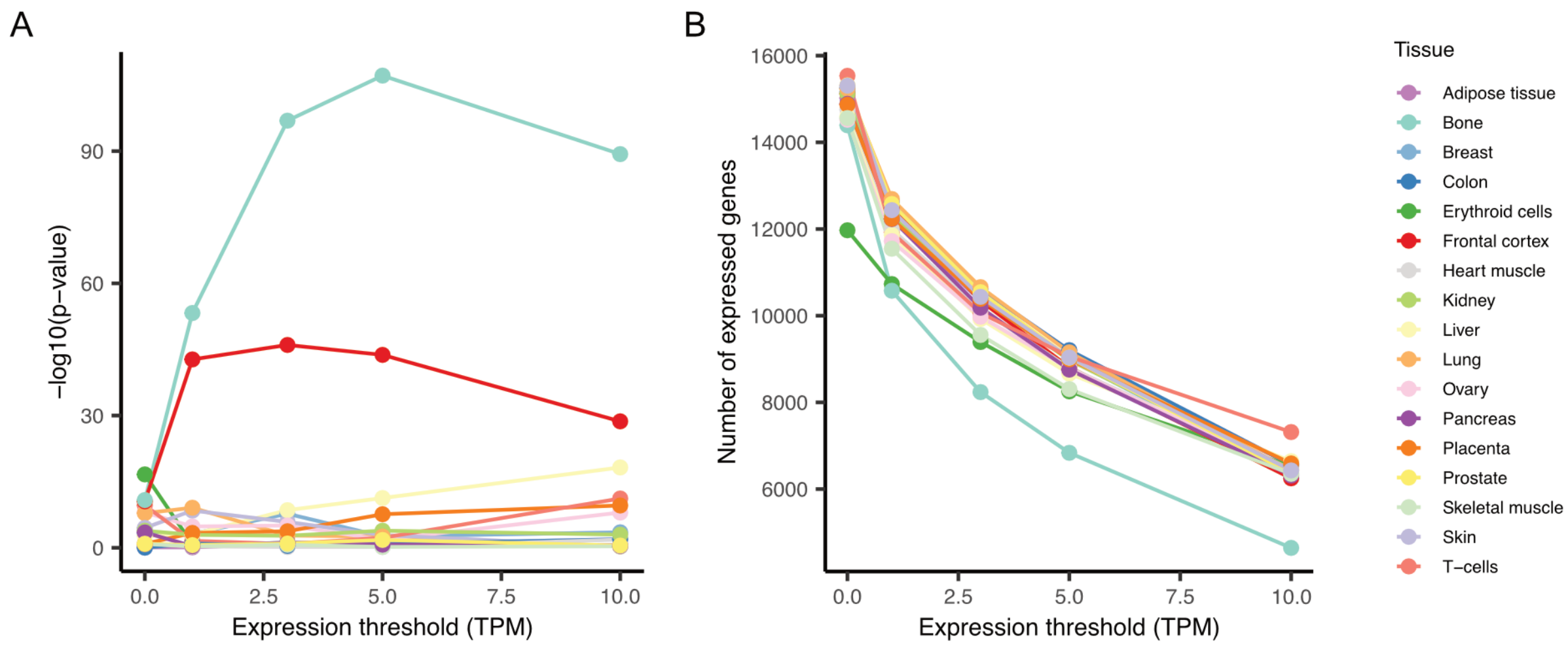

Fig. S31. Brain expression status strongly predicts a gene's constraint estimate, s_{het}**.** ***A)*** *P-values from multiple regression models, where* $\log_{10}(s_{het})$*, estimated from a baseline model trained without any gene features, is regressed on expression status across 17 tissues. A gene is classified as "expressed" in a given tissue if its transcripts per million (TPM) value exceeds a threshold of 0, 1, 3, 5, or 10.* ***B)*** *While "frontal cortex" and "bone" appear to be the strongest predictors in panel A, genes are generally lowly expressed in the bone, and increasing the TPM cutoff rapidly excludes a large number of genes. In contrast, expression levels in the frontal cortex are more comparable to those in other tissues, indicating that its signal in panel A is not driven by a more rapid loss of genes at higher TPM thresholds. Gene expression values across tissues were curated in (17), with data extracted from the Human Protein Atlas (18).*

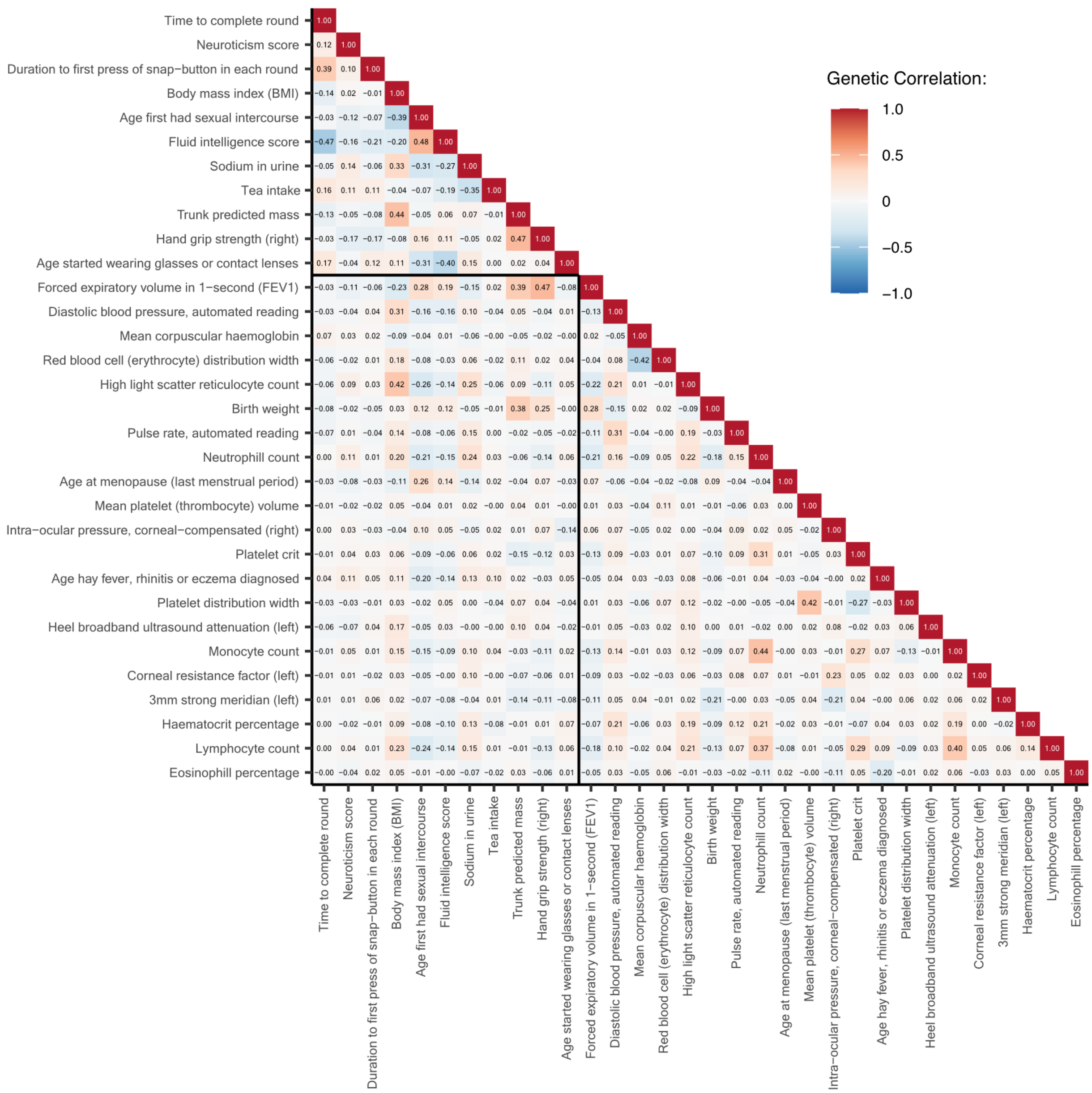

Fig. S32. Heatmap of pairwise genetic correlations across 32 independent traits. *The top 11 traits are CNS traits.*

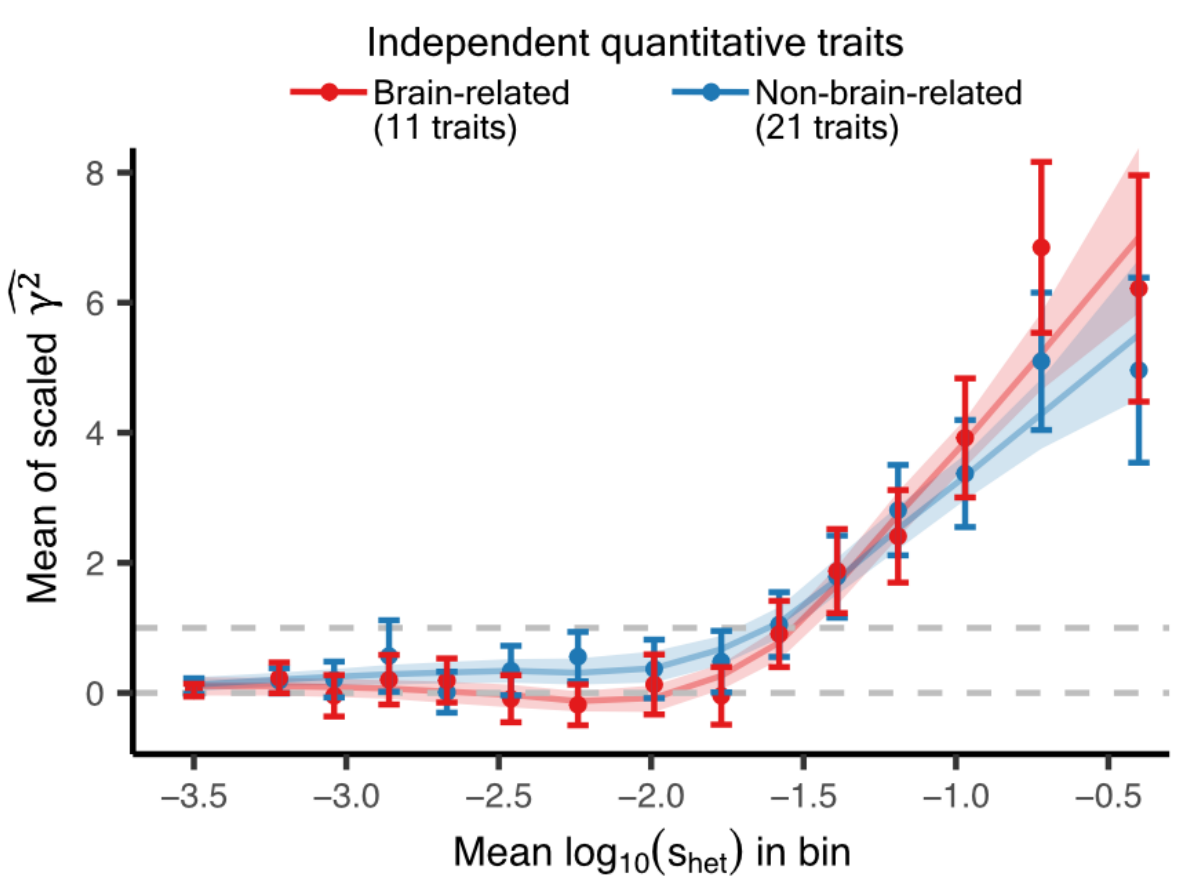

Fig. S33. Mean squared genic effects on independent brain-related versus non-brain-related traits. *Genes are grouped into 15 quantiles of s_{het} estimates (19). The estimated $\widehat{\gamma^2}$ values are scaled so that the mean across all gene-trait pairs in the tenth bin equals one. We applied a MAF $< 1\%$ filter. All estimates are shown with a 95% confidence interval. We bootstrapped genes at the trait level and showed the mean and 95% confidence intervals of the fitted LOESS curves.*

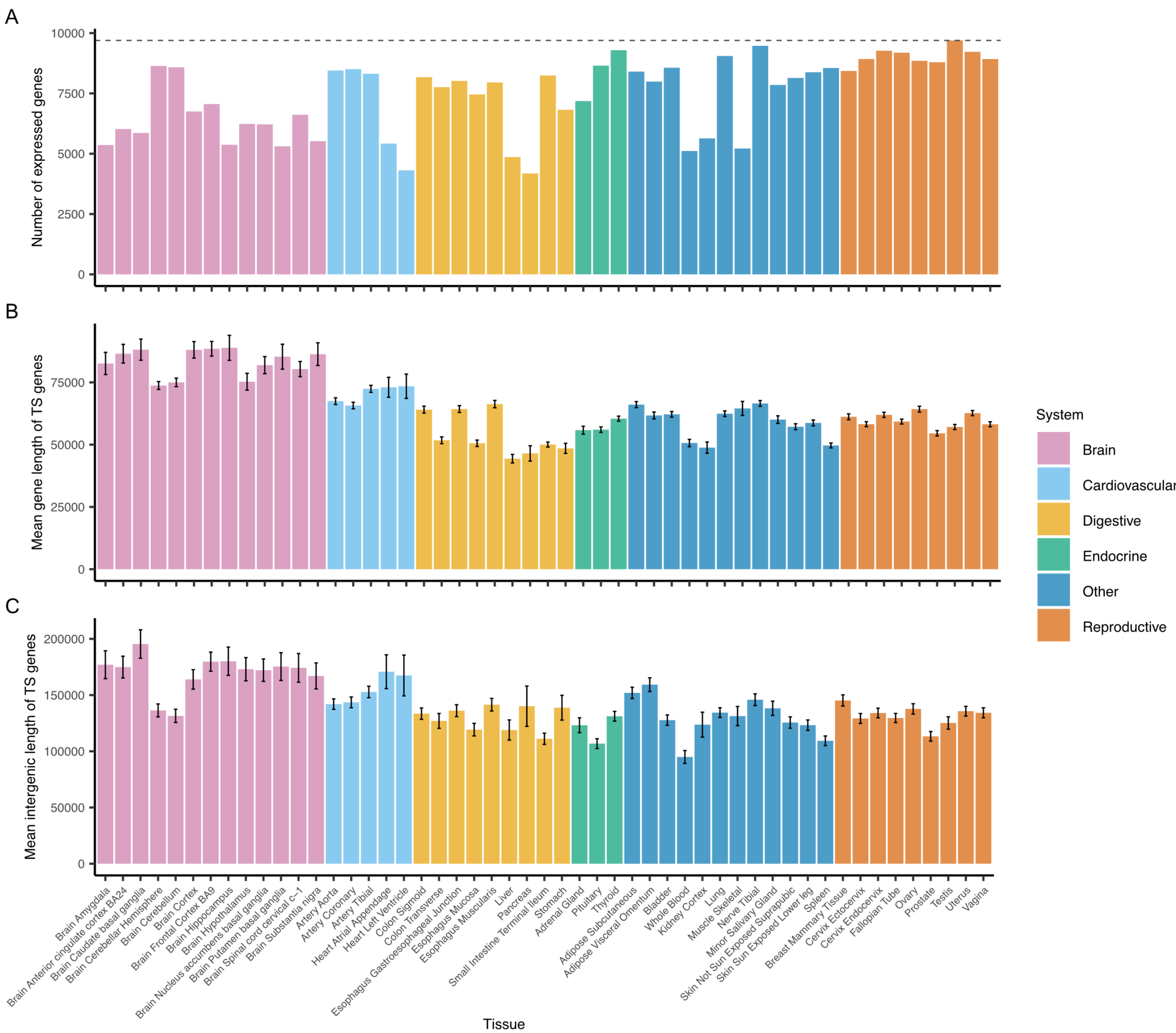

Fig. S34. Genes expressed in the brain tend to be longer and have longer intergenic lengths. ***A)*** *Brain tissues do not express more genes than other tissues. Median gene expression levels of bulk tissues were obtained from the GTEx portal, v11 (20). A gene was considered "expressed" using a transcripts per million (TPM) cutoff of 10. The horizontal line was drawn at testis, the tissue with the highest number of expressed genes.* ***B)*** *Tissue-specific (TS) genes in the brain are, on average, longer than TS genes in other tissues. An expressed gene was classified as TS if its expression level in a given tissue was greater than the mean expression across all other tissues.* ***C)*** *Brain-specific genes also have longer intergenic lengths on average. Intergenic length was defined as the sum of the distances in bp from a gene to its adjacent genes or to the nearest chromosome ends.*

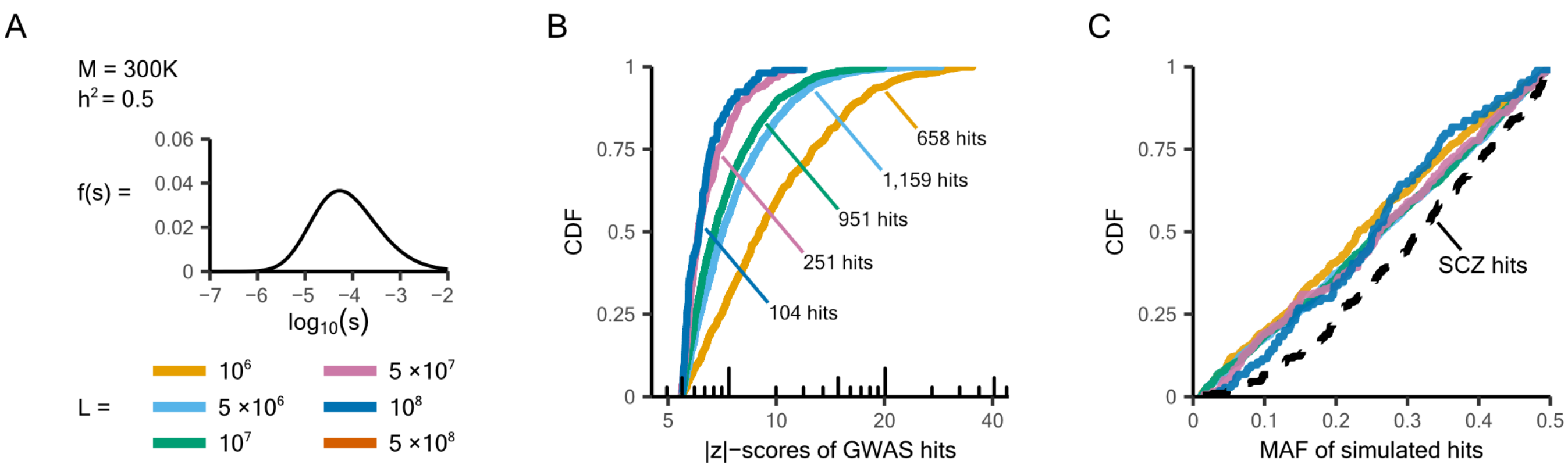

Fig. S35. Assuming no pleiotropy for variant effects fails to recapitulate the observed variation in MAF distributions of GWAS hits. ***A)*** *Simulating under a modified Simons model that assumes no pleiotropy (Materials and Methods). The rest of the simulation setup is the same as in Figure 5A and S29.* ***B)*** *Larger L values lead to narrower distributions of GWAS hit $|z|$-scores. Simulating with a mutational target size of 5×10^8 did not generate any GWAS hits.* ***C)*** *Varying L does not change the MAF distribution of GWAS hits. The black dashed line shows real schizophrenia GWAS hits for reference.*

Supplementary Tables

Table S1. Details of 13 complex diseases and disorders included in this study, with commonly used abbreviations, source of case-control GWAS summary statistics, LDSC intercepts obtained from the log files of our S-LDSC analyses, and the number of genome-wide independent associations identified with GCTA-COJO (21) (Materials and Methods).

Disease/Disorder (Abbreviation)	GWAS source	LDSC intercept	Number of hits
Alzheimer's disease (AD)	(22)	1.068	62
Attention deficit hyperactivity disorder (ADHD)	(23)	1.045	24
Bipolar disorder (BIP)	(24)	1.065	74
Breast cancer (BrCa)	(25)	1.091	202
Coronary artery disease (CAD)	(26)	0.96	217
Inflammatory bowel disease (IBD)	(27)	1.096	127
Major depression (MD)	(28)	1.092	249
Multiple sclerosis (MS)	(29)	0.99	87
Parkinson's disease (PD)	(30)	0.99	22
Prostate cancer (PrCa)	(31)	1.039	134
Rheumatoid arthritis (RA)	(32)	1.053	97
Schizophrenia (SCZ)	(9)	1.085	196
Type 2 Diabetes (T2D)	(33)	1.077	138

Table S2. Key parameters for complex diseases and disorders included in this study. M**: total sample size in the case-control study, including both cases and controls;** ω**: sample prevalence;** K**: population prevalence commonly used or estimated for the European population, taken from the GWAS source paper unless annotated with *;** N'**: effective sample size required on the liability scale to reach equivalent power. We calculated** $M\omega(1-\omega)$ **with reported** M **and** ω**. Details on how we estimated** $\widehat{M\omega(1-\omega)}$ **and N' can be found in the Materials and Methods section.**

Disease/Disorder	Reported M	Reported ω	$M\omega(1-\omega)$	$\widehat{M\omega(1-\omega)}$	K	N'
AD	487,511	17.6%	70,701	29,699	5% (34)*	140,016
ADHD	225,543	17.2%	32,121	22,449	5% (23)	105,835
BIP	840,309	7.1%	55,426	35,255	2% (24)	215,143
BrCa	247,173	54.0%	61,398	52,115	2% (35)*	318,030
CAD	1,165,690	15.6%	153,479	78,272	7% (26)	332,959
IBD	59,957	41.8%	14,586	12,836	0.5% (36)*	108,431
MD	2,000,702	20.6%	327,243	244,161	15% (28)	816,506
MS	115,803	41.0%	28,013	7,327	0.1% (29)	83,236
PD	482,730	7.0%	31,426	4,018	0.5% (30)	33,945
PrCa	140,254	56.4%	34,489	29,087	2% (35)*	177,504
RA	97,173	23.0%	17,209	8,159	0.5% (32)	68,921
SCZ	130,644	40.9%	31,579	26,529	1% (9)	192,273
T2D	659,316	9.5%	56,685	32,547	10% (33)	123,757

Table S3. Comparison of three schizophrenia GWAS of European ancestry: PGC2 (14), PGC2+CLOZUK (15), and PGC3 (9). The three samples are approximately nested, with each larger study incorporating the previous cohort. CLOZUK: patients with treatment-resistant schizophrenia in the UK taking clozapine; 1000G: the 1000 Genomes project; HRC: the Haplotype Reference Consortium.

	**PGC2 (2014)**	**PGC2+CLOZUK (2018)**	**PGC3 (2022)**
Cases	$36{,}989$	$40{,}675$	$53{,}386$
Sample prevalence	24.6%	38.6%	40.9%
CLOZUK cases	$5{,}531$	$11{,}260$	$11{,}260$
Imputation panel	1000G Phase 1 ($N = 2{,}186$)	1000G Phase 3 + UK10K	HRC ($N = 54{,}330$)
Quality control	INFO> 0.6, MAF> 0.01	INFO> 0.6, MAF> 0.01	INFO> 0.6, MAF> 0.01
PC correction	First 10 PCs	First 5 + significant PCs	First 4 + significant PCs
LDSC intercept	1.066	1.075	1.08

Table S4. Evaluating model fit of the normal distribution, the alpha model, and the Simons model. For each trait-model combination, we conducted Kolmogorov–Smirnov (KS) tests to assess whether the residual p-values follow a uniform distribution. We then applied the Benjamini-Hochberg (BH) procedure to the KS p-values for each model to control the false discovery rate at 0.05.

	Normal distribution		Alpha model		Trait-specific f(s)	
**Disease**	**KS p-value**	**BH decision**	**KS p-value**	**BH decision**	**KS p-value**	**BH decision**
BrCa	9.521×10^{-10}	**Reject**	4.666×10^{-8}	**Reject**	0.641	Accept
CAD	1.756×10^{-12}	**Reject**	6.384×10^{-8}	**Reject**	0.242	Accept
IBD	3.992×10^{-3}	**Reject**	0.201	Accept	0.904	Accept
MD	0.765	Accept	0.764	Accept	0.538	Accept
SCZ	6.797×10^{-3}	**Reject**	0.0164	**Reject**	0.0498	Accept
T2D	5.133×10^{-9}	**Reject**	1.405×10^{-9}	**Reject**	0.191	Accept

References

1. YB Simons, et al., Simple scaling laws control the genetic architectures of human complex traits. *PLoS biology* **23**, e3003402 (2025).
2. X Zhu, M Stephens, Bayesian large-scale multiple regression with summary statistics from genome-wide association studies. *The annals applied statistics* **11**, 1561 (2017).
3. AC Gillett, E Vassos, CM Lewis, Transforming summary statistics from logistic regression to the liability scale: application to genetic and environmental risk scores. *Hum. Hered.* **83**, 210–224 (2019).
4. T Wu, PC Sham, On the transformation of genetic effect size from logit to liability scale. *Behav. Genet.* **51**, 215–222 (2021).
5. GM McMahon, SS Waikar, Biomarkers in nephrology. *Am. journal kidney diseases: official journal Natl. Kidney Foundation* **62**, 165 (2013).
6. WC de Groat, D Griffiths, N Yoshimura, Neural control of the lower urinary tract. *Compr. Physiol.* **5**, 327 (2015).
7. D Speed, G Hemani, MR Johnson, DJ Balding, Improved heritability estimation from genome-wide snps. *The Am. J. Hum. Genet.* **91**, 1011–1021 (2012).
8. T Berisa, JK Pickrell, Approximately independent linkage disequilibrium blocks in human populations. *Bioinformatics* **32**, 283 (2015).
9. V Trubetskoy, et al., Mapping genomic loci implicates genes and synaptic biology in schizophrenia. *Nature* **604**, 502–508 (2022).
10. HK Finucane, et al., Partitioning heritability by functional annotation using genome-wide association summary statistics. *Nat. Genet.* **47**, 1228–1235 (2015).
11. HK Finucane, et al., Heritability enrichment of specifically expressed genes identifies disease-relevant tissues and cell types. *Nat. genetics* **50**, 621–629 (2018).
12. Neale lab - UK Biobank GWAS summary statistics (http://www.nealelab.is/uk-biobank/) (2018) [Online; accessed 1-October-2022].
13. LJ Howe, et al., Within-sibship genome-wide association analyses decrease bias in estimates of direct genetic effects. *Nat. genetics* **54**, 581–592 (2022).
14. C Pantelis, et al., Biological insights from 108 schizophrenia-associated genetic loci. *Nature* **511**, 421–427 (2014).
15. AF Pardiñas, et al., Common schizophrenia alleles are enriched in mutation-intolerant genes and in regions under strong background selection. *Nat. genetics* **50**, 381–389 (2018).
16. P Wainschtein, et al., Estimation and mapping of the missing heritability of human phenotypes. *Nature* pp. 1–9 (2025).
17. JP Spence, et al., Specificity, length and luck drive gene rankings in association studies. *Nature* pp. 1–8 (2025).
18. M Uhlén, et al., Tissue-based map of the human proteome. *Science* **347**, 1260419 (2015).
19. T Zeng, JP Spence, H Mostafavi, JK Pritchard, Bayesian estimation of gene constraint from an evolutionary model with gene features. *Nat. Genet.* **56**, 1632–1643 (2024).
20. The Genotype-Tissue Expression (GTEx) Portal, v11 (https://gtexportal.org/) (2025) [Online; accessed 1-June-2026].
21. J Yang, et al., Conditional and joint multiple-snp analysis of gwas summary statistics identifies additional variants influencing complex traits. *Nat. genetics* **44**, 369–375 (2012).
22. C Bellenguez, et al., New insights into the genetic etiology of alzheimer's disease and related dementias. *Nat. genetics* **54**, 412–436 (2022).
23. D Demontis, et al., Genome-wide analyses of adhd identify 27 risk loci, refine the genetic architecture and implicate several cognitive domains. *Nat. genetics* **55**, 198–208 (2023).
24. KS O'Connell, et al., Genomics yields biological and phenotypic insights into bipolar disorder. *Nature* pp. 1–12 (2025).
25. H Zhang, et al., Genome-wide association study identifies 32 novel breast cancer susceptibility loci from overall and subtype-specific analyses. *Nat. genetics* **52**, 572–581 (2020).
26. KG Aragam, et al., Discovery and systematic characterization of risk variants and genes for coronary artery disease in over a million participants. *Nat. genetics* **54**, 1803–1815 (2022).
27. KM De Lange, et al., Genome-wide association study implicates immune activation of multiple integrin genes in inflammatory bowel disease. *Nat. genetics* **49**, 256–261 (2017).
28. MJ Adams, et al., Trans-ancestry genome-wide study of depression identifies 697 associations implicating cell types and pharmacotherapies. *Cell* **188**, 640–652 (2025).
29. IMSG Consortium*†, ANZgene, IIBDGC, WTCCC2, Multiple sclerosis genomic map implicates peripheral immune cells and microglia in susceptibility. *Science* **365**, eaav7188 (2019).
30. MA Nalls, et al., Identification of novel risk loci, causal insights, and heritable risk for parkinson's disease: a meta-analysis of genome-wide association studies. *The Lancet Neurol.* **18**, 1091–1102 (2019).
31. FR Schumacher, et al., Association analyses of more than 140,000 men identify 63 new prostate cancer susceptibility loci. *Nat. genetics* **50**, 928–936 (2018).
32. K Ishigaki, et al., Multi-ancestry genome-wide association analyses identify novel genetic mechanisms in rheumatoid arthritis. *Nat. genetics* **54**, 1640–1651 (2022).
33. A Xue, et al., Genome-wide association analyses identify 143 risk variants and putative regulatory mechanisms for type 2 diabetes. *Nat. communications* **9**, 2941 (2018).
34. H Niu, I Álvarez-Álvarez, F Guillén-Grima, I Aguinaga-Ontoso, Prevalence and incidence of alzheimer's disease in europe:

A meta-analysis. *ıa(English Ed.* **32**, 523–532 (2017).

35. R De Angelis, et al., Complete cancer prevalence in europe in 2020 by disease duration and country (eurocare-6): a population-based study. *The Lancet Oncol.* **25**, 293–307 (2024).
36. JZ Liu, et al., Association analyses identify 38 susceptibility loci for inflammatory bowel disease and highlight shared genetic risk across populations. *Nat. genetics* **47**, 979–986 (2015).
